# Supplementary material for: The integrated stress response suppresses PINK1-dependent mitophagy by preserving mitochondrial import efficiency
Source: Nat Commun. 2026 Apr 9;17:4838. doi: 10.1038/s41467-026-71630-6 (PMC13223273; doi:10.1038/s41467-026-71630-6)
Supplement: Supplementary file 1 — Supplementary Information [file 41467_2026_71630_MOESM1_ESM.pdf]

Supplementary Fig. 1

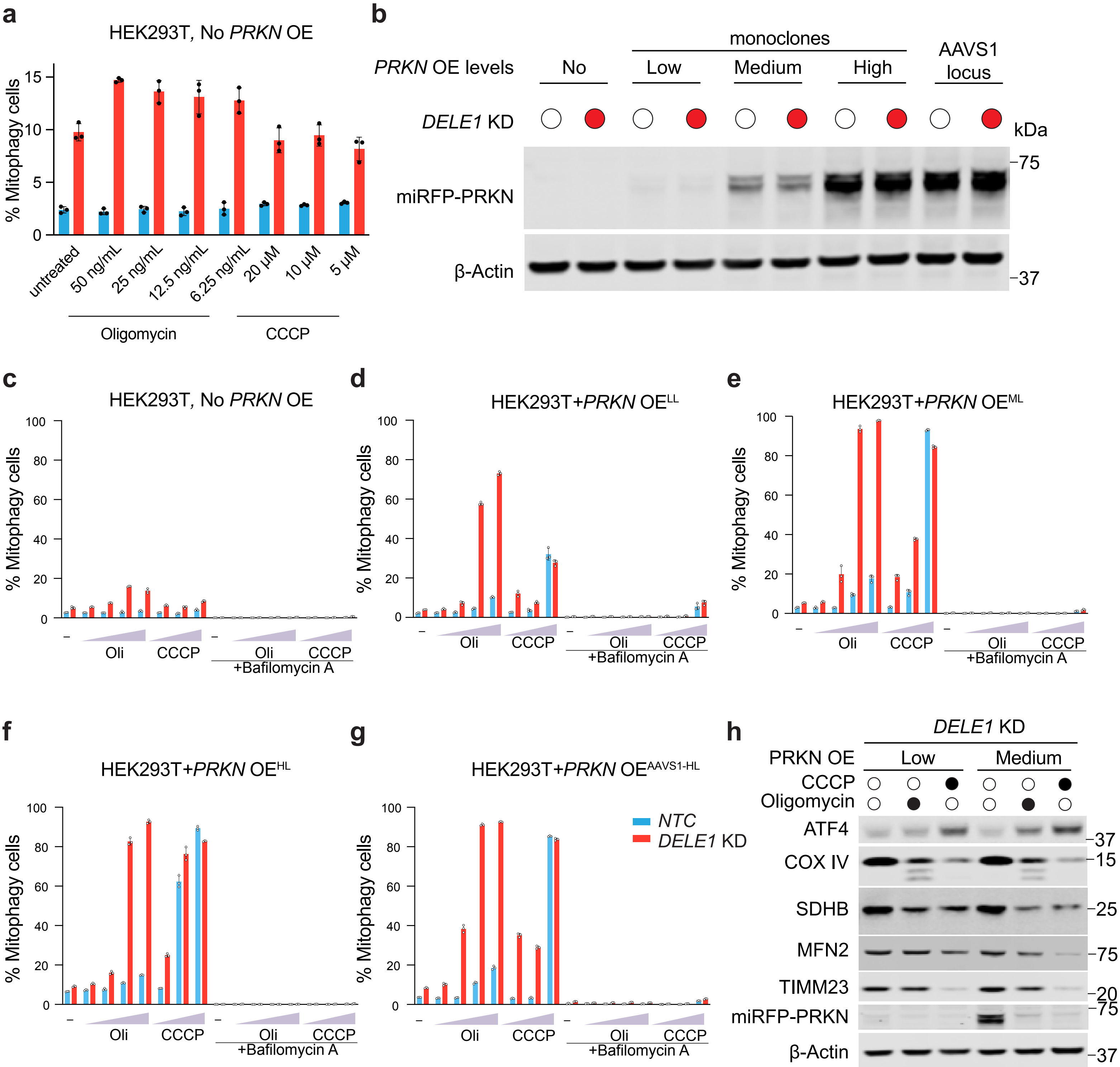

Supplementary Fig.1. Mitophagy levels are positively correlated with the expression levels of *PRKN* in HEK293T cells. a. Mitophagy was measured in *NTC* and *DELE1* KD cells without *PRKN* overexpression. These cells were treated with oligomycin at concentrations ranging from 6.25 to 50 ng/mL, and CCCP at concentrations ranging from 5 to 20 μM. **b.** Immunoblots of PRKN in cells with expression of *PRKN* at different levels. β-actin serves as the loading control. **c-g**. HEK293T *mtKeima* reporter cells without overexpression (OE) of *PRKN*, with a low level of PRKN (*PRKN* OE^{LL}), a medium level (*PRKN* OE^{ML}) or high levels (*PRKN* OE^{HL} and *PRKN* $OE^{AAVS1\text{-}HL}$), expressing an *NTC* or *DELE1* sgRNA (*DELE1* KD) were left untreated or treated with 2.5, 5 or 10 μM CCCP, 03, 0.6,1.25 or 2.5 ng/ml oligomycin in the presence or absence of 100 nM bafilomycin A, followed by measurement of mitophagy using flow cytometry. (mean ± s.d., n = 3 independently treated culture wells) h. Immunoblots of ATF4, COX IV, SDHB, MFN2, TIMM23, and PRKN in *DELE1* KD cells with low or medium levels of *PRKN* over expression. β-actin serves as the loading control.

Supplementary Fig. 2

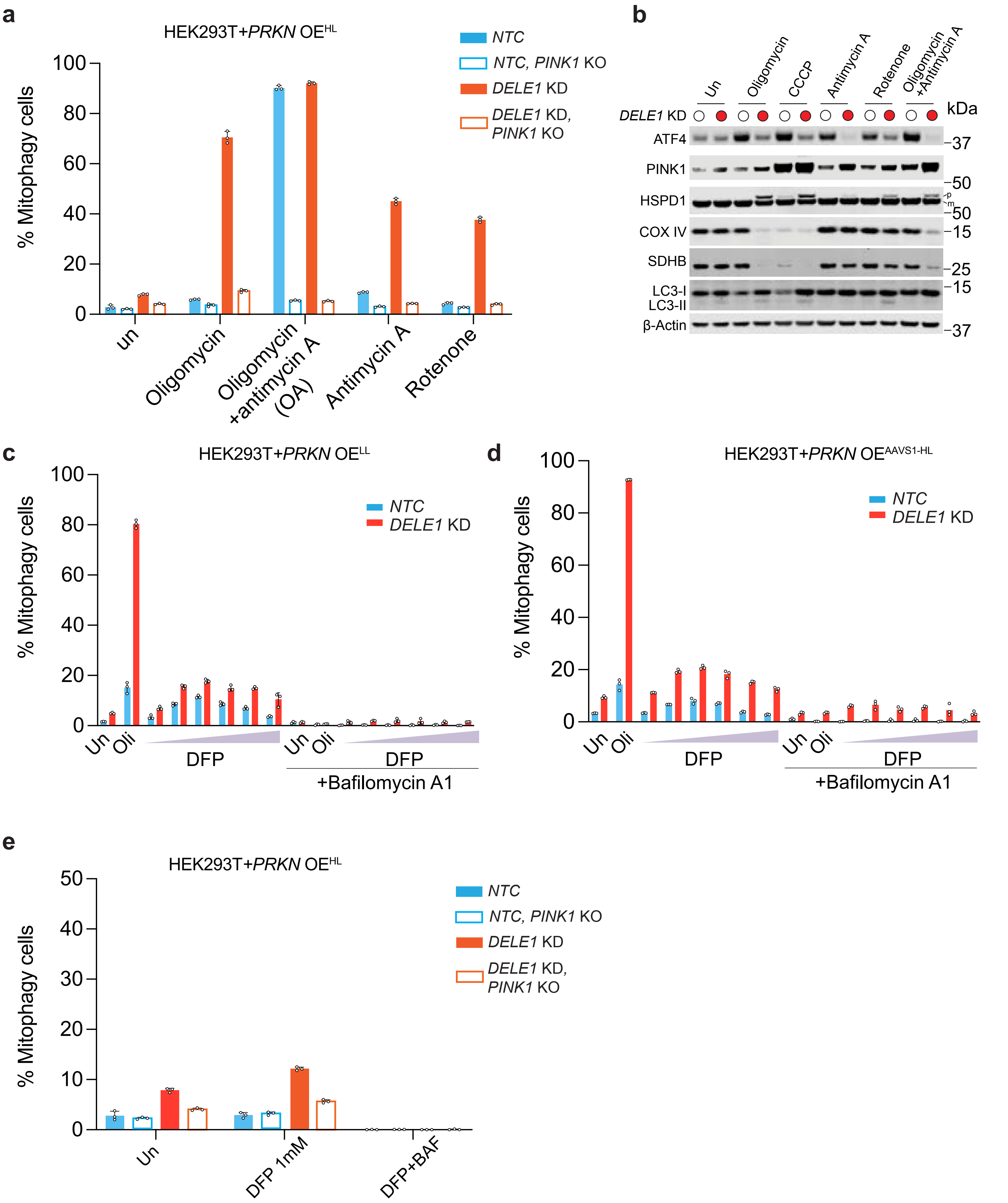

Supplementary Fig.2. Regulation of mitophagy under different types of mitochondrial stress conditions in HEK293T cells. a. HEK293T *PRKN* OE^{HL} wild type (WT) or *PINK1* KO cells with or without *DELE1* KD were treated with 1.25 ng/ml oligomycin, 100 nM rotenone, 100 nM antimycin A, or a combination of oligomycin and antimycin A (OA) for 24 hr, followed by measurement of mitophagy using flow cytometry. (mean ± s.d., n = 3 independently treated culture wells) **b.** Immunoblots of ATF4, PINK1, HSPD1, COX IV, SDHB and LC3 in *PRKN* OE^{ML} cells with *NTC* or *DELE1* KD following treatment with 1.25 ng/mL oligomycin, 10 μM CCCP, 100 nM antimycin A, 100 nM rotenone, or 1.25 ng/mL oligomycin plus 100 nM antimycin A. β-actin serves as the loading control. **c-d.** HEK293T *mtKeima* reporter cells with *PRKN* OE^{LL}, or *PRKN* OE^{HL}, expressing an *NTC* or *DELE1* sgRNA (*DELE1* KD) were left untreated or treated with 0.125, 0.25, 0.5, 1, 2 and 4 mM deferiprone (DFP) in the presence or absence of 100 nM bafilomycin A, followed by measurement of mitophagy using flow cytometry. Oligomycin conditions were included to serve as a positive control. (mean ± s.d., n = 3 independently treated culture wells) **e.** HEK293T *PRKN* OE^{HL} WT or *PINK1* KO cells with or without *DELE1* KD were treated with 1 mM DFP with or without bafilomycin A1 (BAF) for 24 hr, followed by measurement of mitophagy using flow cytometry. (mean ± s.d., n = 3 independently treated culture wells)

Supplementary Fig. 3

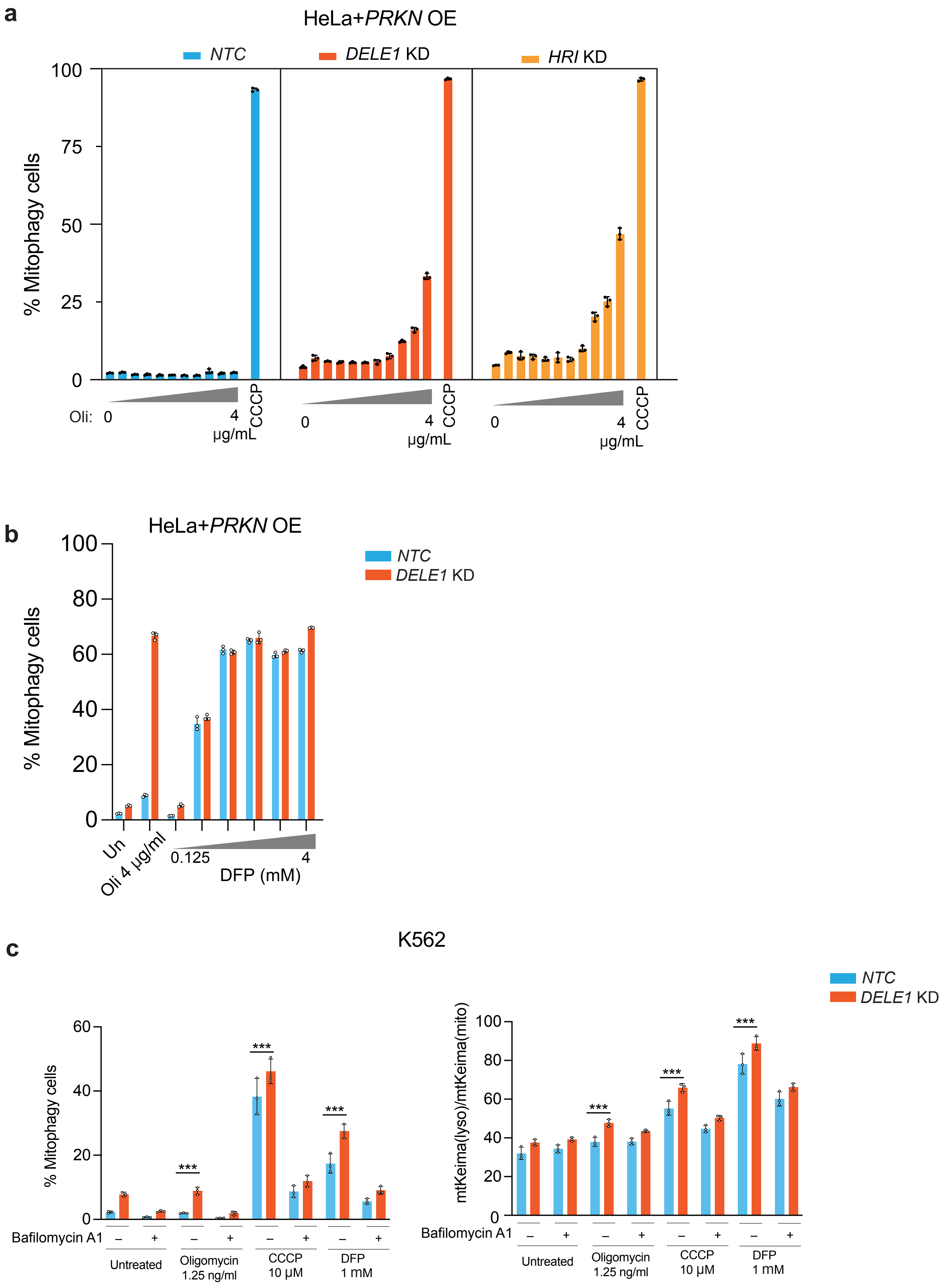

Supplementary Fig.3. DELE1 negatively regulates mitophagy in HeLa and K562 cells. a. HeLa cell line with *PRKN* OE were infected with a *NTC* sgRNA, a *DELE1* sgRNA or an *HRI* sgRNA. These cells were treated with oligomycin (Oli) at following concentrations: 1.25 ng/mL, 2.5 ng/mL, 5 ng/mL, 10 ng/mL, 50 ng/mL, 100 ng/mL, 500 ng/mL, 1 μg/mL, 2 μg/mL, and 4 μg/mL, as well as CCCP at 10 μM for 24 hr, followed by flow cytometry measurement of mitophagy. **b.** HeLa *mtKeima* reporter cells with *PRKN* OE, expressing an *NTC* or *DELE1* sgRNA (*DELE1* KD) were left untreated or treated with 0.125, 0.25, 0.5, 1, 2 and 4 mM DFP, followed by measurement of mitophagy using flow cytometry. Oligomycin conditions were included and served as a positive control. (mean ± s.d., n = 3 independently treated culture wells). **c.** K562 *mtKeima* reporter cells, expressing an *NTC* or *DELE1* sgRNA (*DELE1* KD) were left untreated or treated with1.25 ng/mL oligomycin, 10 μM, or 1 mM DFP with or without bafilomycin A1, followed by measurement of mitophagy using flow cytometry. *left,* quantified by percentage of mitophagy population; *right,* quantified by the ratio of mtKeima (lysosomal) versus mtKeima (mitochondrial). (mean ± s.d., n = 3 independently treated culture wells) Two-way ANOVA test followed by Turkey's multiple comparisons test (two-sided). *** adjusted *p* value < 0.001.

Supplementary Fig. 4

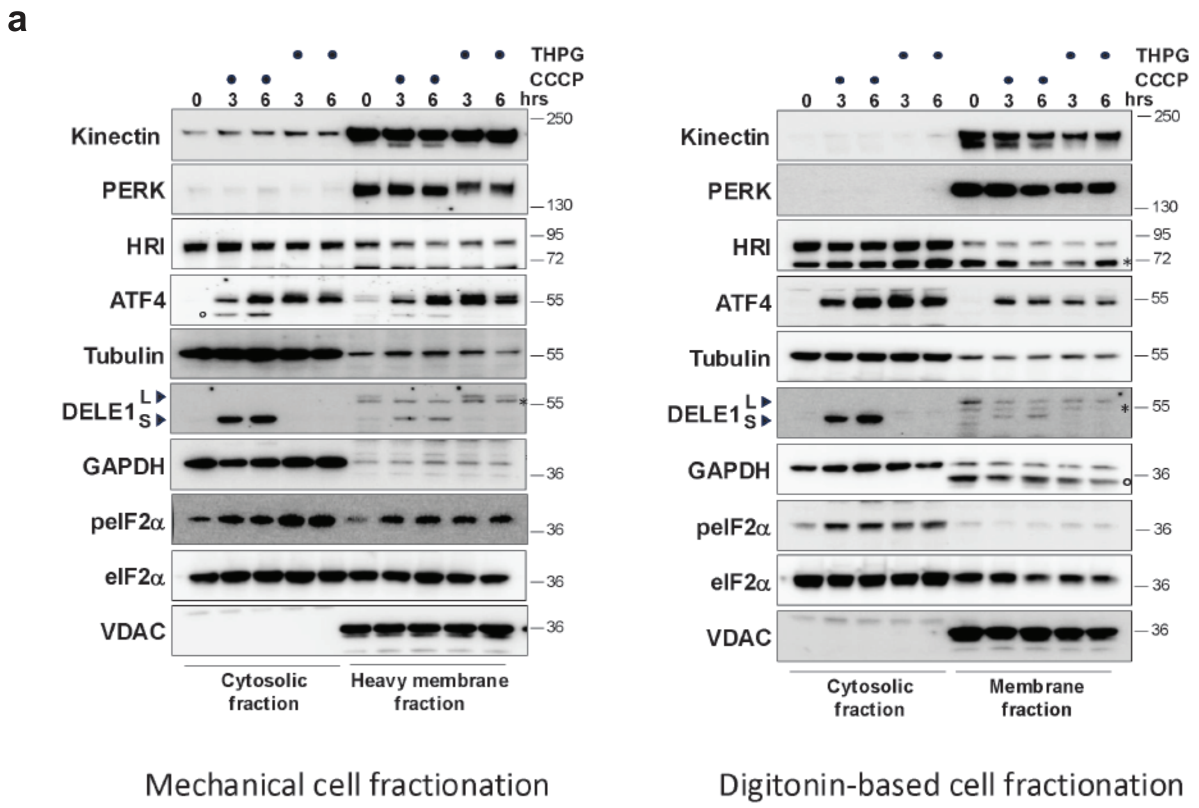

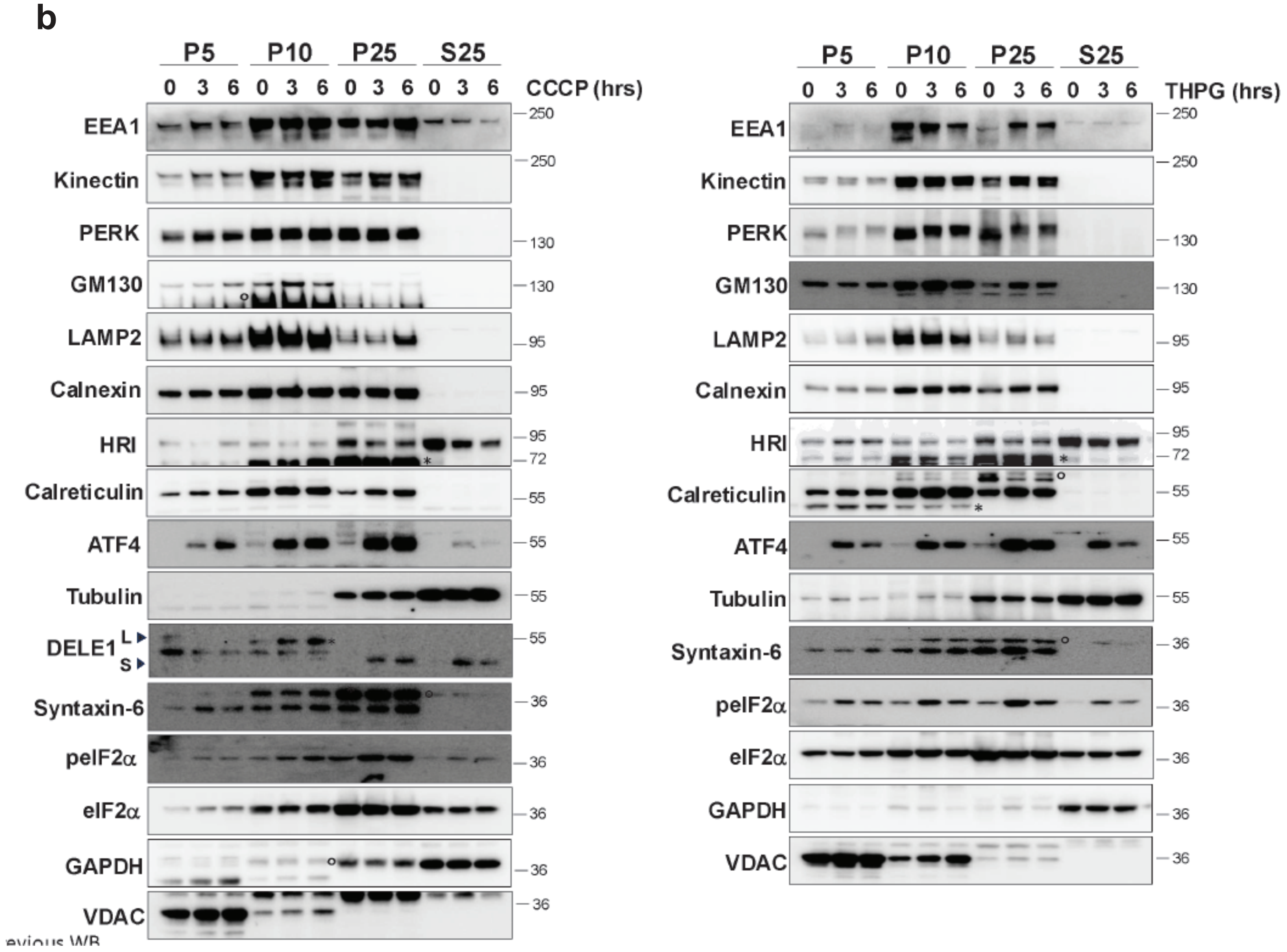

Supplementary Fig. 4 continue

c

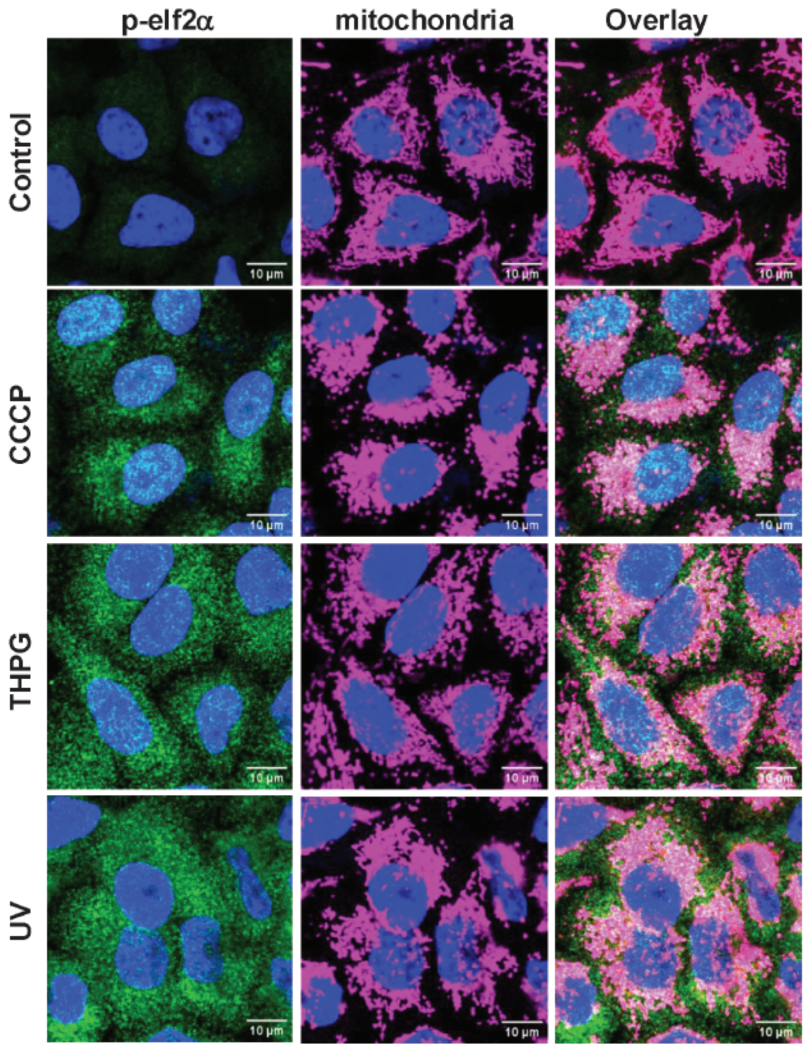

Supplementary Fig.4. Mitochondrial-stress-induced ISR is not associated with a specific mitochondrial localization of phosphorylated eIF2α. a. HeLa cells were either left unstimulated or treated for 3 and 6 hrs with CCCP (10 mM) or thapsigargin (THPG, 500 nM). Next, cytosolic and membrane fractions obtained using mechanical or digitonin-based cell fractionation were analyzed by immunoblotting for the presence of the indicated proteins. ° band from a previous WB. * Non-specific band. **b.** HeLa cells were either left unstimulated or treated with CCCP (10 mM) or thapsigargin (THPG, 500 nM) for the indicated time, then fractionated and samples were analyzed by immunoblotting with antibodies against the indicated proteins. ° band from a previous WB. * Non-specific band. **c.** HeLa cells were either left unstimulated or treated with CCCP (10 mM), thapsigargin (THPG, 500 nM), or UV-C (500 J/m2) for 3 hrs. p-eIF2α was then analyzed by immunofluorescence. Mitochondria were stained with an antibody raised against Hsp60. Scale bar: 10 µm.

Supplementary Fig. 5

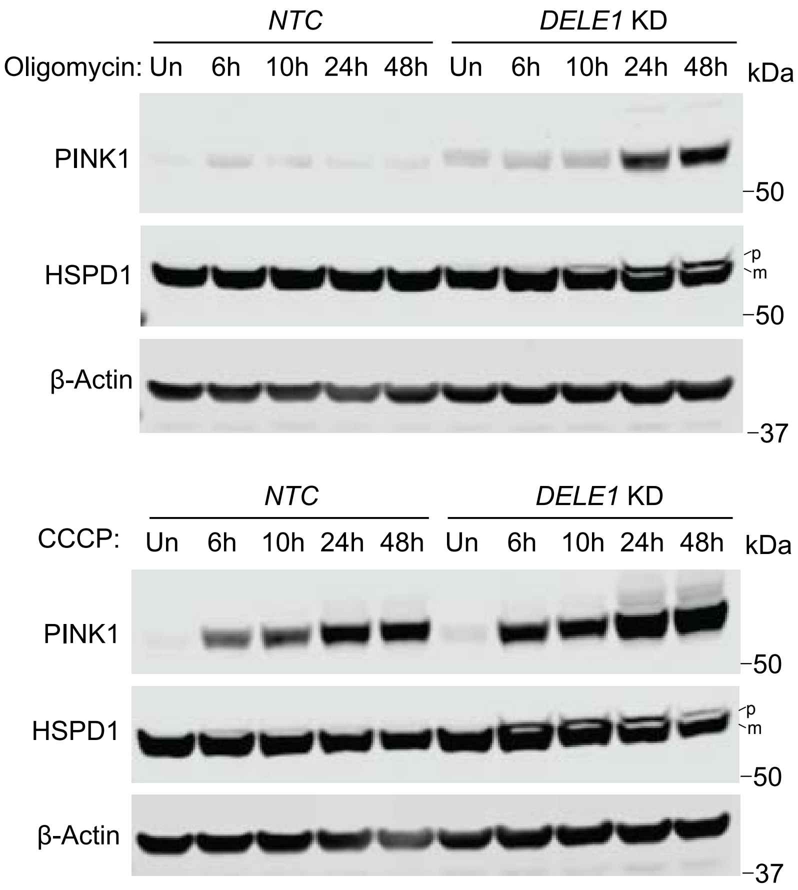

Supplementary Fig.5. PINK1 accumulation occurs in *DELE1* KD cells at a slower rate following oligomycin treatment compared to CCCP. WT and DELE1 KD cells were treated with oligomycin or CCCP for indicated time for immunoblotting analysis of PINK1 and HSPD1.

Supplementary Fig. 6

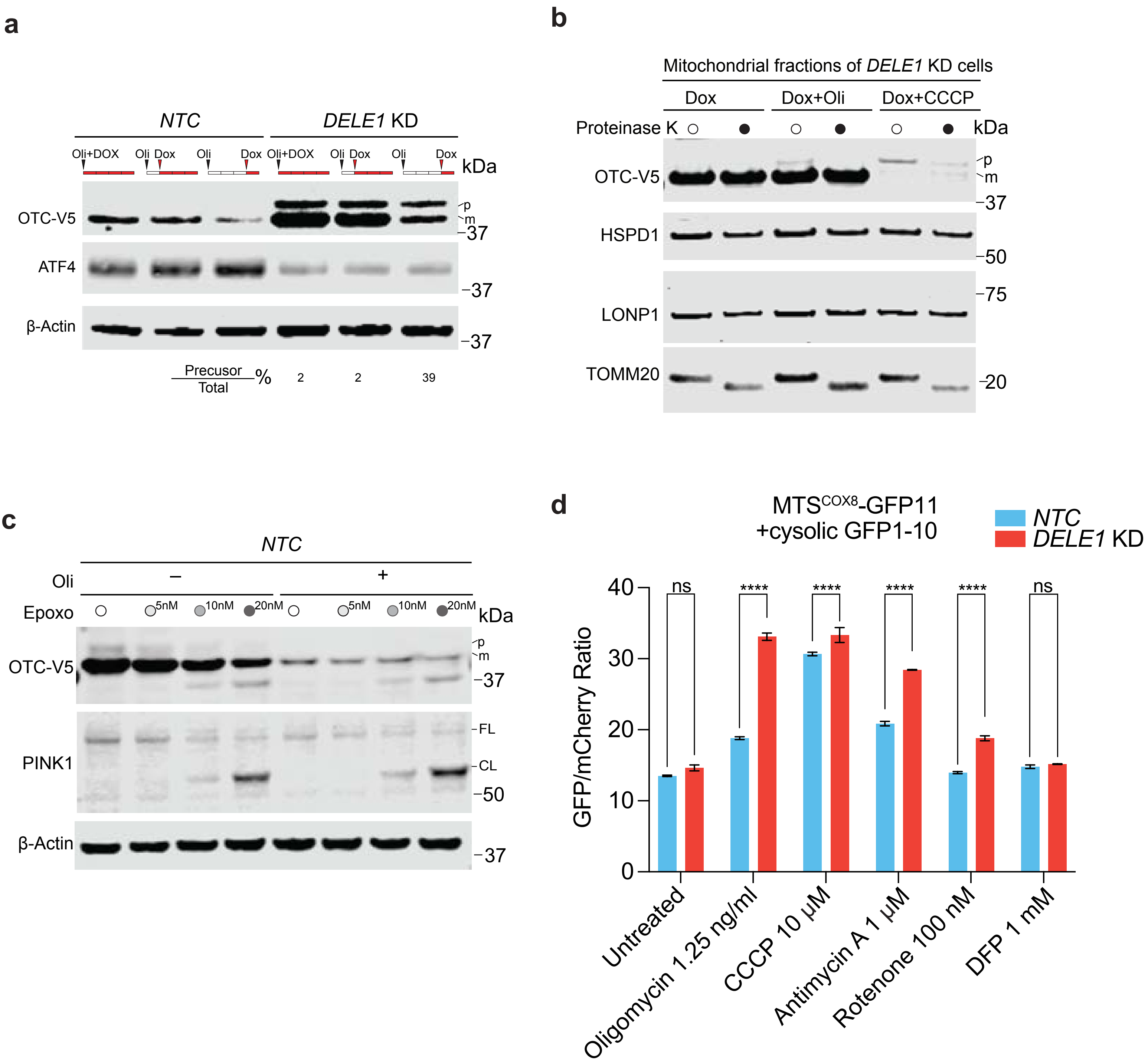

Supplementary Fig.6 DELE1 preserves mitochondrial protein import under different mitochondrial stress conditions. a. Immunoblots of OTC-V5 and ATF4 following co-induction of OTC-V5 with oligomycin, or after 6 h or 18 h of oligomycin pretreatment. **b.** Immunoblots of OTC-V5, HSPD1, LONP1 and TOMM20 from mitochondrial fractions of *DELE1* KD cells, following treatment with 1.25 ng/mL oligomycin or 10 μM CCCP. Mitochondrial fractions were divided into two equal portions, one left untreated and the other was digested with 20 ug/mL Proteinase K for 15 min on ice. **c.** Immunoblots of OTC-V5 and PINK1 following cotreatment of oligomycin with epoxomicin at 5, 10 and 20 nM in WT cells. **d.** Measurement of the import of MTS^{COX8}-GFP11 in GFP1-10 (cytosolic) cells with an *NTC* or *DELE1* sgRNA (*DELE1* KD) following treatment with 1.25 ng/mL oligomycin, 10 μM CCCP, 1 μM antimycin A, 100 nM rotenone, or 1mM DFP. MTS^{COX8}-GFP11 was induced simultaneously during drug treatment by adding 500 ng/mL doxycycline. (mean ± s.d., n = 3 independently treated culture wells) Two-way ANOVA test followed by Turkey's multiple comparisons test. ns, not significant, **** adjusted *p* value < 0.001.

Supplementary Fig. 7

a

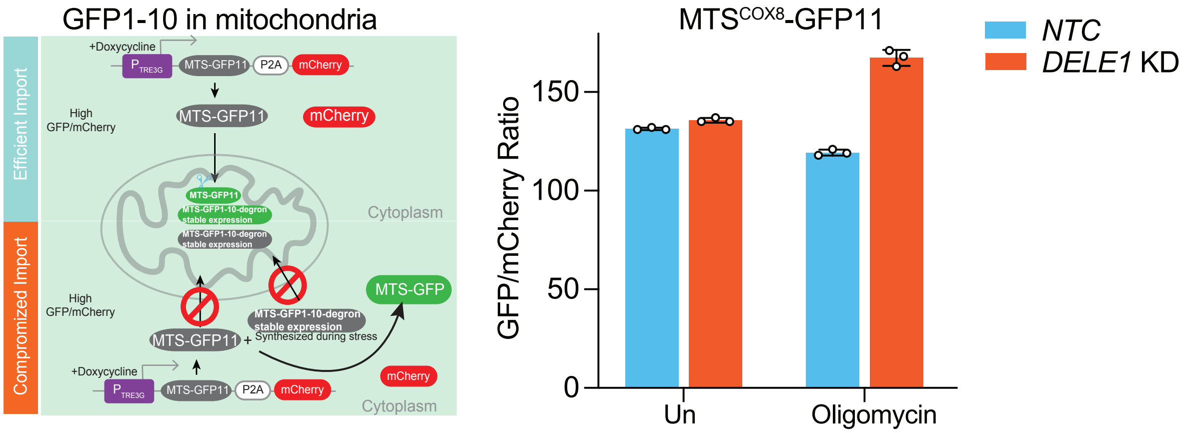

b

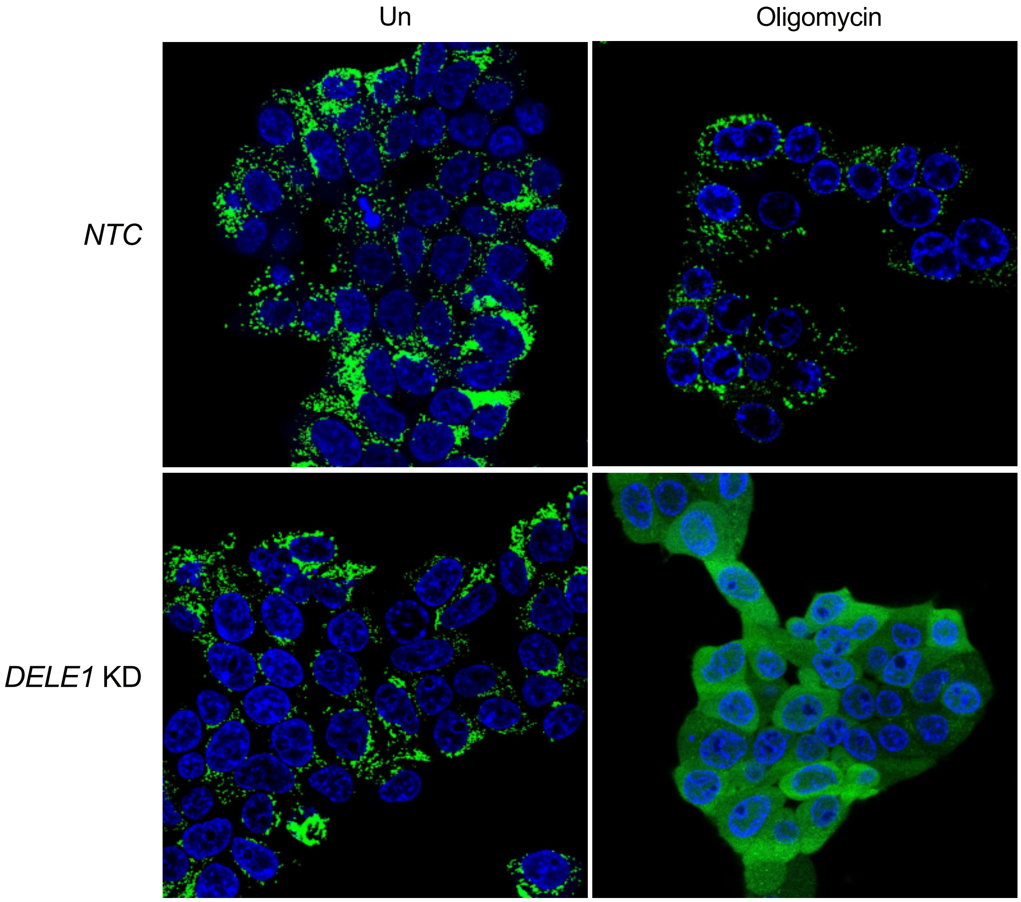

Supplementary Fig.7 The degron is required for reliable assessment of mitochondrial import using the Split-GFP reporter. a. *left,* Schematic illustration for monitoring mitochondrial protein import using split-GFP (inducible MTS-GFP11+ constitutive MTS-GFP1-10 without a degron) reporter via flow cytometry. *right,* Flow cytometry data indicate a slight increase of GFP/mcherry ratio in DELE1 KD cells following oligomycin treatment. b. The reporter cell lines were treated with doxycycline to induce MTS-GFP11 concurrently with oligomycin and imaged via confocal microscope.

Supplementary Fig. 8

a

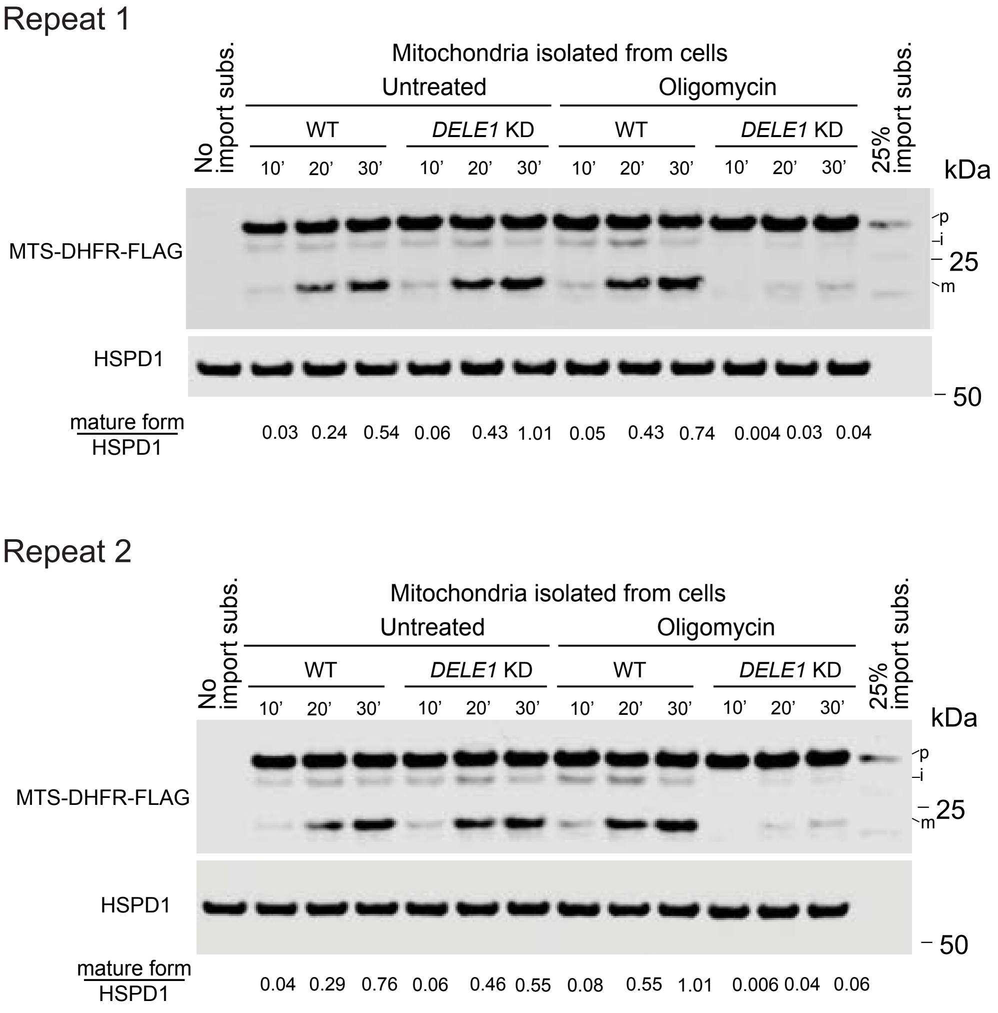

b

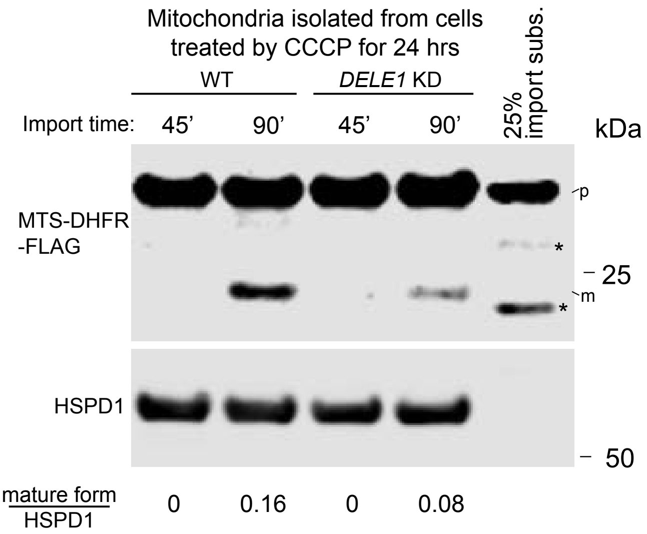

Supplementary Fig.8 DELE1 is not essential for baseline protein import but maintains minimal protein import following CCCP treatment. a. Cell free mitochondrial protein import assay using mitochondrial isolated from WT and *DELE1* KD cells, either left untreated or treated with oligomycin for 24 h. The mitochondria were incubated with MTS-DHFR-flag for the indicated time. **b.** Cell free mitochondrial protein import assay using mitochondrial isolated from WT and *DELE1* KD cells treated with 10μM CCCP for 6 h followed by immunoblots of the import substrate and HSPD1. *p* precursor; i, intermediate; *m*, mature; * non-specific band.

Supplementary Fig. 9

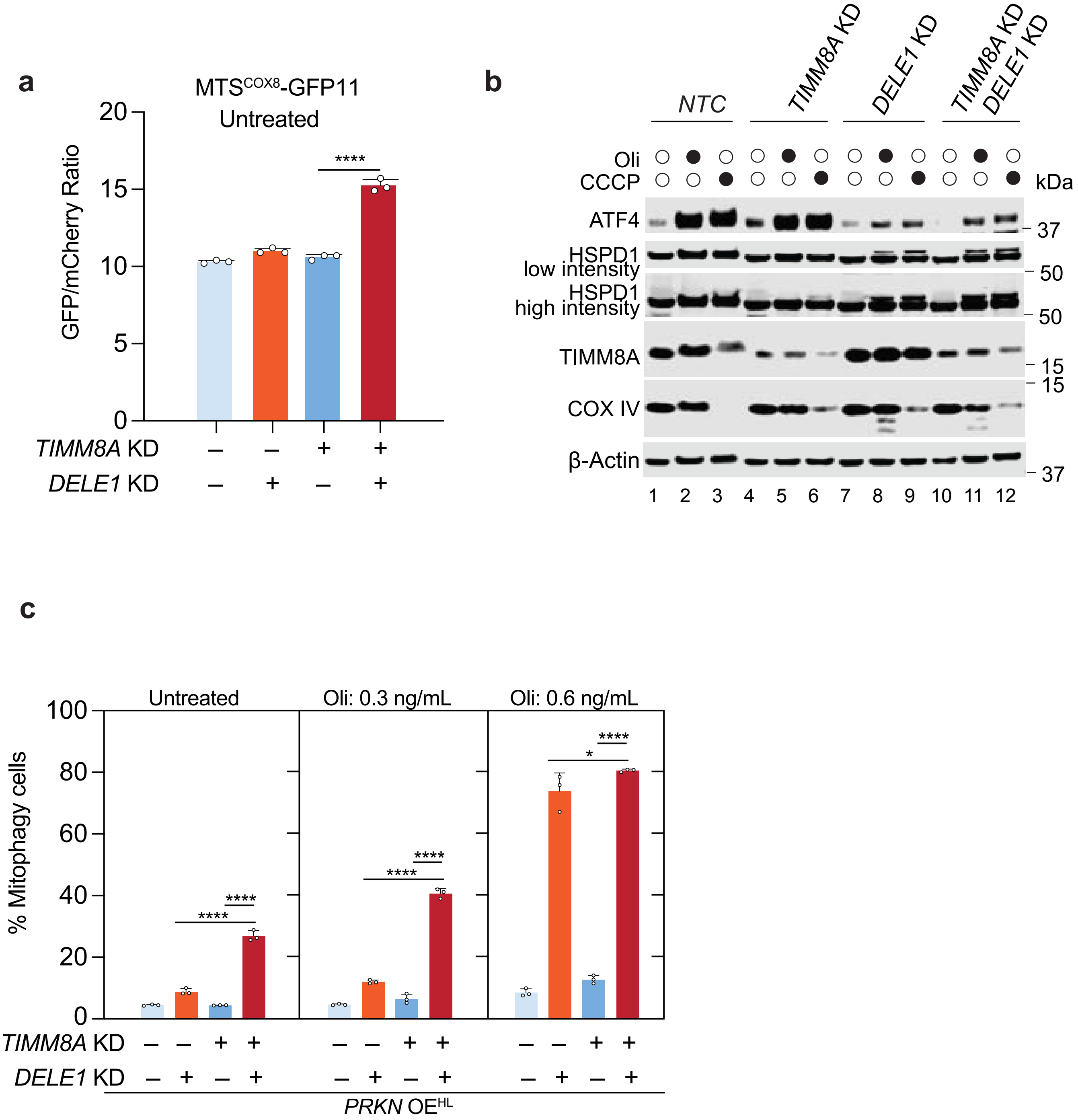

Supplementary Fig.9 DELE1 maintains minimal protein import and suppress mitophagy following *TIMM8A* KD. a. Measurement of the import of MTS^{COX8}-GFP11 in HEK293T cells with an *NTC sgRNA*, a *DELE1* sgRNA, a *TIMM8A* sgRNA or *TIMM8A and DELE1* double sgRNAs. No additional mitochondrial stress was used in this assay. **b.** Immunoblots of ATF4, HSPD1, TIMM8A and COX IV in cells with *NTC*, *TIMM8A* KD, *DELE1* KD or *TIMM8A, DELE1* double KD cells following treatment with 10 μM CCCP or 0.6 ng/mL oligomycin. **c.** Measurement of mitophagy in *PRKN* OE^{HL} cells with a *NTC sgRNA*, a *DELE1* sgRNA, a *TIMM8A* sgRNA or *TIMM8A and DELE1* double sgRNAs, following no treatment, 0.3 ng/mL or 0.6 ng/mL oligomycin. (mean ± s.d., n = 3 independently treated culture wells) Two-way ANOVA test followed by Turkey's multiple comparisons test (two-sided). **** adjusted p value < 0.0001; * adjusted p value = 0.0158

Supplementary Fig. 10

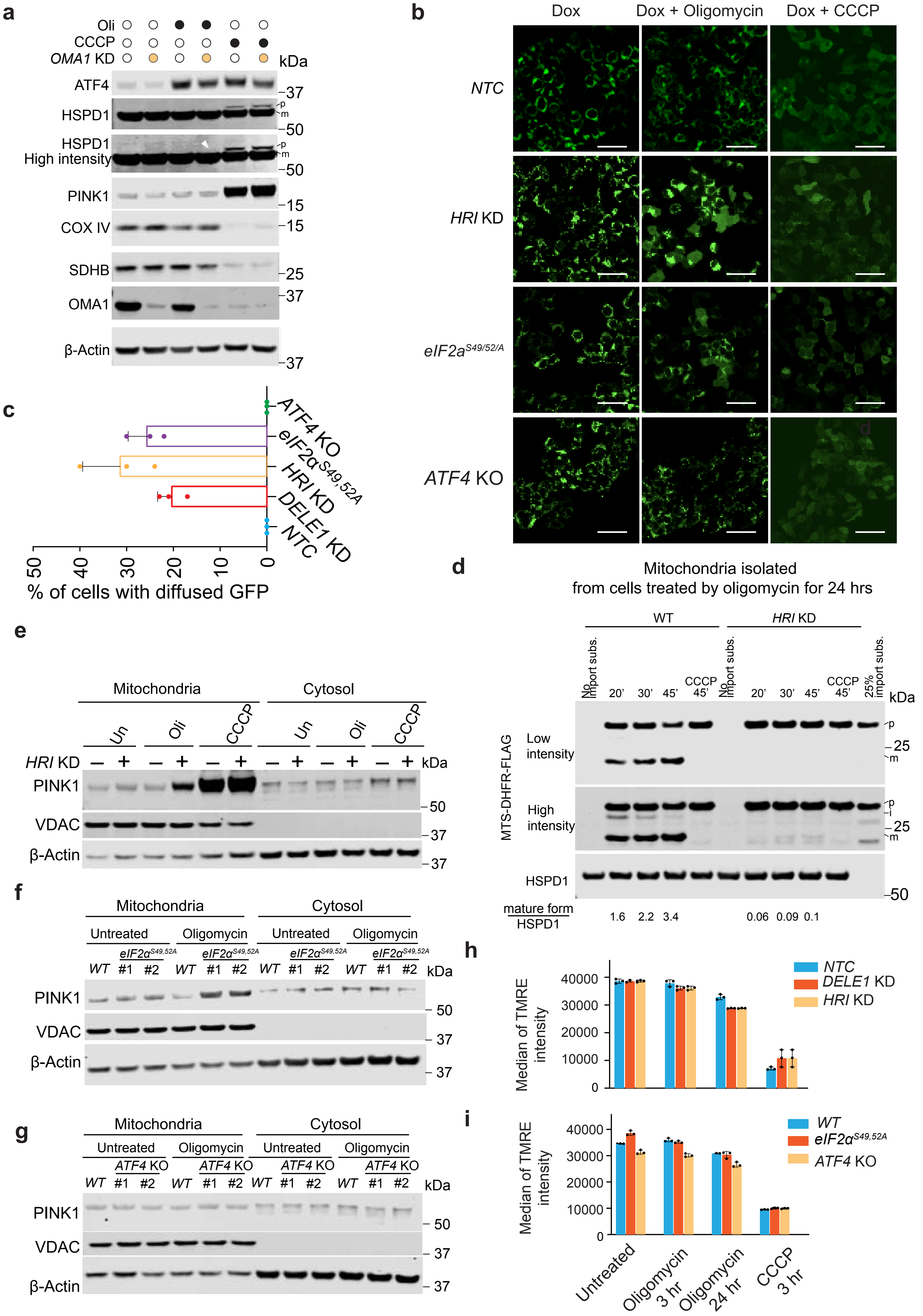

Supplementary Fig.10. DELE1-ISR pathway preserves mitochondrial protein import in an ATF4-independent manner. a. Immunoblots of ATF4, HSPD1, PINK1, COX IV, OMA1 and β-Actin. *NTC* and *OMA1* KD cells were left untreated or treated with 10 μM CCCP or 1.25 ng/mL oligomycin for 24 h. β-Actin serves as loading control. White arrowhead: a faint band of precursor form of HSPD1 is visible in *OMA1* KD cells following oligomycin treatment. **b.** Inducible MTS^{COX8}-*YFP* cells with *NTC* sgRNA, *DELE1* sgRNA, *HRI* sgRNA, *DELE1* sgRNA, $eIF2\alpha^{S49/52/A}$ and *ATF4* KO were treated with 500 ng/mL doxycycline concurrently with 1.25 ng/mL oligomycin or 10 μM CCCP for overnight before imaging. Scale bar: 100 μm. **c.** Quantification of MTS^{COX8}-YFP import under oligomycin condition. **d.** Cell free mitochondrial protein import assay using mitochondrial isolated from WT and *HRI* KD cells treated with oligmycin for 24 h followed by immunoblots of the import substrate and HSPD1. *p* precursor; i, intermediate; *m*, mature; * non-specific band. **e-g.** Immunoblots of PINK1, VDAC1 and β-actin from mitochondrial and cytosolic fractions prepared from *HRI* KD **(e)**, two clonal $eIF2\alpha^{S49/52/A}$ cells **(f)**, or *ATF4* KO cells **(g)** following treatment with 1.25 ng/mL oligomycin or 10 μM CCCP treatment for 24 h. **h. and i.** HEK293T cells with an *NTC* sgRNA, an *HRI* sgRNA, a *DELE1* sgRNA, wild type (*WT*), $eIF2\alpha^{S49/52/A}$ and *ATF4* KO were treated with 10 μM CCCP for 3 hr, as a positive control for mitochondrial depolarization, and 1.25 ng/ml oligomycin for 3 or 24 hr, followed by 100 nM TMRE staining and flow cytometry analysis. (mean ± s.d., n = 3 independently treated culture wells)

Supplementary Fig. 11

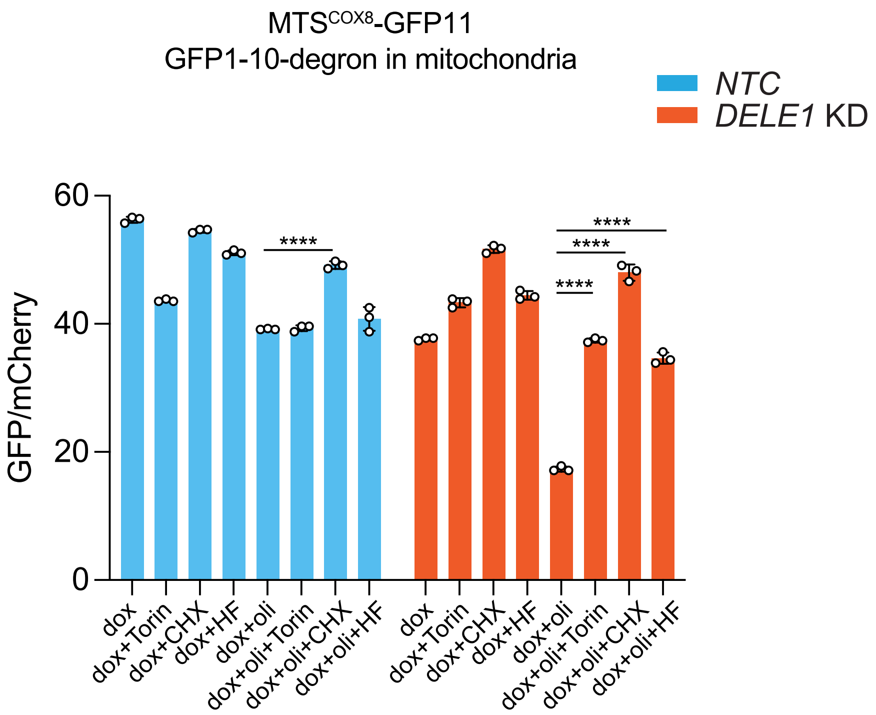

Supplementary Fig.11. Protein attenuation alleviates impairment of mitochondrial protein import in *DELE1* KD cells following oligomycin treatment. MTS-GFP1-11 was induced in cells expressing MTS-GFP1-10-degron and treated concurrently with oligomycin, either alone or in combination with protein synthesis inhibitors cycloheximide (CHX) or Torin1, or with the integrated stress response (ISR) inhibitor Halofuginone (HF). (mean ± s.d., n = 3 independently treated culture wells) Two-way ANOVA test followed by Turkey's multiple comparisons test. **** adjusted p value < 0.0001.

Supplementary Fig. 12

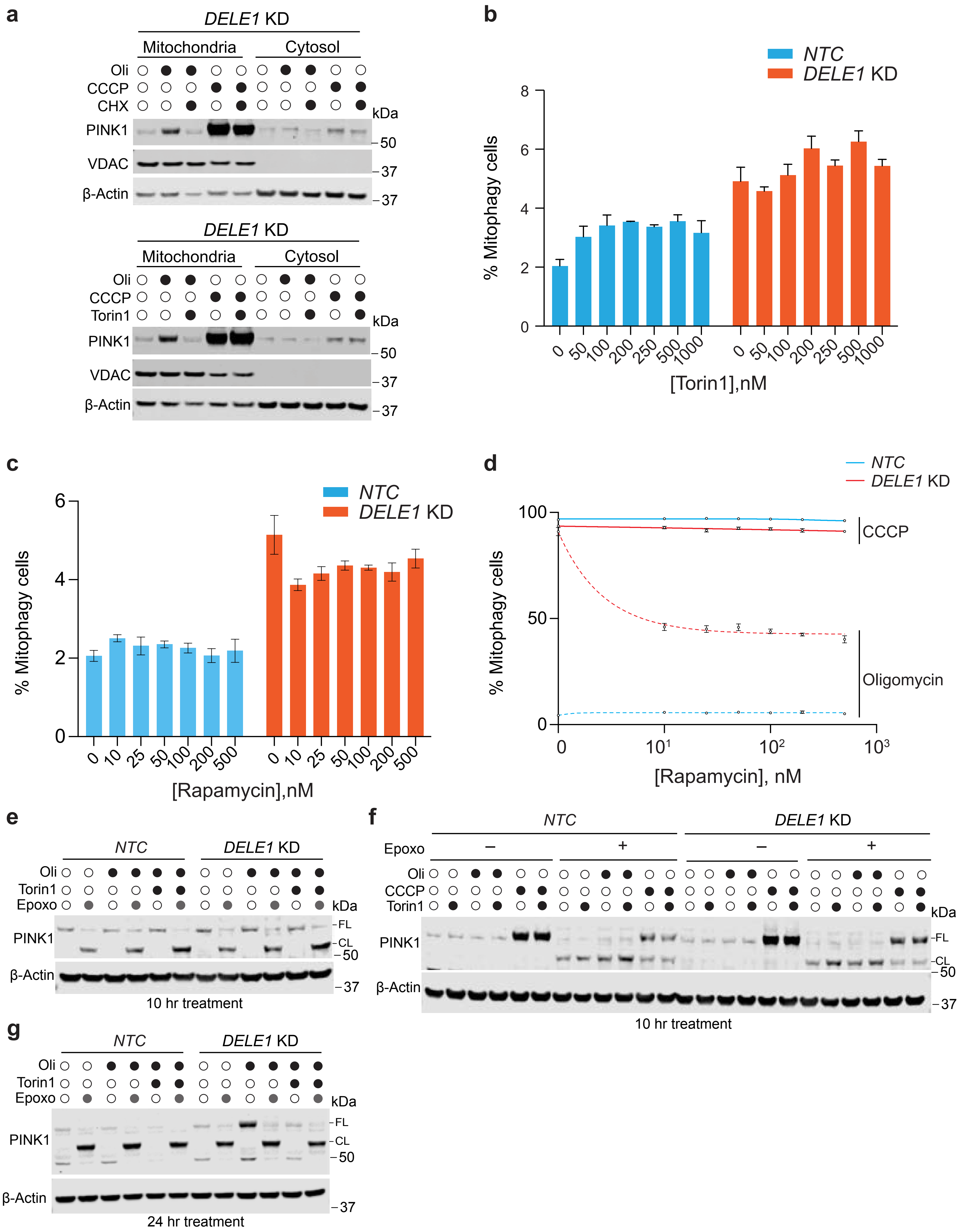

Supplementary Fig.12. Torin1 and rapamycin slightly increase mitophagy but suppress oligomycin-induced mitophagy in *DELE1* KD cells. a. Immunoblots of PINK1, VDAC1 and β-actin from mitochondrial and cytosolic fractions prepared from *DELE1* KD cells. Top panel: cells were treated with 1.25 ng/mL oligomycin or 10 μM CCCP in the presence or absence of 100 ng/mL cycloheximide (CHX). Bottom panel: cells were treated with 1.25 ng/mL oligomycin or 10 μM CCCP in the presence or absence of 250 nM Torin1. **b.** *NTC* and *DELE1* KD cells were treated with Torin1 at concentrations ranging from 50 nM to 1000 nM, followed by flow cytometry to measure mitophagy. (mean ± s.d., n = 3 independently treated culture wells) **c.** *NTC* and *DELE1* KD cells were treated with rapamycin at concentrations ranging from 10 nM to 500 nM, followed by flow cytometry to measure mitophagy. (mean ± s.d., n = 3 independently treated culture wells) **d.** *NTC* and *DELE1* KD cells with *PRKN* OE^{HL} are treated with 10 μM CCCP or 1.25 ng/mL oligomycin in the presence of rapamycin at 7 different concentrations (0, 10, 25, 50, 100, 20 and 500 nM) for 24 h followed by flow cytometry to measure mitophagy. Rapamycin concentrations were converted to their base-10 logarithmic values. A nonlinear regression analysis using a log(inhibitor) vs. response model with a variable slope (four parameters) was performed to generate the plot. (mean ± s.d., n = 3 independently treated culture wells) **e-f.** Immunoblots of PINK1 in cells with *NTC* or *DELE1* KD following treatment with different combinations of drugs for about 10 h as indicated. FL: full length PINK1; CL: cleaved PINK1. Oligomycin (Oli): 2.5 ng/mL, Torin1: 250 nM, CCCP: 10 μM, epoxomicin: 300 nM. **g.** Immunoblots of PINK1 in cells with *NTC* or *DELE1* KD following treatment with different combinations of drugs for about 24 h as indicated. FL: full length PINK1; CL: cleaved PINK1. Oligomycin (Oli): 1.25 ng/mL, Torin1: 250 nM, epoxomicin: 50 nM. FL: full length PINK1 ; CL: cleaved PINK1.

Supplementary Fig.13

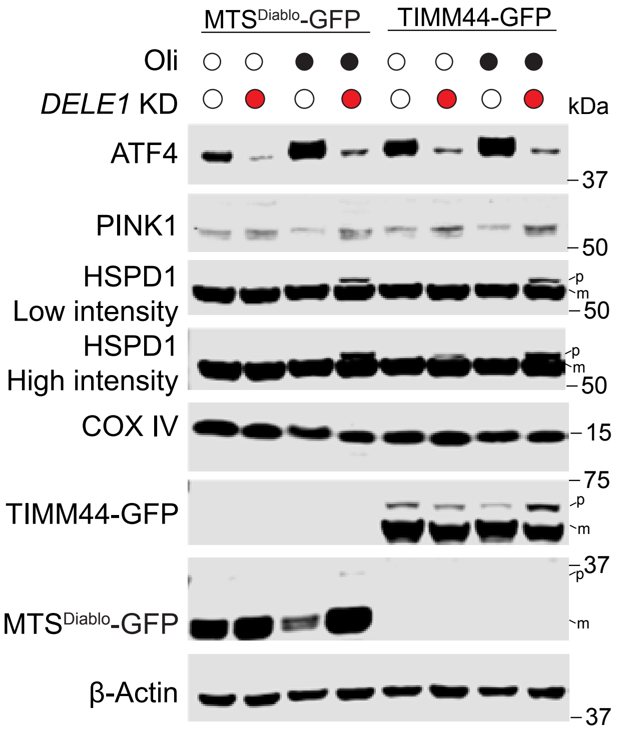

Supplementary Fig.13. Overexpression of mitochondrial protein TIMM44 induces mild mitochondrial import stress. Immunoblots of ATF4, PINK1, HSPD1, COX IV, TIMM44-GFP, and MTS^{Diablo}-GFP in cells with overexpression of MTS^{Diablo}-GFP or TIMM44-GFP with or without *DELE1* KD. p: precursor, m: mature.

Supplementary Fig. 14

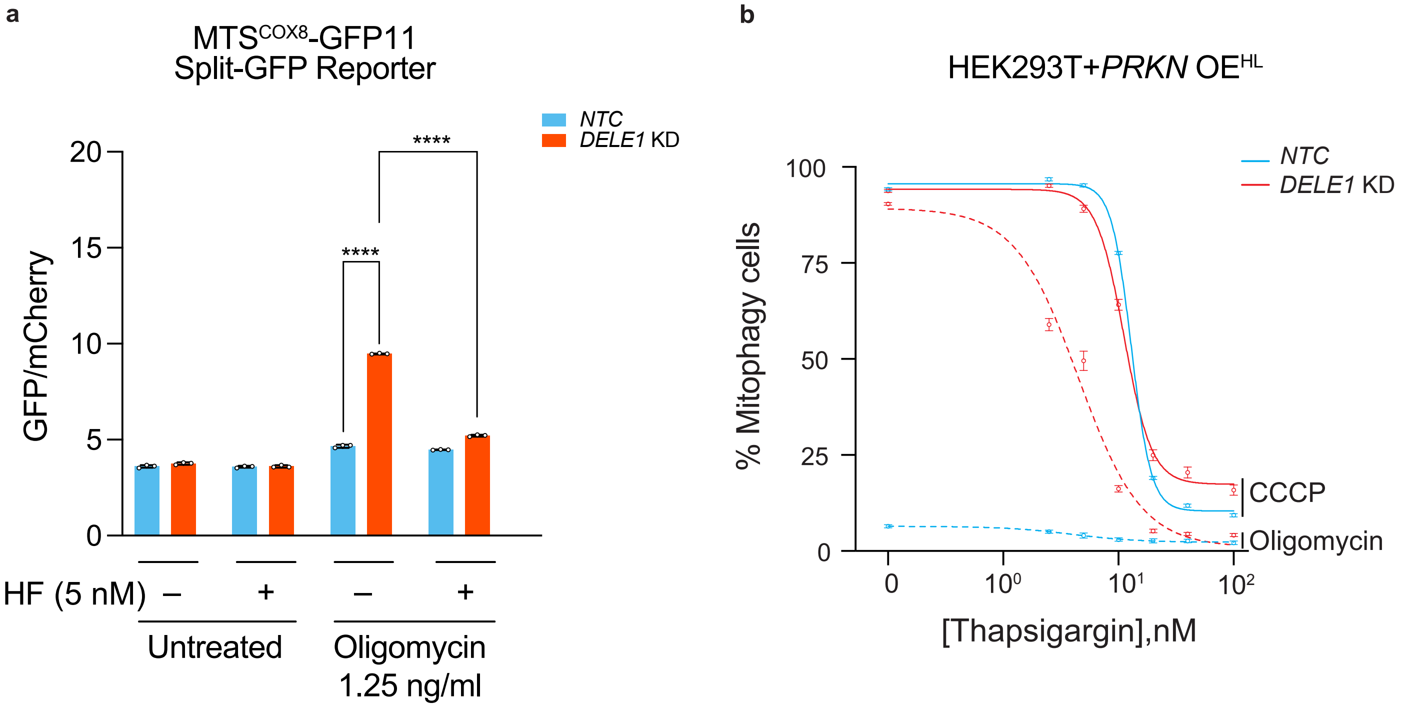

Supplementary Fig.14. Activation of alternative ISR pathways rescue mitochondrial import and suppress mitophagy in DELE1-ISR-deficient cells. a. Measurement of the import of MTS^{COX8}-GFP11 in HEK293T cells with an *NTC* or *DELE1* sgRNA (*DELE1* KD) following treatment with 1.25 ng/mL oligomycin in the presence or absence of 5 nM halofuginone (HF). MTS^{COX8}-GFP11 was induced during mitochondrial stress. (mean ± s.d., n = 3 independently treated culture wells) Two-way ANOVA test followed by Turkey's multiple comparisons test. **** adjusted *p* value < 0.0001. **b.** *NTC* and *DELE1* KD cells with *PRKN* OE^{HL} are treated with 10 μM CCCP or 1.25 ng/mL oligomycin in the presence of thapsigargin at 7 different concentrations (0, 2.5, 5, 10, 20, 40 and 100 nM) for 24 h followed by flow cytometry to measure mitophagy. Thapsigargin concentrations were converted to their base-10 logarithmic values. A nonlinear regression analysis using a log(inhibitor) vs. response model with a variable slope (four parameters) was performed to generate the plot. (mean ± s.d., n = 3 independently treated culture wells)

Supplementary Fig. 15

a

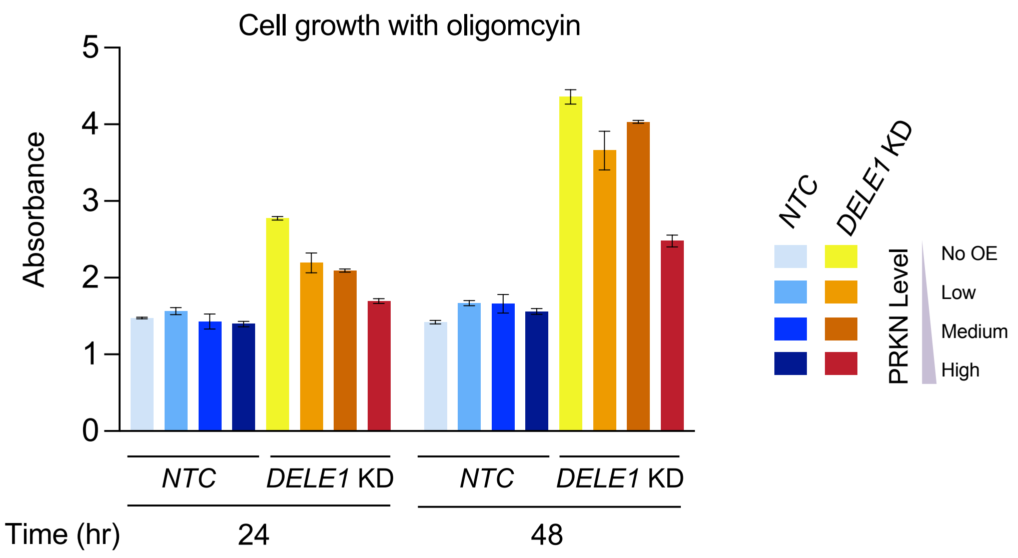

b

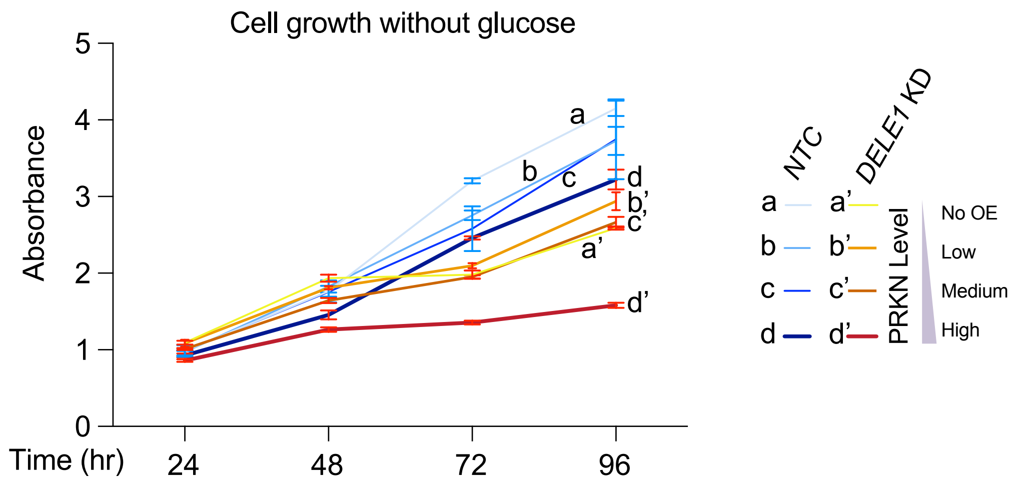

Supplementary Fig.15. Quantification of the crystal violet assays in Fig. 6. a. Crystal violet staining of cells with different expression levels of PRKN with or without *DELE1* KD following treatment with 1.25 ng/mL for 24 and 48 h. (mean ± s.d., n = 3 independently treated culture wells) **b.** Crystal violet staining of cells with different expression levels of PRKN cultured without glucose in the medium for 24, 48, 72 and 96 h respectively. (mean ± s.d., n = 3 independently treated culture wells)

Supplementary Fig. 16

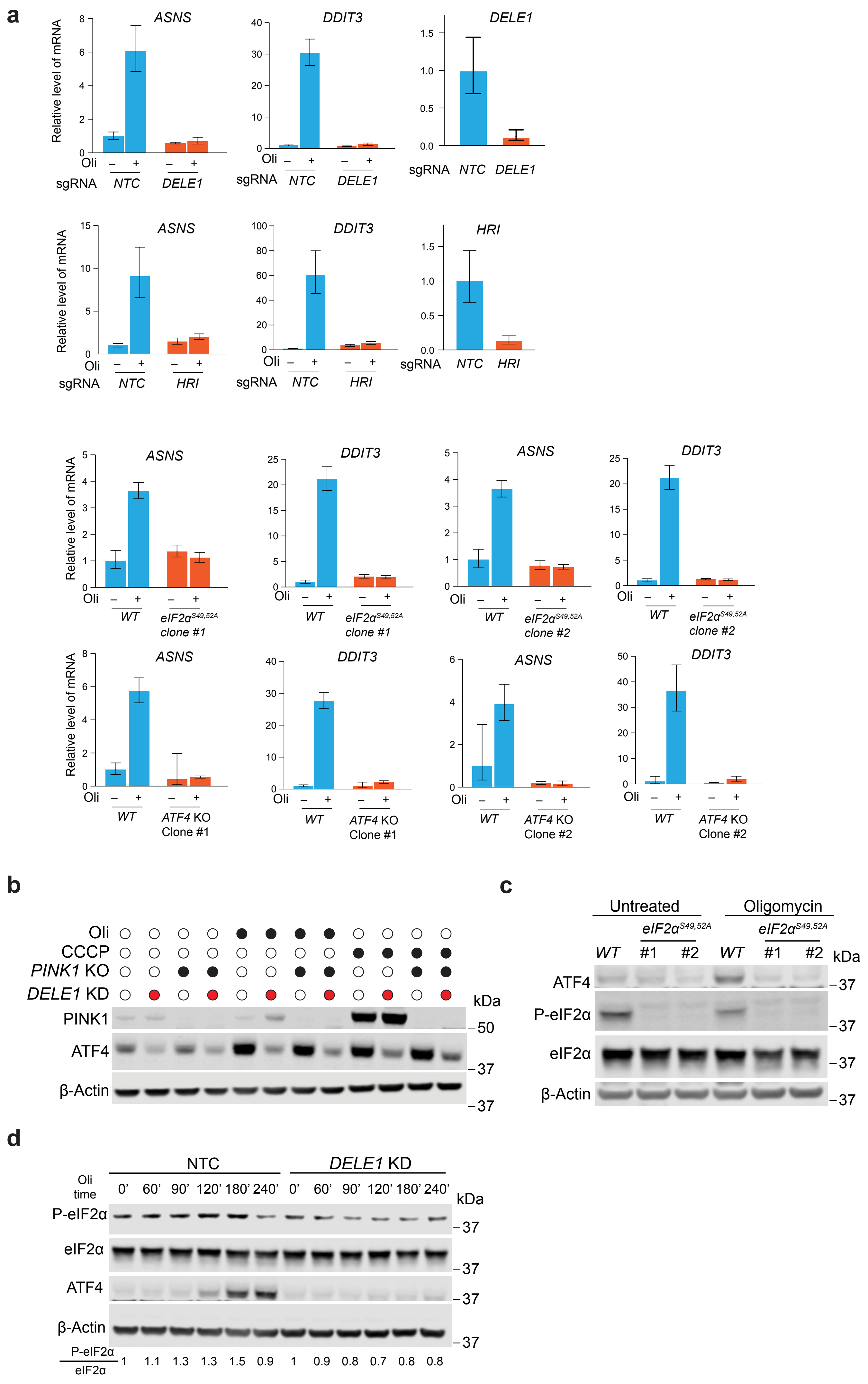

Supplementary Fig.16. Validation of loss of the ISR activation in different cell lines. a. *NTC, HRI* KD and *DELE1* KD, wild type (WT), *eIF2*$\alpha^{S49/52/A}$ and *ATF4* KO cells were left untreated or treated with 1.25 ng/mL oligomycin for 6 h before harvesting for RNA extraction, cDNA synthesis and qPCR. ISR target genes *ASNS* and *DDIT3* were used to evaluate the activation of the ISR. For *DELE1* and *HRI* KD, qPCR of *DELE1* and *HRI* were also performed. (n = 3) **b.** Immunoblots of ATF4, PINK1and β-actin. *WT* and *PINK1* KO cells with or without *DELE1* KD were treated with 1.25 ng/mL oligomycin or 10 μM CCCP for 24 h. β-Actin serves as the loading control. **c.** Immunoblots of ATF4, phosphorylated eIF2α (P- eIF2α), total eIF2α and β-actin. WT and two clonal *eIF2*$\alpha^{S49/52/A}$ cells were treated with 1.25 ng/mL oligomycin for 24 h. β-Actin serves as the loading control. **d.** Immunoblots of ATF4, phosphorylated eIF2α, total eIF2α and β-actin. *NTC* and *DELE1* KD cells were treated with 1.25 ng/mL oligomycin for 0, 60, 90, 120, 180 and 240 min. Numbers at the bottom indicate p-eIF2α/total eIF2α ratios, normalized to untreated NTC samples.

Supplementary Fig. 17

a

Oligomycin 1.25 ng/mL

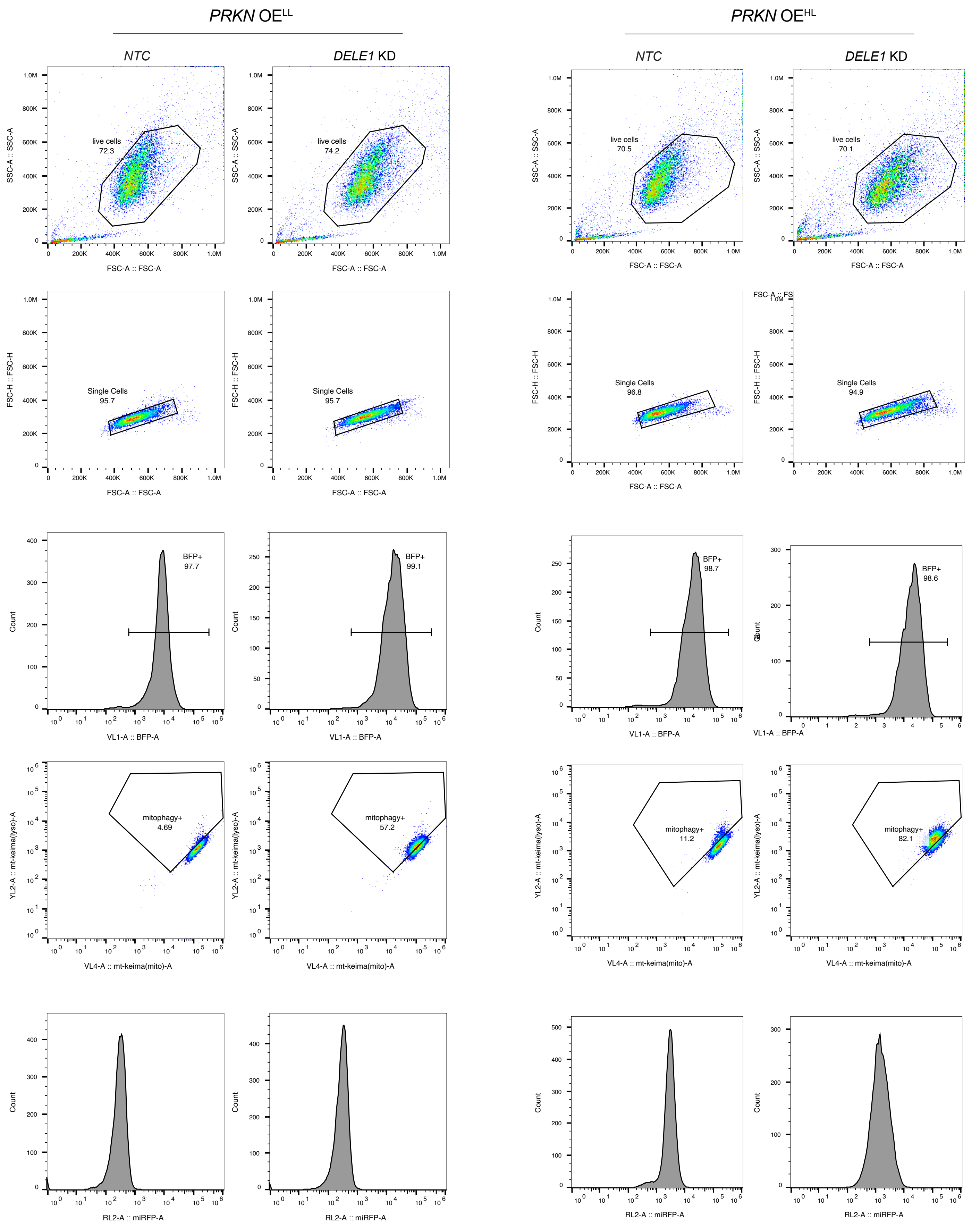

Supplementary Fig. 17

b

Oligomycin 1.25 ng/mL

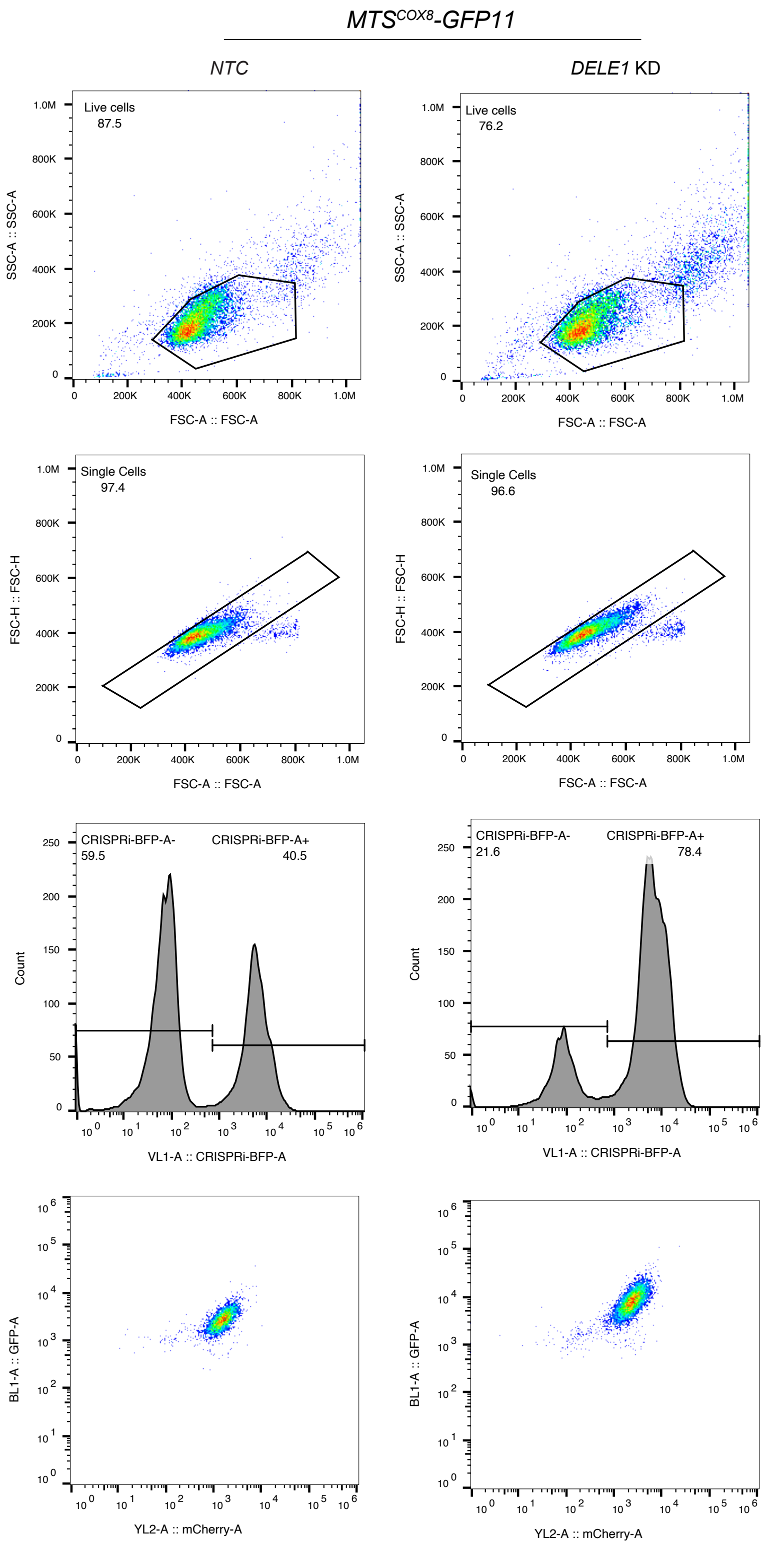

CRISPRi-BFP-A+ : Median : CMV-GFP-A: 2554	CRISPRi-BFP-A+ : Median : CMV-GFP-A: 7366
CRISPRi-BFP-A+ : Median : Normalized GFP: 17.5	CRISPRi-BFP-A+ : Median : Normalized GFP: 32.8
CRISPRi-BFP-A+ : Median : ATF4-mCherry-A: 1501	CRISPRi-BFP-A+ : Median : ATF4-mCherry-A: 2299

Supplementary Fig.17. Examples of gating strategies for mitophagy measurement and split-GFP using flow cytometry. From the live cells, single cells are gated based on forward scatter area versus forward scatter height. From the single cells, BFP positive cells are these with sgRNA. The high mitophagy population is first gated in NTC cells under untreated condition, and the same gate is applied throughout the experiments. mtKeima is measured by two channels: YL2 (mtKeima(lyso)): Ex 637, Em 620 ± 7.5; VL4 (mtKeima(mito)): Ex 405, Em 660 ± 10; miRFP is measured using RL2 channel Ex 637, Em 720 ± 15. BFP signal indicates sgRNA positivity. **a.** Examples of mitophagy measurements in non-targeting (*NTC*) sgRNA positive or *DELE1* sgRNA positive cells with a low level of PRKN or with a high level of PRKN following oligomycin treatment. **b.** Mitochondrial protein import was calculated as a ratio between GFP vs mCherry intensities. Examples here include non-targeting (*NTC*) sgRNA positive or *DELE1* sgRNA positive cells following oligomycin treatment.
