# Supplementary material for: The integrated stress response suppresses PINK1-dependent mitophagy by preserving mitochondrial import efficiency
Source: Nat Commun. 2026 Apr 9;17:4838. doi: 10.1038/s41467-026-71630-6 (PMC13223273; doi:10.1038/s41467-026-71630-6)
Supplement: Supplementary file 2 — Reporting Summary [file 41467_2026_71630_MOESM2_ESM.pdf]

nature portfolio

Corresponding author(s): Xiaoyan Guo
Last updated by author(s): Mar 4, 2026

Reporting Summary

Nature Portfolio wishes to improve the reproducibility of the work that we publish. This form provides structure for consistency and transparency in reporting. For further information on Nature Portfolio policies, see our Editorial Policies and the Editorial Policy Checklist.

Statistics

For all statistical analyses, confirm that the following items are present in the figure legend, table legend, main text, or Methods section.

n/a	Confirmed	
☐	☒	The exact sample size (*n*) for each experimental group/condition, given as a discrete number and unit of measurement
☐	☒	A statement on whether measurements were taken from distinct samples or whether the same sample was measured repeatedly
☐	☒	The statistical test(s) used AND whether they are one- or two-sided<br>*Only common tests should be described solely by name; describe more complex techniques in the Methods section.*
☒	☐	A description of all covariates tested
☐	☒	A description of any assumptions or corrections, such as tests of normality and adjustment for multiple comparisons
☐	☒	A full description of the statistical parameters including central tendency (e.g. means) or other basic estimates (e.g. regression coefficient) AND variation (e.g. standard deviation) or associated estimates of uncertainty (e.g. confidence intervals)
☐	☒	For null hypothesis testing, the test statistic (e.g. *F*, *t*, *r*) with confidence intervals, effect sizes, degrees of freedom and *P* value noted<br>*Give P values as exact values whenever suitable.*
☒	☐	For Bayesian analysis, information on the choice of priors and Markov chain Monte Carlo settings
☒	☐	For hierarchical and complex designs, identification of the appropriate level for tests and full reporting of outcomes
☒	☐	Estimates of effect sizes (e.g. Cohen's *d*, Pearson's *r*), indicating how they were calculated

Our web collection on statistics for biologists contains articles on many of the points above.

Software and code

Policy information about availability of computer code

Data collection	All software is commercially or freely available, literature references are in the Methods section. Attune (version: 8.01.1) for flow cytometry, Odyssey version 3.0 for western blot, Invitrogen EVOS FL Auto2 imaging system, ZEN software (Carl Zeiss) confocal imaging.
Data analysis	All software is commercially or freely available, literature references are in the Methods section. Prism (Version: 10.2.3), flowJo (Version: 10.10.0), ImageStudioLite (LICObio).

For manuscripts utilizing custom algorithms or software that are central to the research but not yet described in published literature, software must be made available to editors and reviewers. We strongly encourage code deposition in a community repository (e.g. GitHub). See the Nature Portfolio guidelines for submitting code & software for further information.

Data

Policy information about availability of data

All manuscripts must include a data availability statement. This statement should provide the following information, where applicable:

- Accession codes, unique identifiers, or web links for publicly available datasets
- A description of any restrictions on data availability
- For clinical datasets or third party data, please ensure that the statement adheres to our policy

All other data supporting the findings of this study are in the Supplementary Figures. Source data are provided with this paper. Source data include uncropped western blot scans, flow cytometry and qPCR data.

Research involving human participants, their data, or biological material

Policy information about studies with human participants or human data. See also policy information about sex, gender (identity/presentation), and sexual orientation and race, ethnicity and racism.

Reporting on sex and gender	not involved.
Reporting on race, ethnicity, or other socially relevant groupings	not involved.
Population characteristics	not involved.
Recruitment	not involved.
Ethics oversight	not involved.

Note that full information on the approval of the study protocol must also be provided in the manuscript.

Field-specific reporting

Please select the one below that is the best fit for your research. If you are not sure, read the appropriate sections before making your selection.

- [x] Life sciences
- [ ] Behavioural & social sciences
- [ ] Ecological, evolutionary & environmental sciences

For a reference copy of the document with all sections, see nature.com/documents/nr-reporting-summary-flat.pdf

Life sciences study design

All studies must disclose on these points even when the disclosure is negative.

Sample size	All of the flow cytometery data and qPCR experiments are done in triplicates, and repeated at least twice. Western blot experiments have been repeated at least twice.
Data exclusions	No data were excluded.
Replication	All attempts at replication were successful.
Randomization	Randomization was not necessary for this study because cell lines are carefully validated and characterized, and proper controls are included. There were no human or animal subjects used in this study. Randomization is not typically used in this field.
Blinding	Blinding was not necessary for this study, as results in this study did not require subjective judgment. Blinding is not typically used in this field.

Reporting for specific materials, systems and methods

We require information from authors about some types of materials, experimental systems and methods used in many studies. Here, indicate whether each material, system or method listed is relevant to your study. If you are not sure if a list item applies to your research, read the appropriate section before selecting a response.

Materials & experimental systems

n/a	Involved in the study
[ ]	[x] Antibodies
[ ]	[x] Eukaryotic cell lines
[x]	[ ] Palaeontology and archaeology
[x]	[ ] Animals and other organisms
[x]	[ ] Clinical data
[x]	[ ] Dual use research of concern
[x]	[ ] Plants

Methods

n/a	Involved in the study
[x]	[ ] ChIP-seq
[ ]	[x] Flow cytometry
[x]	[ ] MRI-based neuroimaging

Antibodies

Antibodies used	anti-ATF4 (ProteinTech, 28657-1-AP, rabbit, 1:1000), anti-[illegible]-actin (ProteinTech, 66009-1-Ig, mouse, 1:5000), anti-[illegible]-actin (ProteinTech, 81115-1-RR, rabbit, 1:5000), anti-COX IV (Invitrogen, MA5-17279, mouse, 1:2000), anti-V5 (Thermo Fisher Scientific, R96025, mouse, 1:1000), anti-eIF2[illegible] (ProteinTech, 11170-1-AP, Rabbit, 1:1000), anti-GFP (Roche, 11814460001, mouse, 1:1,000),

anti-HSPD1 (Invitrogen, MA3-012, mouse, 1:2000), anti-LC3 (ProteinTech, 14600-1-AP, Rabbit, 1:500), anti-phospho-eIF2�(ser51) (Cell Signaling Technology, 3398S, Rabbit, 1:1000), anti-Parkin (Invitrogen, 702785, rabbit, 1:500), anti-PINK1 (ProteinTech, 23274-1-AP, rabbit, 1:500), anti-PINK1 (CST, 6946S, rabbit, 1:1000), anti-SDHB (Invitrogen, MA5-26936, mouse, 1:1000), anti-TIMM23 (Proteintech, 67535-1-Ig, mouse, 1:1000), anti-MFN1 (Invitrogen, PA5-38042, rabbit, 1:1000), anti-MFN2 (Proteintech, 12186-1-AP, rabbit, 1:1000), anti-phospho-Ubiquitin (CST, 62802S, rabbit, 1:1000), anti-OMA1 (Proteintech, 17116-1-AP, rabbit, 1:1000), anti-HRI (Proteintech, 20499-1-AP, rabbit, 1:1000), anti-VDAC (Cell Signaling Technology, 4661S, Rabbit, 1:1000). Antibodies used in Supplementary Fig.4 were: mouse monoclonal anti-EEA1 (BD Biosciences, Cat# 610456, 1:4000 dilution), mouse monoclonal anti-Kinectin (Santa Cruz Biotechnology, sc-374576, 1:5000), rabbit monoclonal anti-PERK (Cell Signaling Technology, 3192, 1 :2000), mouse monoclonal anti-GM130 (BD Biosciences, 610822, 1:2000), mouse monoclonal anti-LAMP2 (Santa Cruz Biotechnology, sc-18822, 1:5000), mouse monoclonal anti-calnexin (BD Biosciences, 610524, 1:4000 dilution), rabbit polyclonal anti-HRI (Proteintech, 20499-1-AP, 1 :2000), mouse monoclonal anti-calreticulin (BD Biosciences, 612137, 1:4000 dilution), rabbit monoclonal anti-ATF4 (Cell Signaling Technology, 11815, 1 :2000), rabbit monoclonal anti-Tubulin (Cell Signaling Technology, 2128, 1 :10000), rabbit polyclonal anti-DELE1 (Thermo Fisher Scientific, PA5-57712, 1 :1000), rabbit monoclonal anti-Syntaxin6 (Cell Signaling Technology, 2869, 1:5000), rabbit monoclonal anti-Phospho-eIF2a (Ser51) (Cell Signaling Technology, 3398, 1 :2000), rabbit monoclonal eIF2� (Cell Signaling Technology, 5324, 1 :5000), mouse monoclonal anti-GAPDH (Sigma-Aldrich, G8795, 1 :10000) and rabbit polyclonal anti-VDAC (Millipore, AB10527, 1:10000).

Validation

All antibodies used in this study were pre�validated by their respective manufacturers. In our experiments, they behaved as expected, detecting proteins at the appropriate molecular weights.
anti-ATF4 (ProteinTech, 28657-1-AP, rabbit, 1:1000)
https://www.ptglab.com/products/ATF4-Antibody-28657-1-AP.htm
anti-b-actin (ProteinTech, 66009-1-Ig, mouse, 1:5000)
https://www.ptglab.com/products/Pan-Actin-Antibody-66009-1-Ig.htm
anti-b-actin (ProteinTech, 81115-1-RR, rabbit, 1:5000)
https://www.ptglab.com/products/beta-actin-Antibody-81115-1-RR.htm
anti-COX IV (Invitrogen, MA5-17279, mouse, 1:2000)
https://www.thermofisher.com/antibody/product/COX4-Antibody-clone-GT6310-Monoclonal/MA5-17279
anti-V5 (Thermo Fisher Scientific, R96025, mouse, 1:1000)
https://www.thermofisher.com/antibody/product/V5-Tag-Antibody-clone-SV5-Pk1-Monoclonal/R960-25
anti-eIF2a (ProteinTech, 11170-1-AP, Rabbit, 1:1000)
https://www.ptglab.com/products/EIF2S1-Antibody-11170-1-AP.htm
anti-GFP (Roche, 11814460001, mouse, 1:1,000)
https://www.sigmaaldrich.com/US/en/product/roche/11814460001?
srsltid=AfmBOopz2LvsIsTaFh_XwYDEX-3eMxu8sXB03HOjNuaA99FIYlEz1A42
anti-HSPD1 (Invitrogen, MA3-012, mouse, 1:2000)
https://www.thermofisher.com/antibody/product/HSP60-Antibody-clone-4B9-89-Monoclonal/MA3-012
anti-LC3 (ProteinTech, 14600-1-AP, Rabbit, 1:500)
https://www.ptglab.com/products/MAP1LC3B-Antibody-14600-1-AP.htm?srsltid=AfmBOoqImfWuKiwmVA7XdXZrL6V3HPHqY-J9iyV9cAj4LrHwkSyPRBR6
anti-phospho-eIF2a(ser51) (Cell Signaling Technology, 3398S, Rabbit, 1:1000)
https://awsprod-www.cellsignal.com/products/primary-antibodies/phospho-eif2-alpha-ser51-d9g8-rabbit-monoclonal-antibody/3398
anti-Parkin (Invitrogen, 702785, rabbit, 1:500)
https://www.thermofisher.com/antibody/product/Parkin-Antibody-clone-21H24L9-Recombinant-Monoclonal/702785
anti-PINK1 (ProteinTech, 23274-1-AP, rabbit, 1:500)
https://www.ptglab.com/products/PINK1-Antibody-23274-1-AP.htm
anti-PINK1 (CST, 6946S, rabbit, 1:1000)
https://www.cellsignal.com/products/primary-antibodies/pink1-d8g3-rabbit-mab/6946
anti-SDHB (Invitrogen, MA5-26936, mouse, 1:1000)
https://www.thermofisher.com/antibody/product/SDHB-Antibody-clone-OTI3F5-Monoclonal/MA5-26936
anti-TIMM23 (Proteintech, 67535-1-Ig, mouse, 1:1000)
https://www.ptglab.com/products/Tim23-Antibody-67535-1-Ig.htm?
srsltid=AfmBOopxo5ScULcMRKTQpQZmd_sycx8uRPxnrPfbwanJn7b1Z0CZXP2U
anti-MFN1 (Invitrogen, PA5-38042, rabbit, 1:1000)
https://www.thermofisher.com/antibody/product/MFN1-Antibody-Polyclonal/PA5-38042
anti-MFN2 (Proteintech, 12186-1-AP, rabbit, 1:1000)
https://www.ptglab.com/products/MFN2-Antibody-12186-1-AP.htm
anti-phospho-Ubiquitin (CST, 62802S, rabbit, 1:1000)
https://www.cellsignal.com/products/primary-antibodies/phospho-ubiquitin-ser65-e2j6t-rabbit-mab/62802
anti-OMA1 (Proteintech, 17116-1-AP, rabbit, 1:1000)
https://www.ptglab.com/products/OMA1-Antibody-17116-1-AP.htm
anti-HRI (Proteintech, 20499-1-AP, rabbit, 1:1000)
https://www.ptglab.com/products/EIF2AK1-Antibody-20499-1-AP.htm
anti-VDAC (Cell Signaling Technology, 4661S, Rabbit, 1:1000)
https://www.cellsignal.com/products/primary-antibodies/vdac-d73d12-rabbit-mab/4661
Antibodies used in Supplementary Fig.4 were: mouse monoclonal anti-EEA1 (BD Biosciences, Cat# 610456, 1:4000 dilution)
https://www.bdbiosciences.com/en-us/products/reagents/microscopy-imaging-reagents/immunofluorescence-reagents/purified-mouse-anti-eea1.610456
mouse monoclonal anti-Kinectin (Santa Cruz Biotechnology, sc-374576, 1:5000)
https://www.scbt.com/p/kinectin-1-antibody-g-5?srsltid=AfmBOoq6IF7xmSd2YL_sPiV0LfbQEUzXNZMuwL7nbVEV0DdHac4ES-_j
rabbit monoclonal anti-PERK (Cell Signaling Technology, 3192, 1 :2000)
https://www.cellsignal.com/products/primary-antibodies/perk-c33e10-rabbit-mab/3192
mouse monoclonal anti-GM130 (BD Biosciences, 610822, 1:2000)
https://www.bdbiosciences.com/en-us/products/reagents/microscopy-imaging-reagents/immunofluorescence-reagents/purified-mouse-anti-gm130.610822

mouse monoclonal anti-LAMP2 (Santa Cruz Biotechnology, sc-18822, 1:5000)
https://www.scbt.com/p/lamp-2-antibody-h4b4
mouse monoclonal anti-calnexin (BD Biosciences, 610524, 1:4000 dilution)
https://www.bdbiosciences.com/en-dk/products/reagents/western-blotting-and-molecular-reagents/western-blot-reagents/purified-mouse-anti-calnexin.610524?tab=product_details
rabbit polyclonal anti-HRI (Proteintech, 20499-1-AP, 1 :2000)
https://www.ptglab.com/products/EIF2AK1-Antibody-20499-1-AP.htm
mouse monoclonal anti-calreticulin (BD Biosciences, 612137, 1:4000 dilution)
https://www.bdbiosciences.com/en-us/products/reagents/microscopy-imaging-reagents/immunofluorescence-reagents/purified-mouse-anti-calreticulin.612137
rabbit monoclonal anti-ATF4 (Cell Signaling Technology, 11815, 1 :2000)
https://www.cellsignal.com/products/primary-antibodies/atf-4-d4b8-rabbit-mab/11815
rabbit monoclonal anti-Tubulin (Cell Signaling Technology, 2128, 1 :10000)
https://www.cellsignal.com/products/primary-antibodies/beta-tubulin-9f3-rabbit-monoclonal-antibody/2128?srsltid=AfmBOope_sDS0Fo2eIcVvQQtUmr1bmQeLYQ6vT7XD1mHeJDosx0KIERm
rabbit polyclonal anti-DELE1 (Thermo Fisher Scientific, PA5-57712, 1 :1000)
https://www.thermofisher.com/antibody/product/DELE-Antibody-Polyclonal/PA5-57712
rabbit monoclonal anti-Syntaxin6 (Cell Signaling Technology, 2869, 1:5000)
https://www.cellsignal.com/products/primary-antibodies/syntaxin-6-c34b2-rabbit-mab/2869
rabbit monoclonal anti-Phospho-eIF2a (Ser51) (Cell Signaling Technology, 3398, 1 :2000)
https://awsprod-www.cellsignal.com/products/primary-antibodies/phospho-eif2-alpha-ser51-d9g8-rabbit-monoclonal-antibody/3398
rabbit monoclonal eIF2a (Cell Signaling Technology, 5324, 1 :5000)
https://www.cellsignal.com/products/primary-antibodies/eif2-alpha-d7d3-rabbit-monoclonal-antibody/5324?srsltid=AfmBOopXRo2wqjeVrxkzJjacnPkxc50AmQXnRmpnRs8vwX5XvhXAaNtw
mouse monoclonal anti-GAPDH (Sigma-Aldrich, G8795, 1 :10000)
https://www.sigmaaldrich.com/product/sigma/g8795
rabbit polyclonal anti-VDAC (Millipore, AB10527, 1:10000)
https://www.sigmaaldrich.com/US/en/product/mm/ab10527?srsltid=AfmBOor5K0W7Yk81Z-AJZ8aCiavss7l5zLEEcP7yn1-wqMgZkfPqxDrf

Eukaryotic cell lines

Policy information about cell lines and Sex and Gender in Research

Cell line source(s)

Most of the cell lines used in this study are generated using HEK293T (ATCC: CRL-3216) as parental cell line. We also used HeLa (ATCC CCL-2), K562 (ATCC CCL-243)cell lines.

Authentication

We did authentication on the K562 using CLA IdentiFiler Direct kit at Keck DNA Sequencing Facility at Yale. HEK293T and HeLa cell lines were not authenticated.

Mycoplasma contamination

All the cell lines used in this study are not contaminated with mycoplasma. These cell lines were subject to mycoplasma detection once every 6 months using in house PCR method.

Commonly misidentified lines
(See ICLAC register)

N/A

Plants

Seed stocks

Report on the source of all seed stocks or other plant material used. If applicable, state the seed stock centre and catalogue number. If plant specimens were collected from the field, describe the collection location, date and sampling procedures.

Novel plant genotypes

Describe the methods by which all novel plant genotypes were produced. This includes those generated by transgenic approaches, gene editing, chemical/radiation-based mutagenesis and hybridization. For transgenic lines, describe the transformation method, the number of independent lines analyzed and the generation upon which experiments were performed. For gene-edited lines, describe the editor used, the endogenous sequence targeted for editing, the targeting guide RNA sequence (if applicable) and how the editor was applied.

Authentication

Describe any authentication procedures for each seed stock used or novel genotype generated. Describe any experiments used to assess the effect of a mutation and, where applicable, how potential secondary effects (e.g. second site T-DNA insertions, mosiacism, off-target gene editing) were examined.

Flow Cytometry

Plots

Confirm that:

☒ The axis labels state the marker and fluorochrome used (e.g. CD4-FITC).

☒ The axis scales are clearly visible. Include numbers along axes only for bottom left plot of group (a 'group' is an analysis of identical markers).

☒ All plots are contour plots with outliers or pseudocolor plots.

☒ A numerical value for number of cells or percentage (with statistics) is provided.

Methodology

Sample preparation	Cells were trypsinized and resuspended in DMEM for flow cytometry.
Instrument	Attune Nxt
Software	Attune Flow cytometry (version: 8.01.1)
Cell population abundance	In this study, cell populations are differentiated by fluorescent markers. Internal non-fluorescent population is nicely separated from those with fluorescent markers. All the populations have sufficient events.
Gating strategy	For the Gating strategy, please see Extended Data Fig. 17.

☒ Tick this box to confirm that a figure exemplifying the gating strategy is provided in the Supplementary Information.
