# Supplementary material for: The integrated stress response suppresses PINK1-dependent mitophagy by preserving mitochondrial import efficiency
Source: Nat Commun. 2026 Apr 9;17:4838. doi: 10.1038/s41467-026-71630-6 (PMC13223273; doi:10.1038/s41467-026-71630-6)

Figure 1h

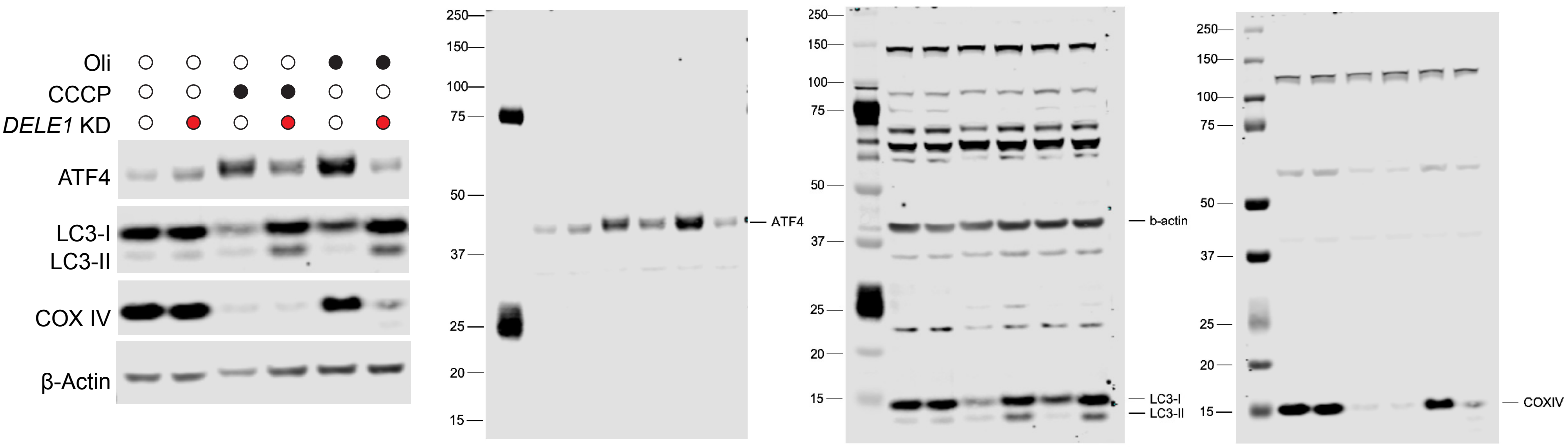

Figure 2a

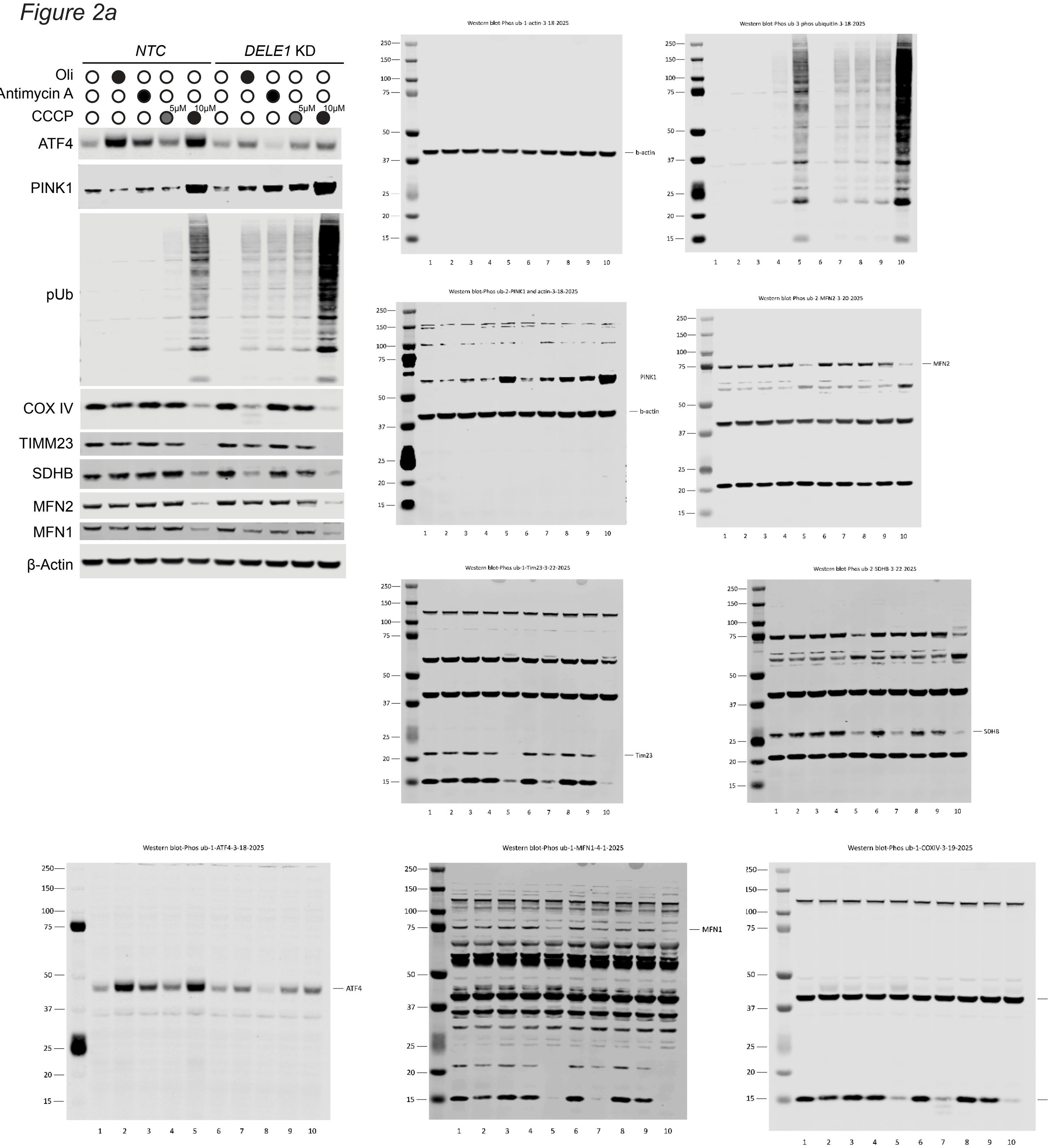

Figure 2c

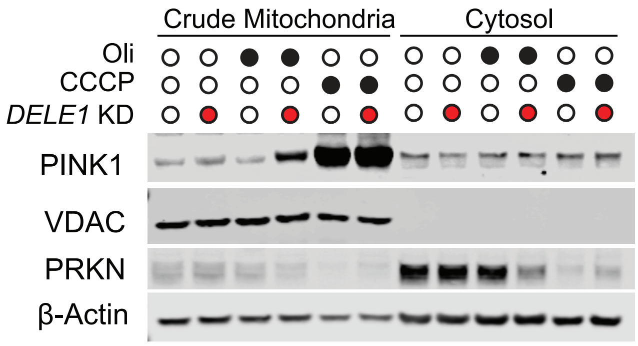

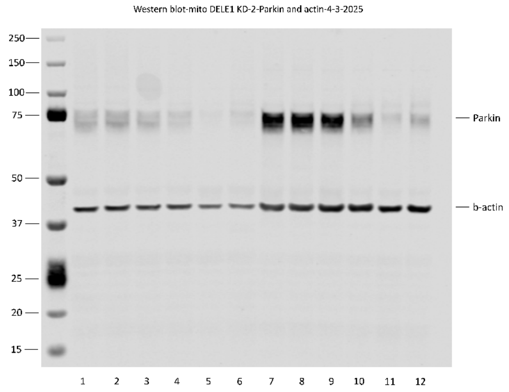

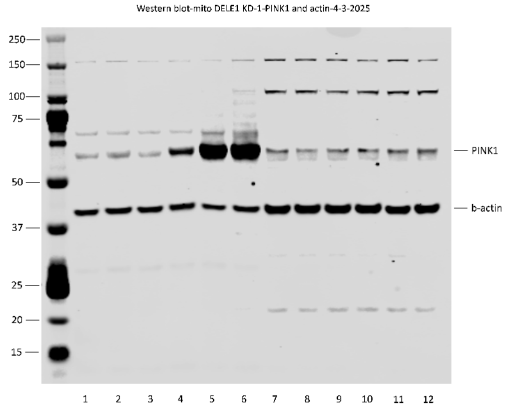

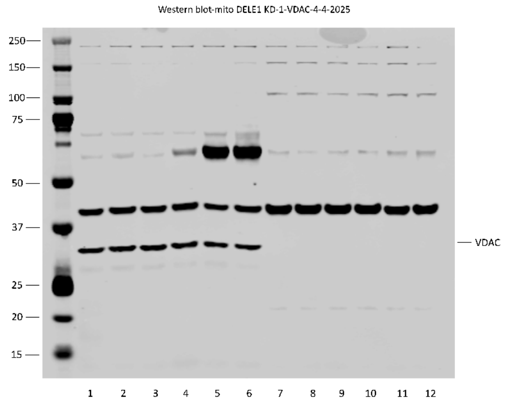

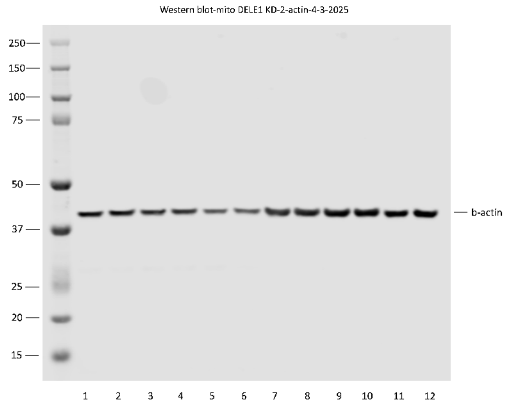

Figure 2d

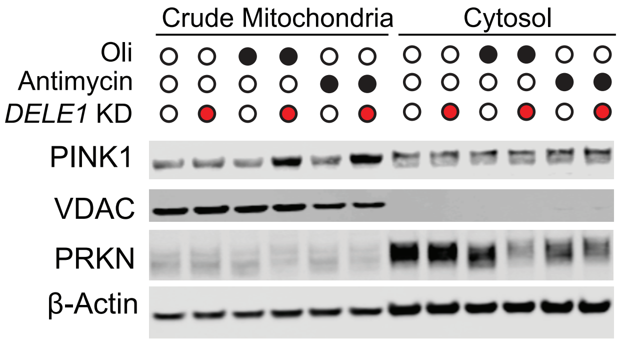

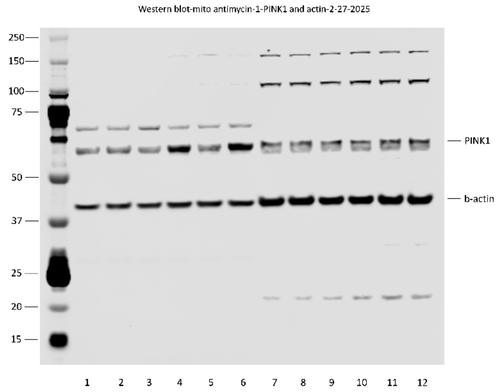

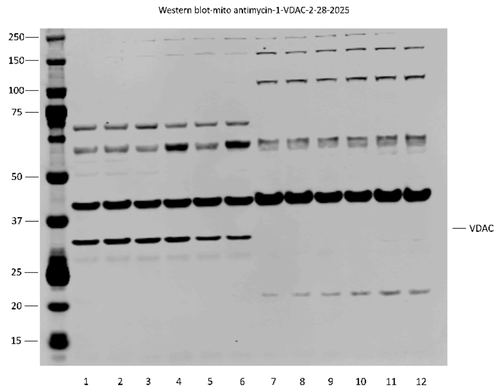

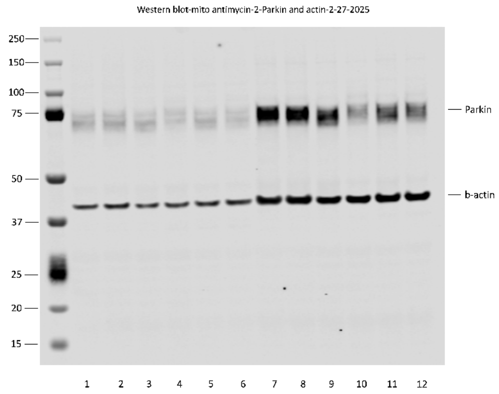

Figure 3a

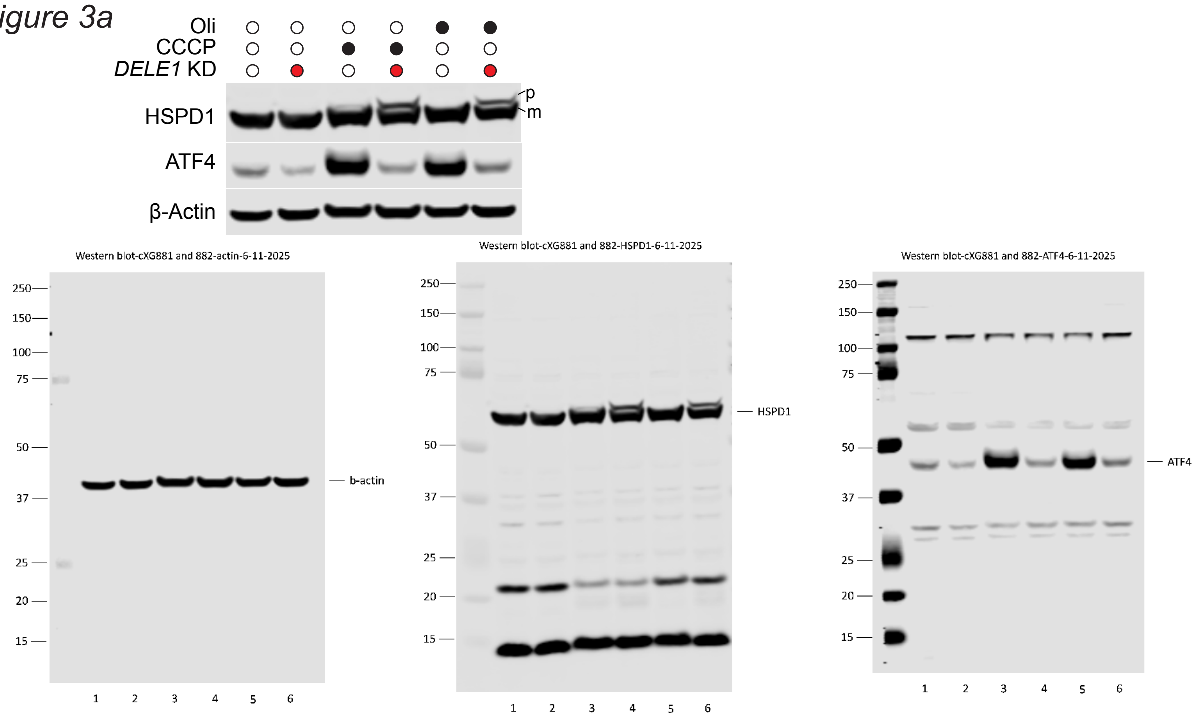

Figure 3b

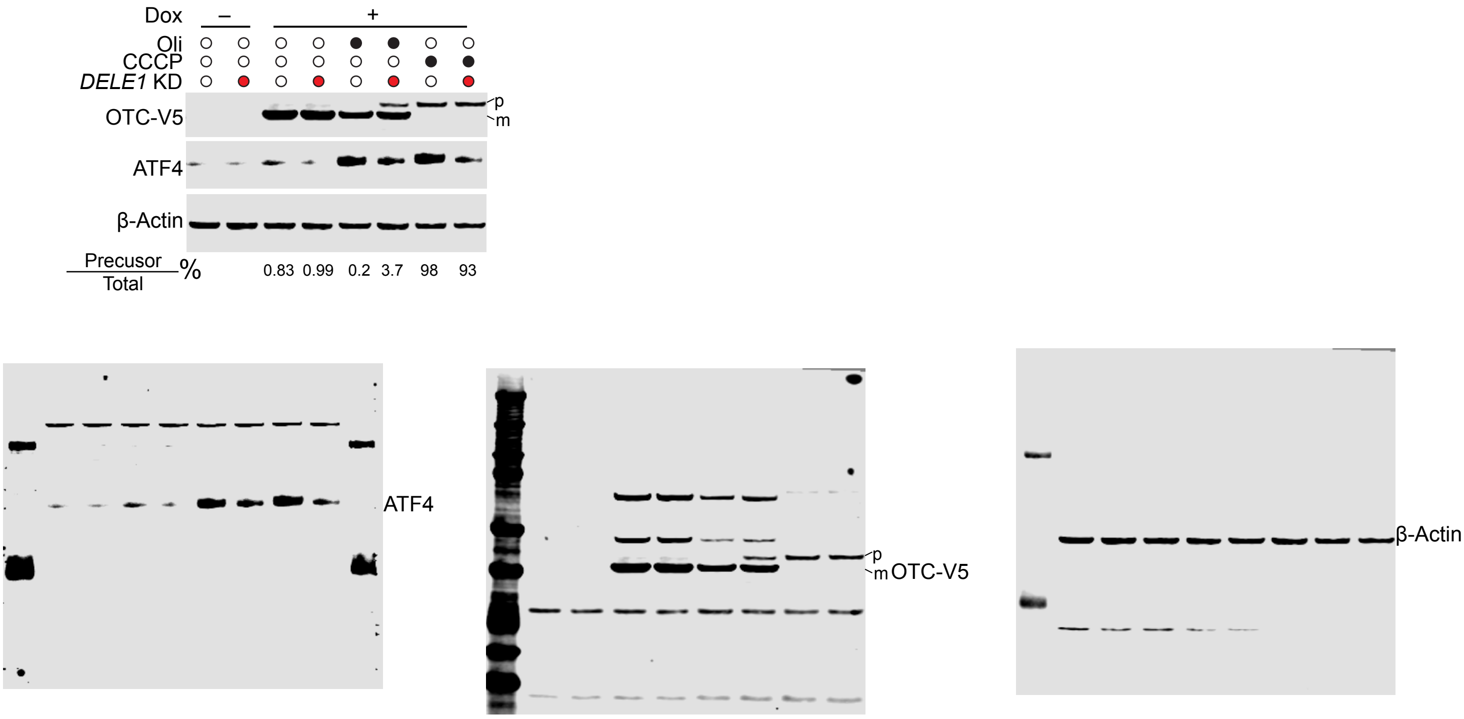

Figure 3i

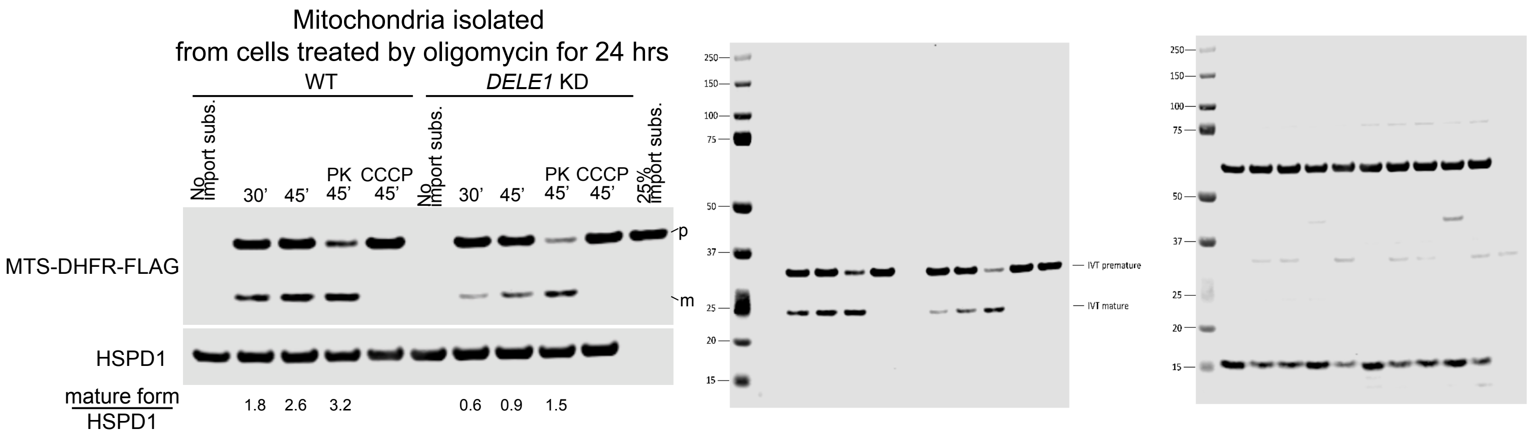

Figure 4b

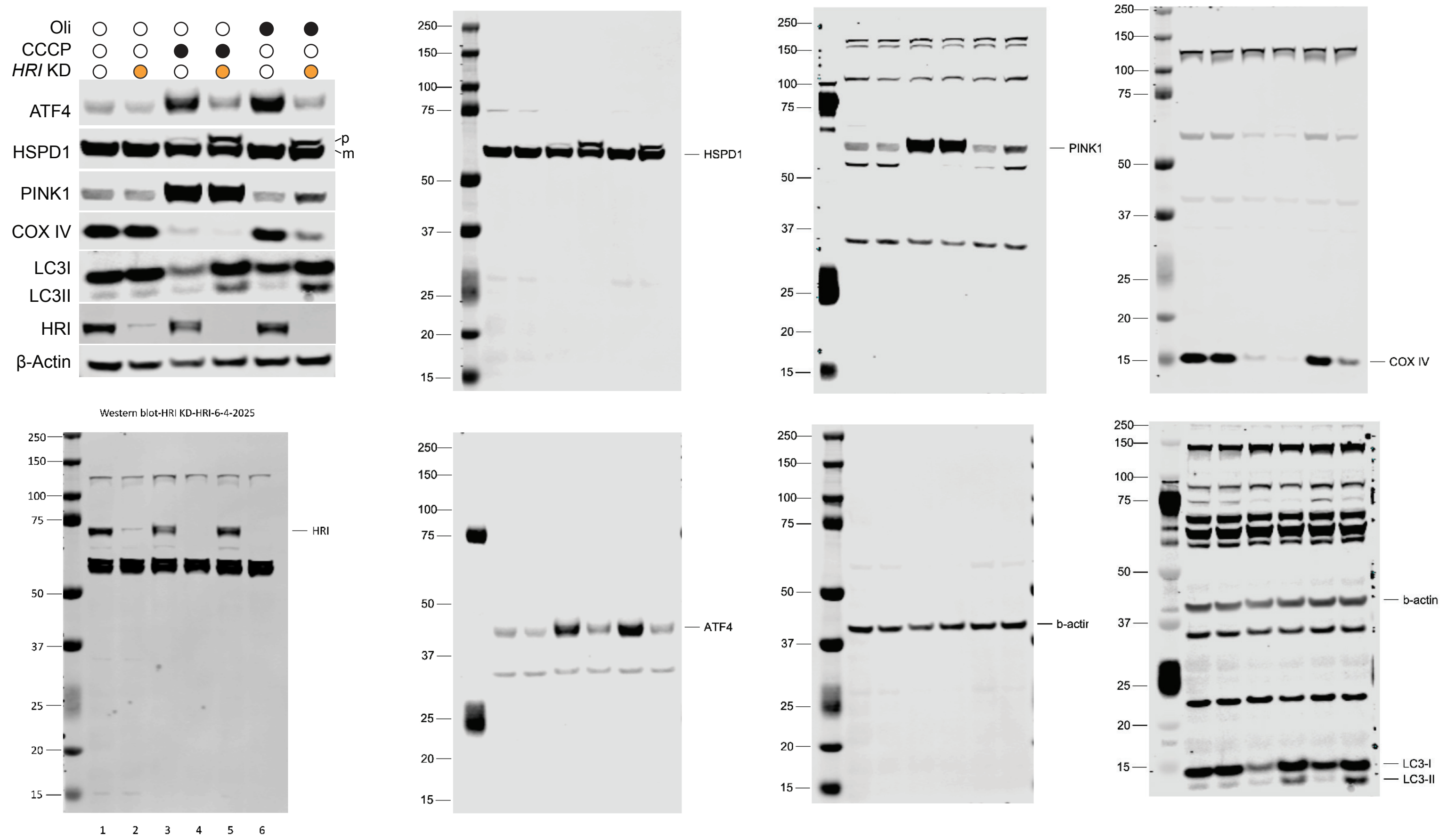

Figure 4d

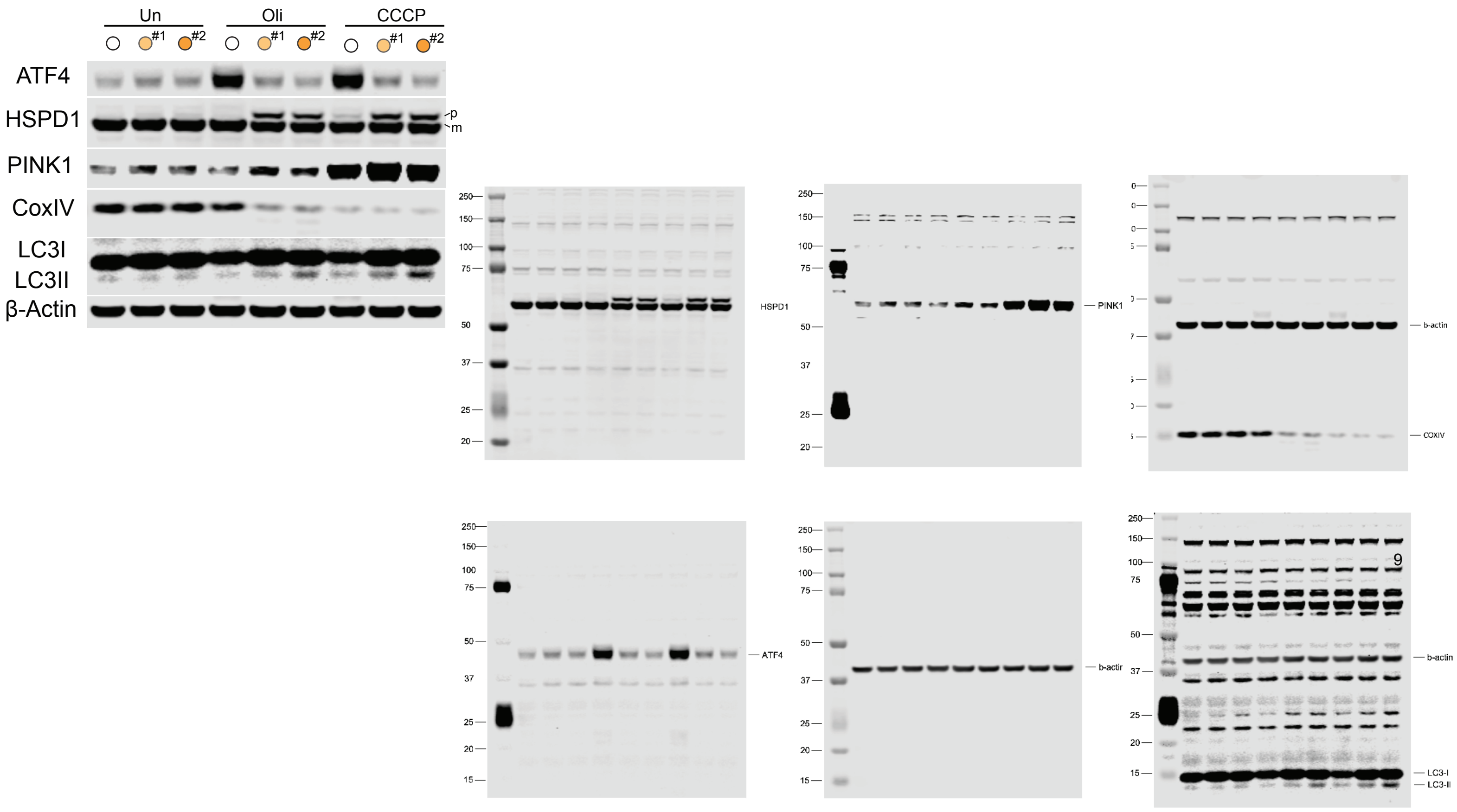

Figure 4f

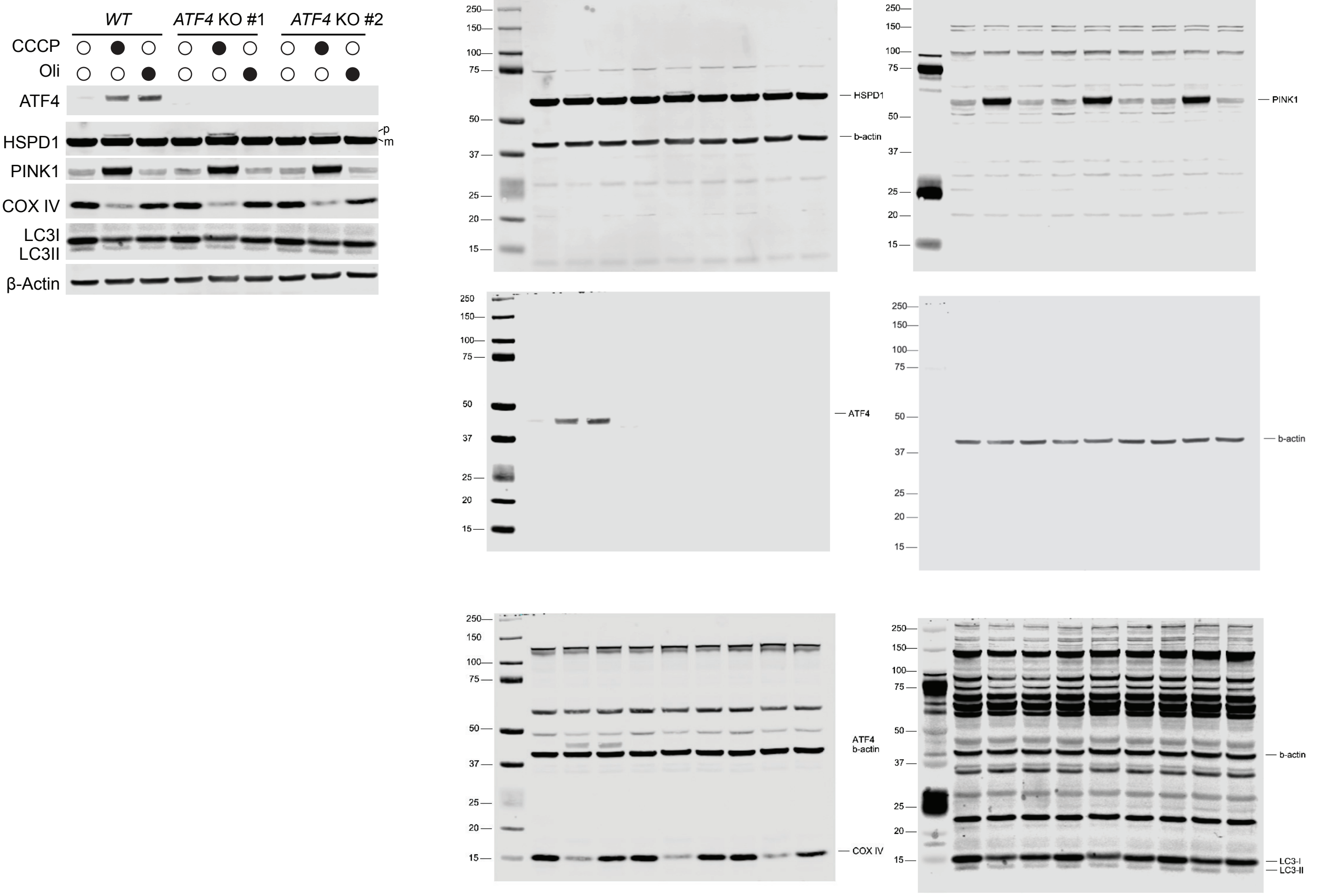

Figure 4j

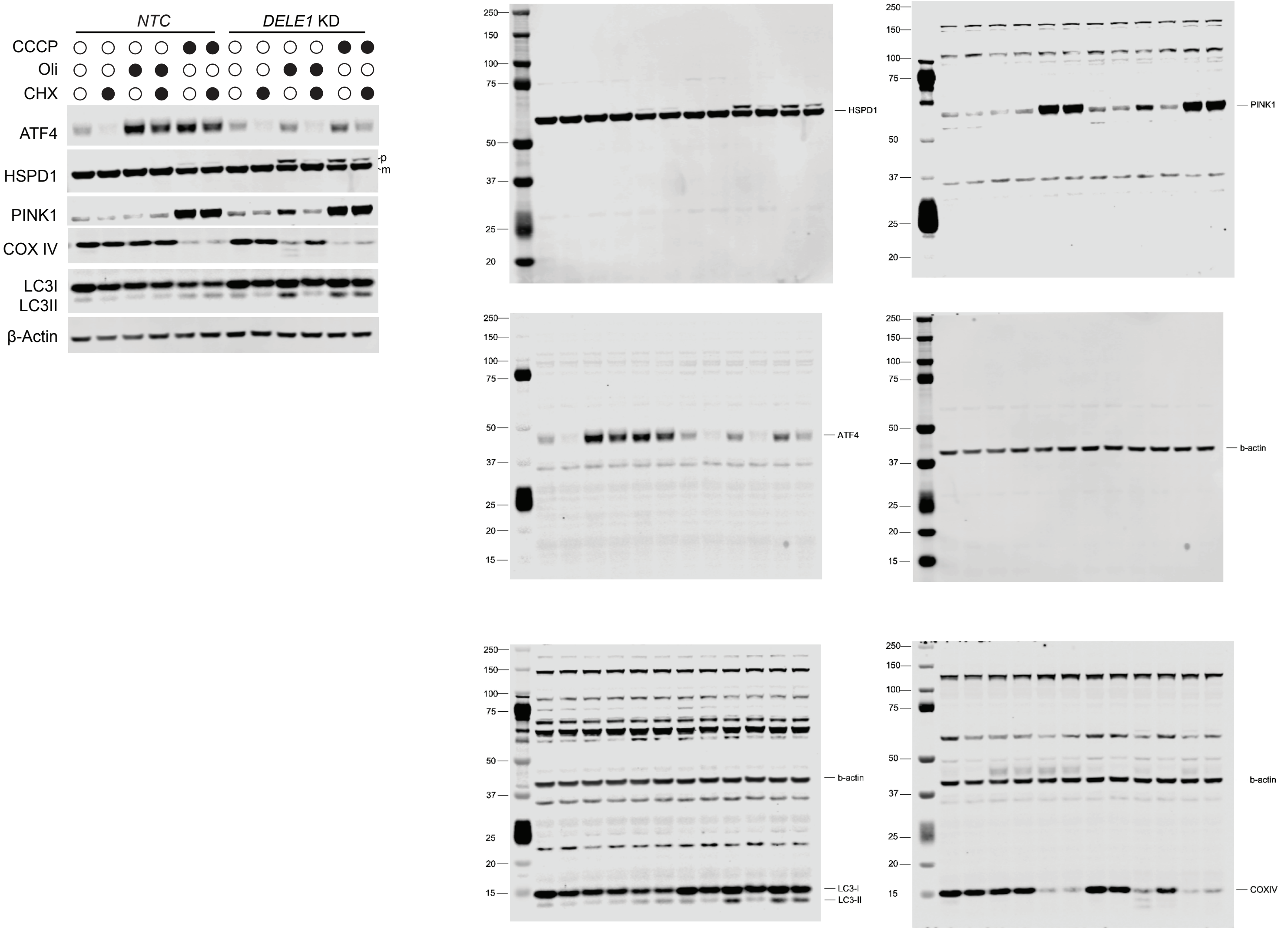

Figure 4i

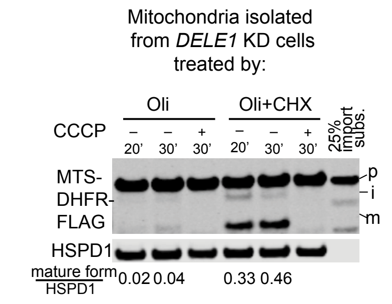

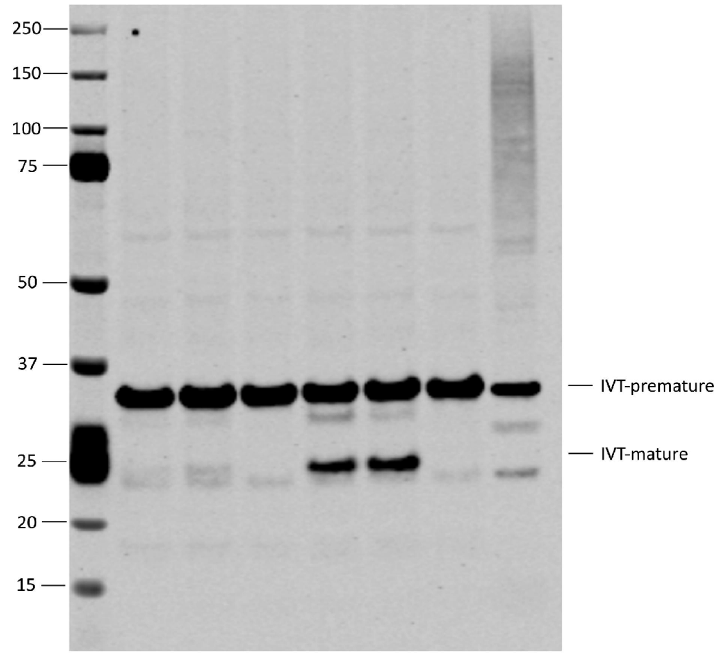

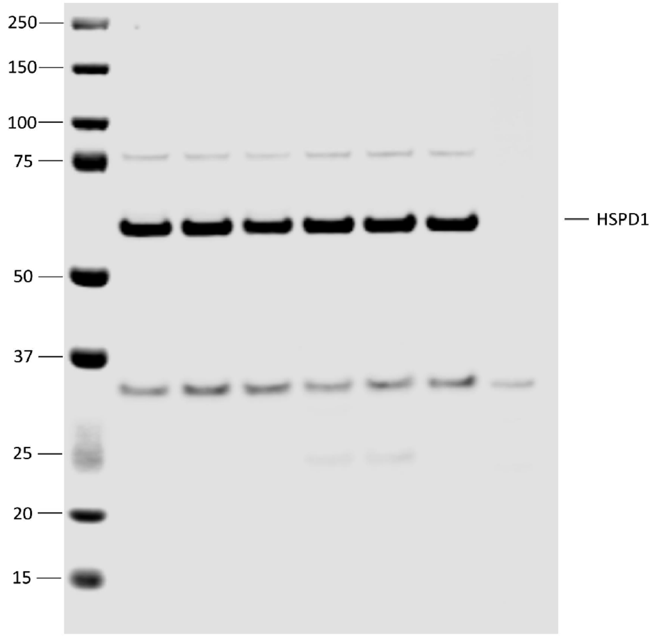

Figure 4m

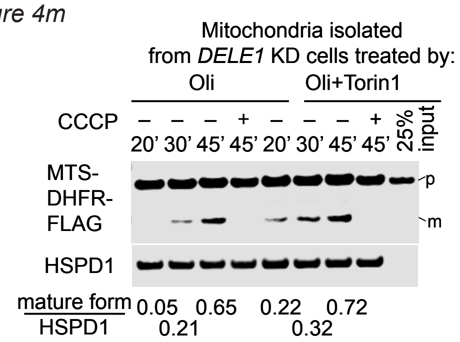

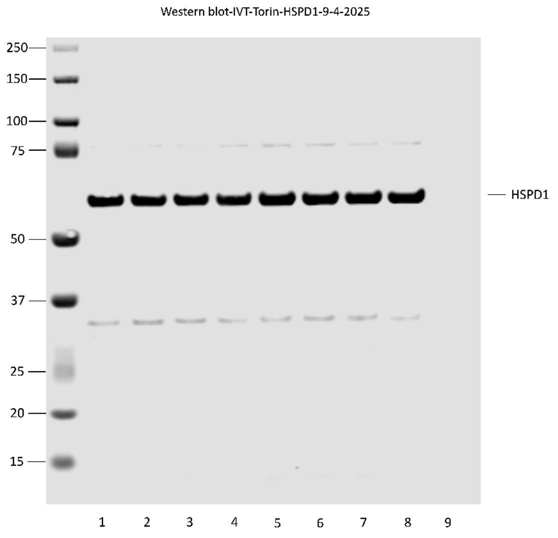

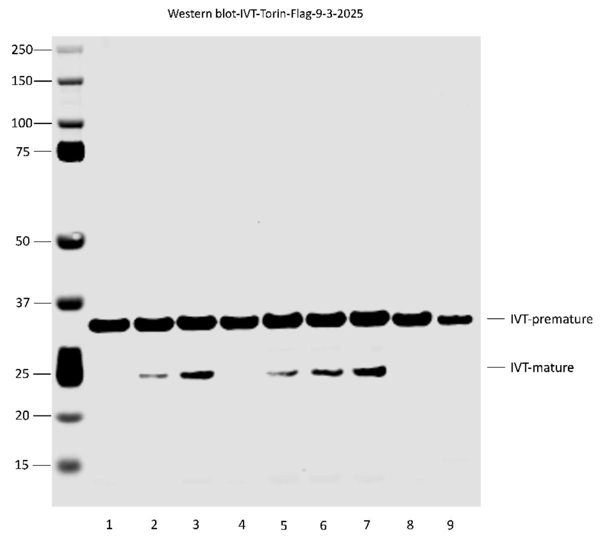

Figure 4n

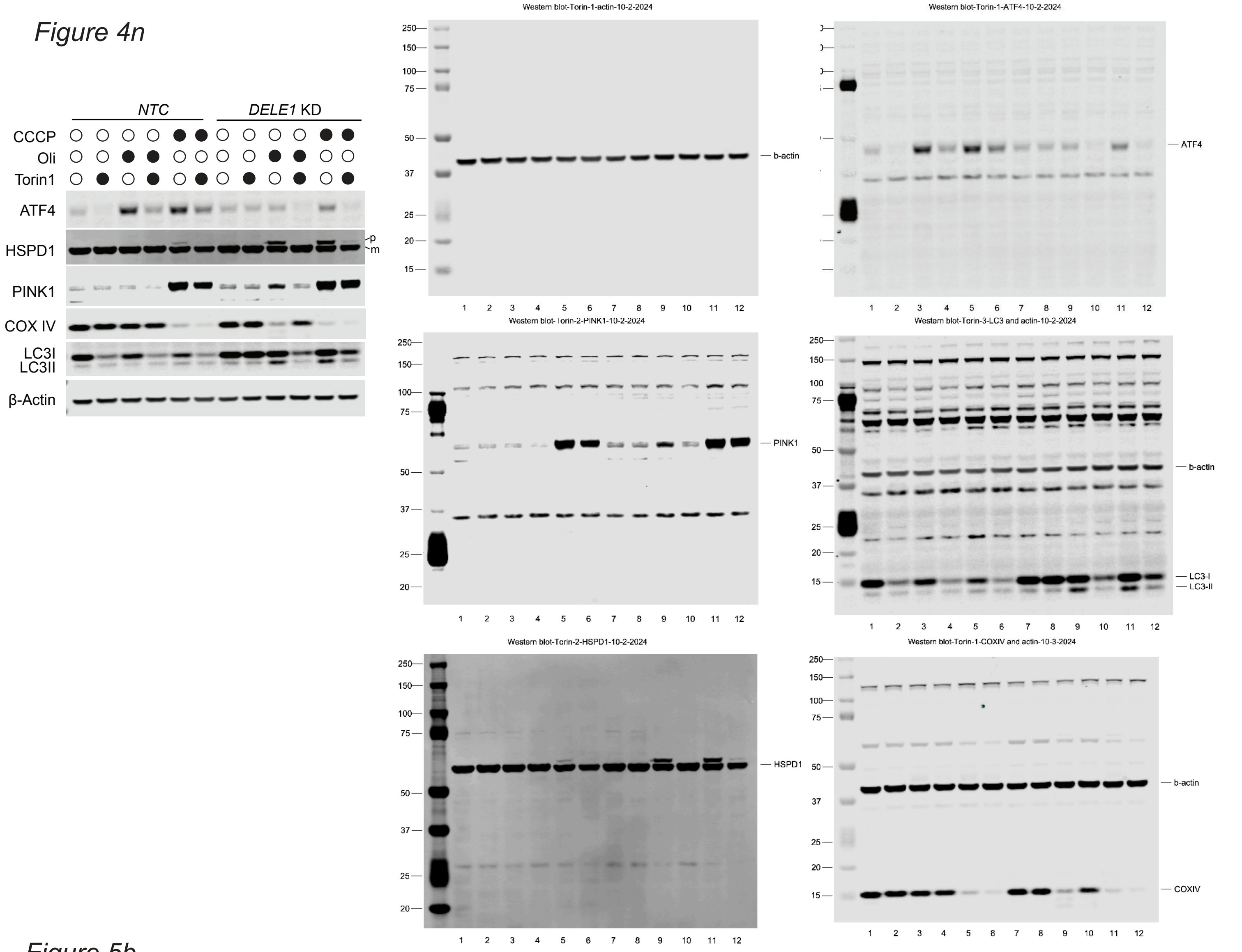

Figure 5b

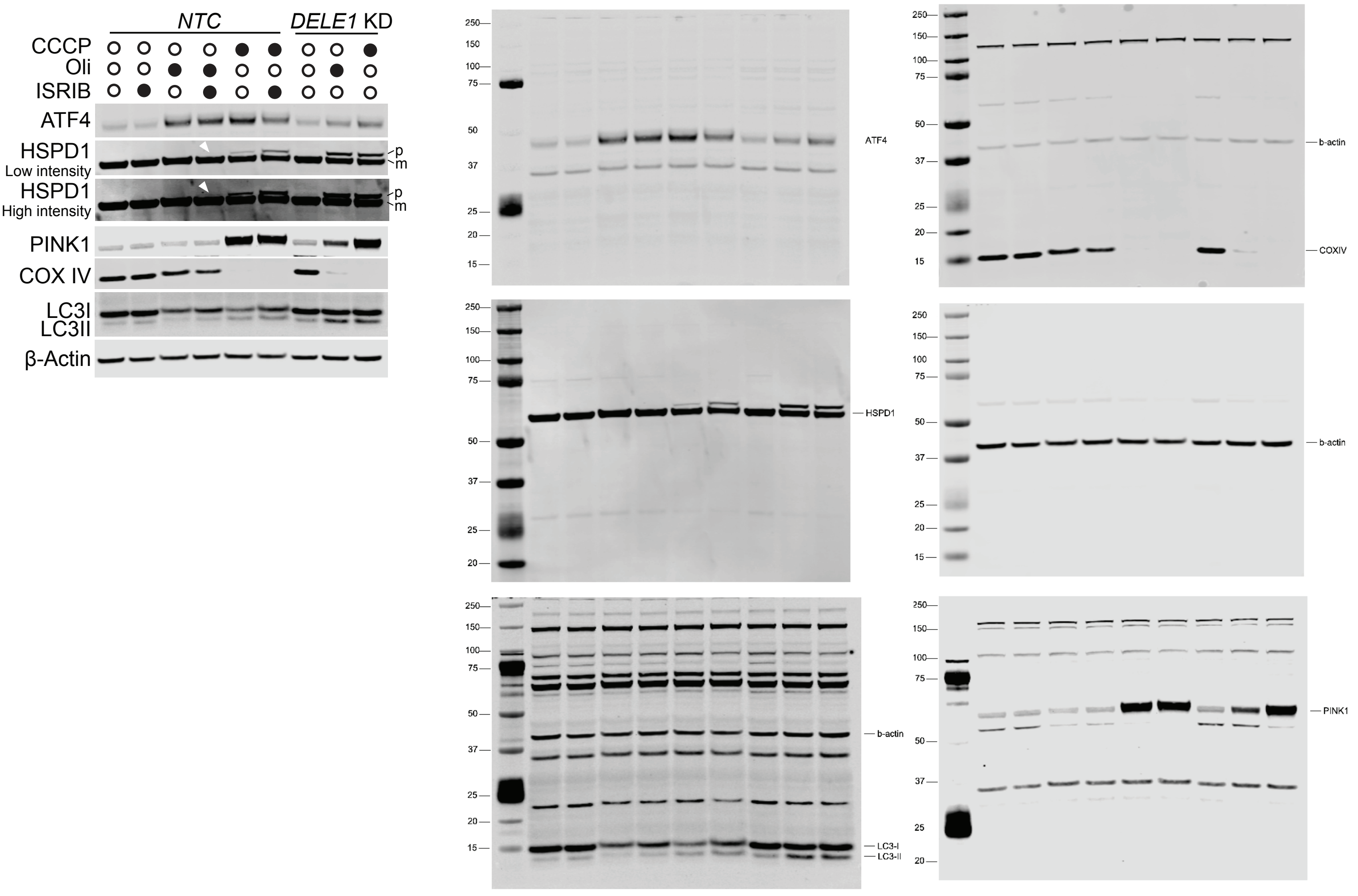

Figure 5d

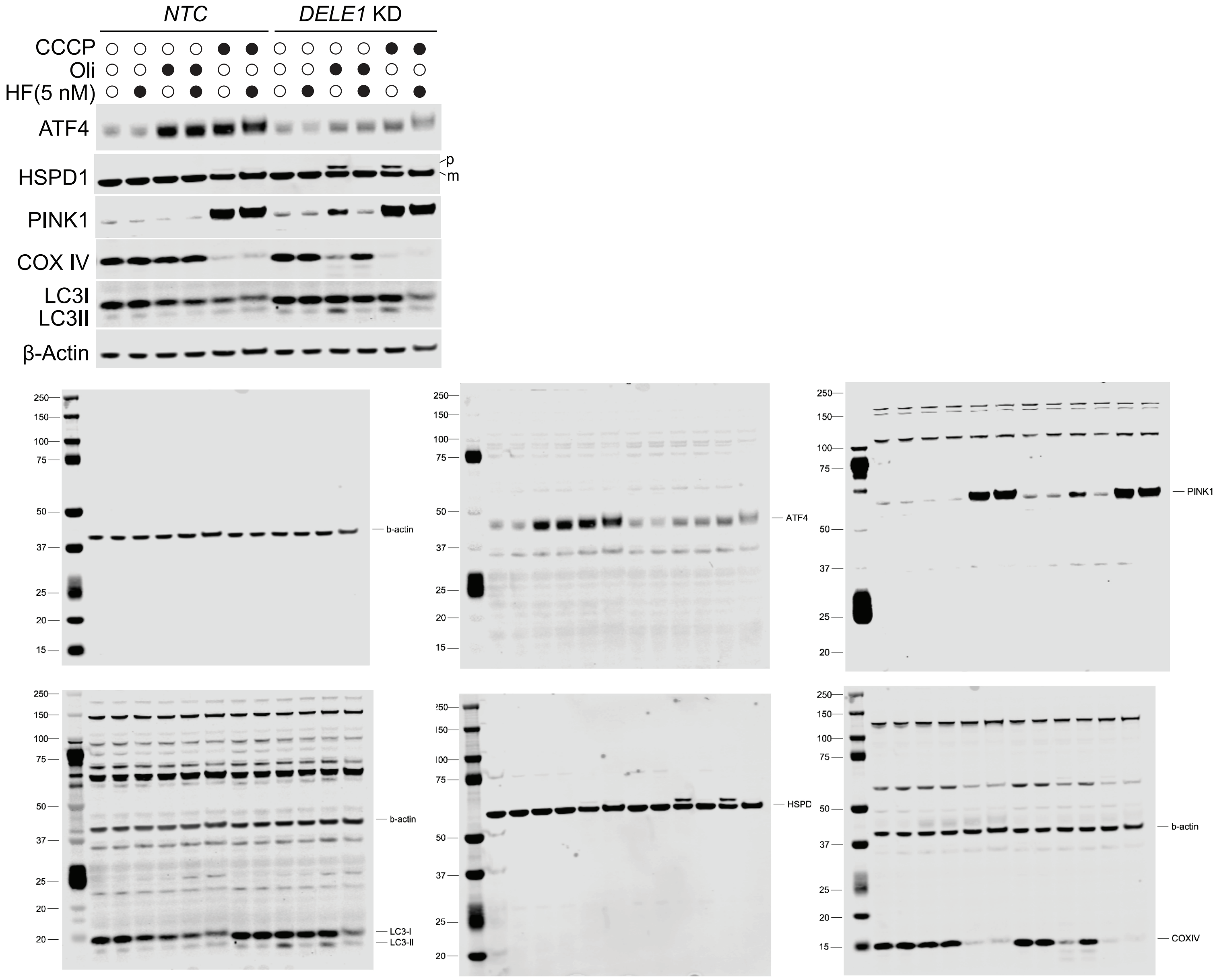

Figure 6b

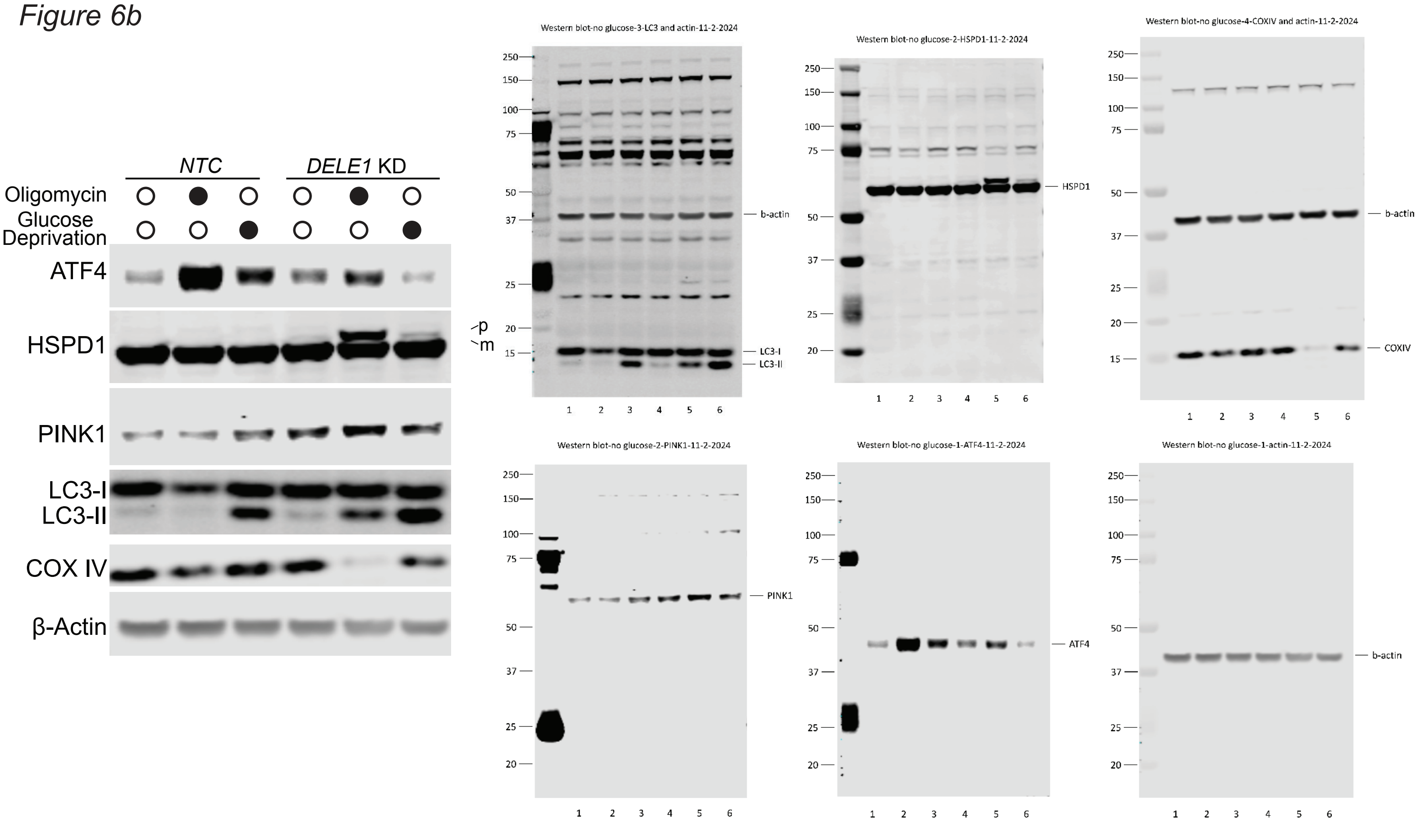

Supplementary Fig. 1b

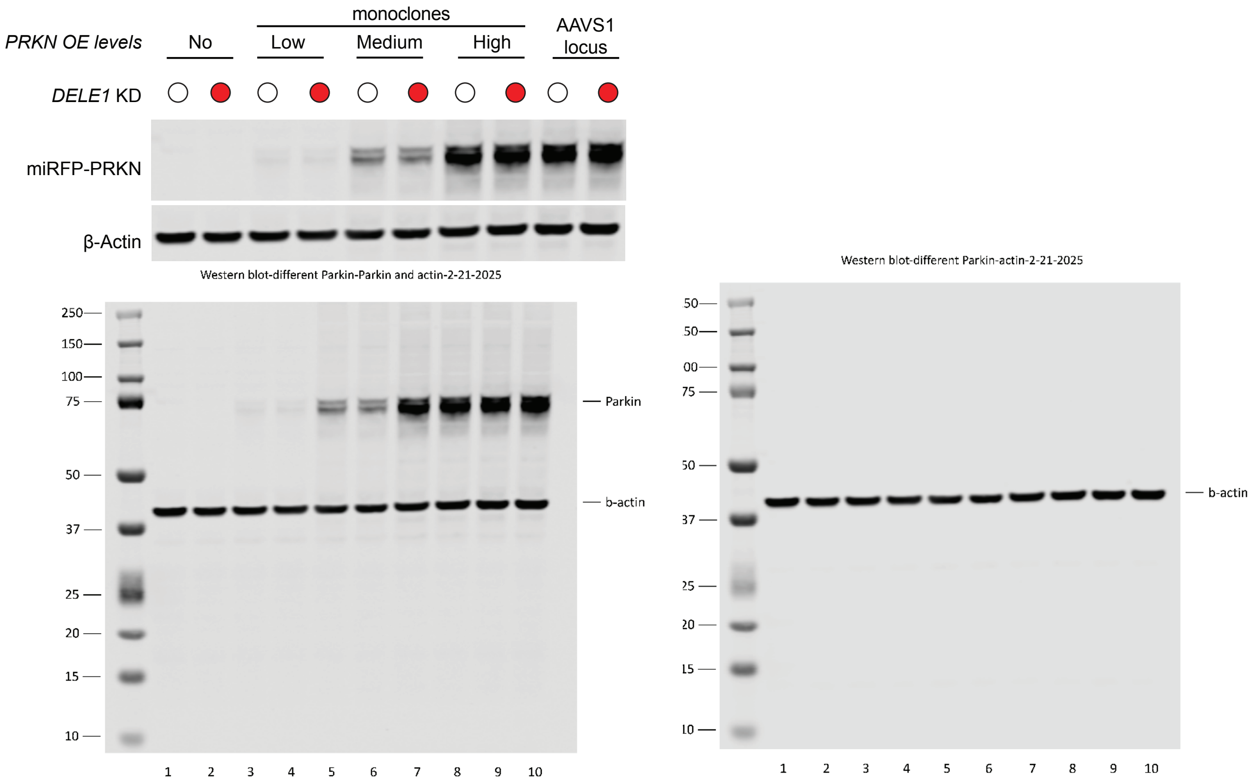

Supplementary Fig. 1h

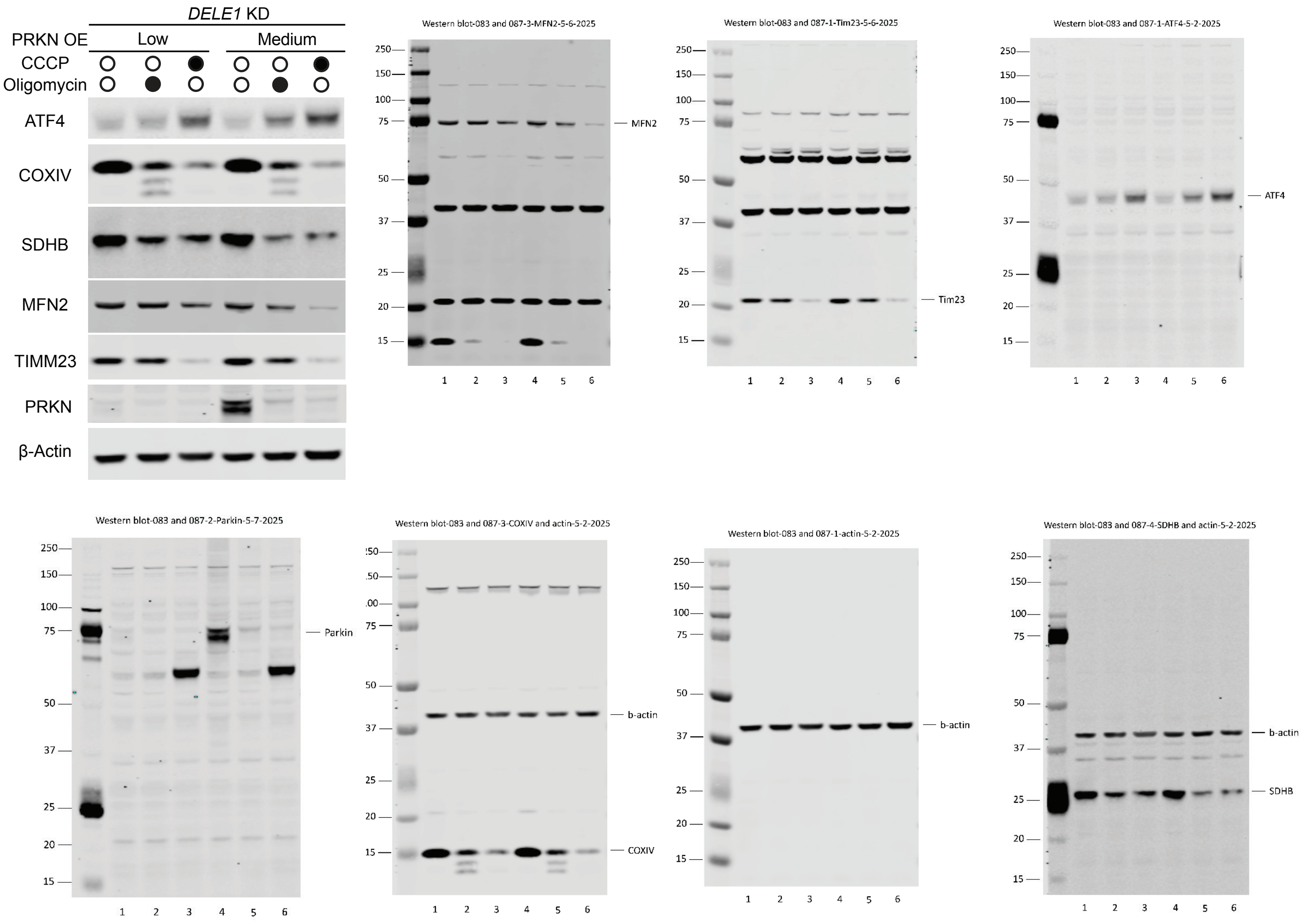

Supplementary Fig 2b

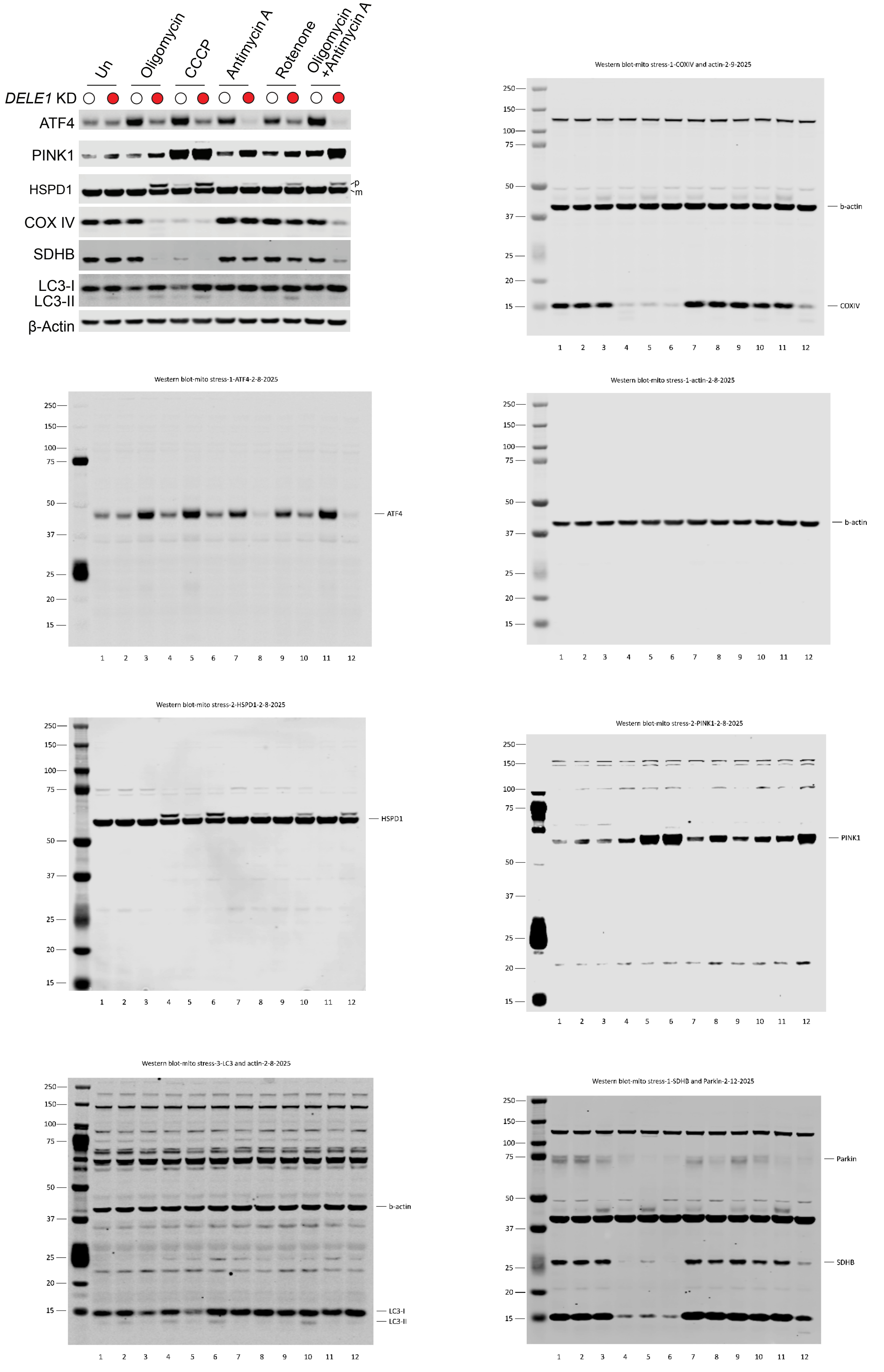

Supplementary Fig. 4a right

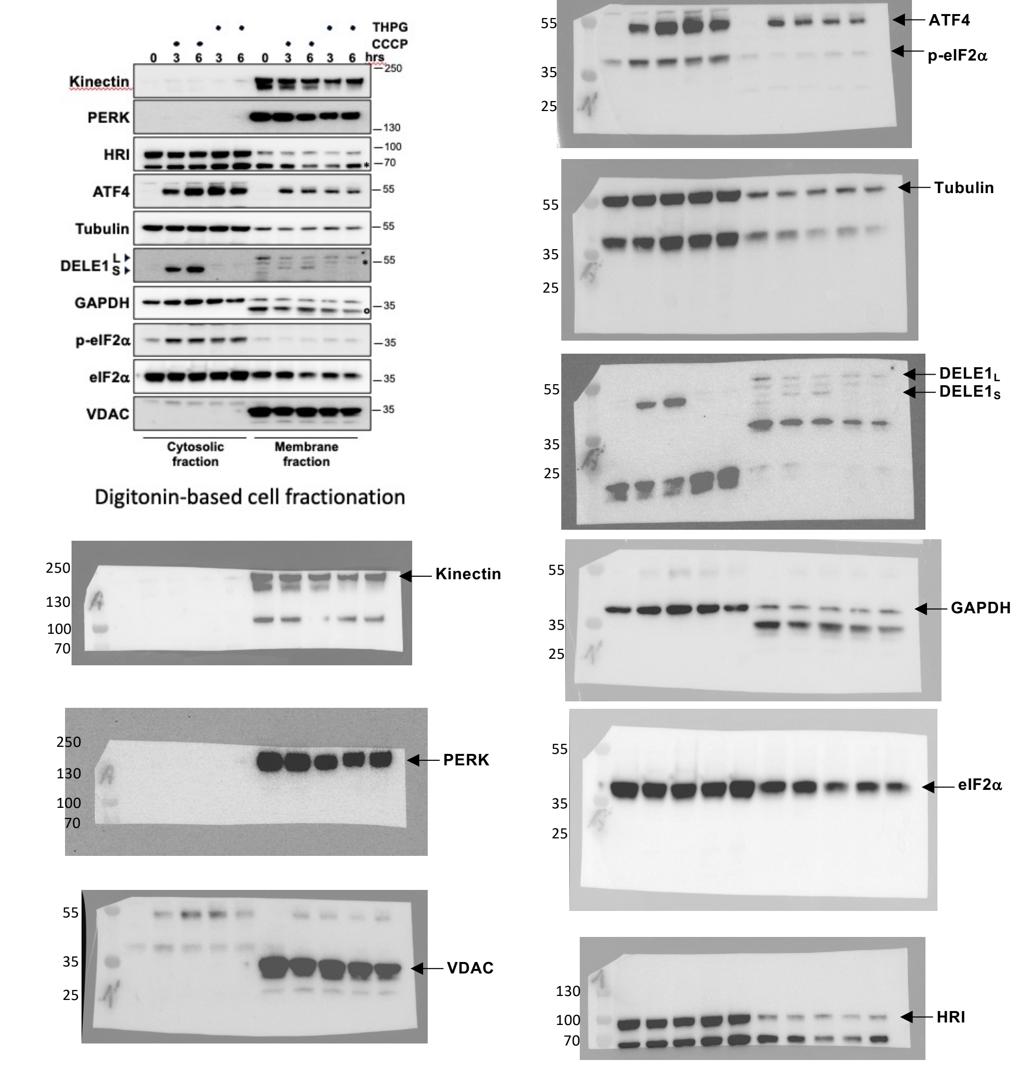

Supplementary Fig. 4b left

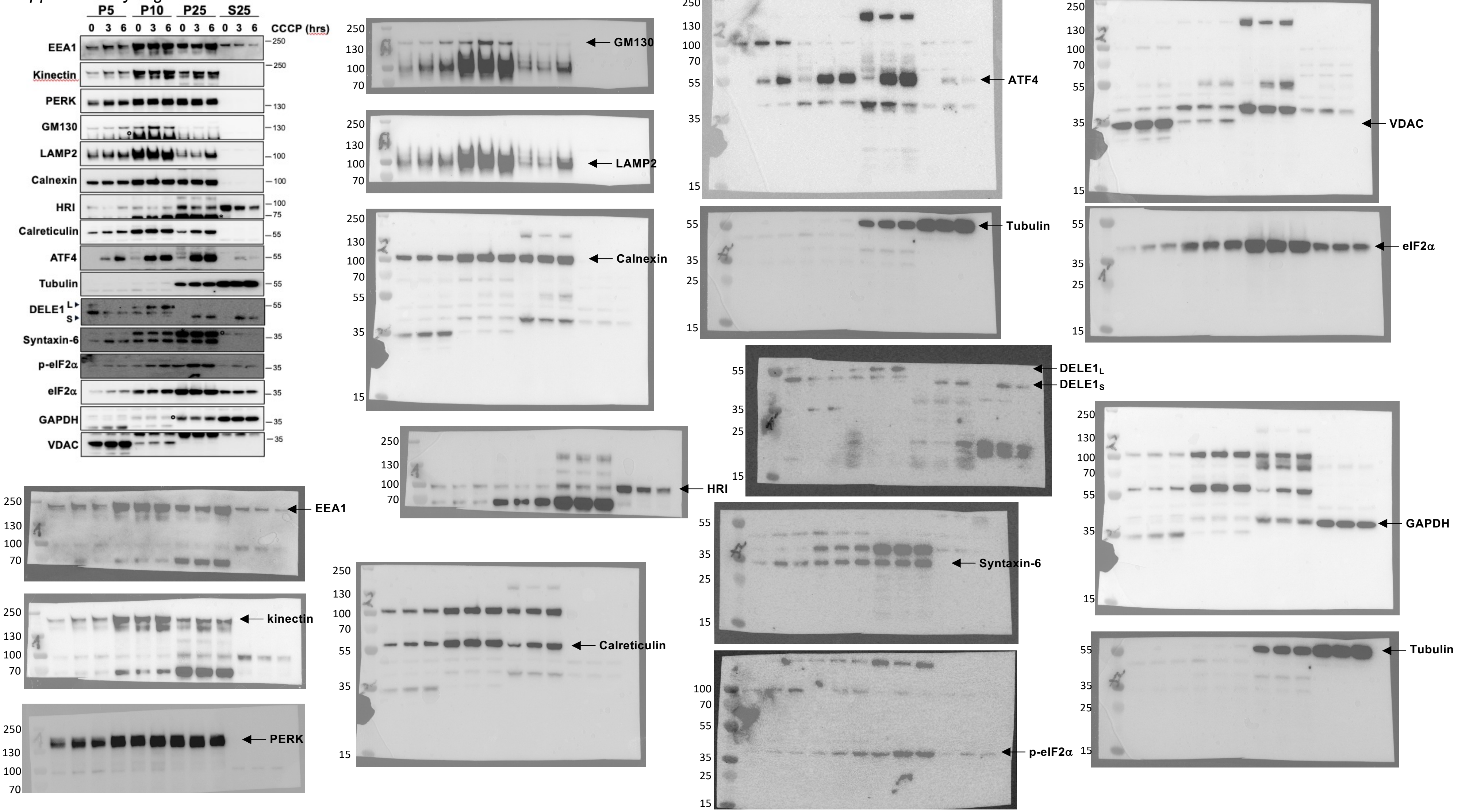

Supplementary Fig. 4b right

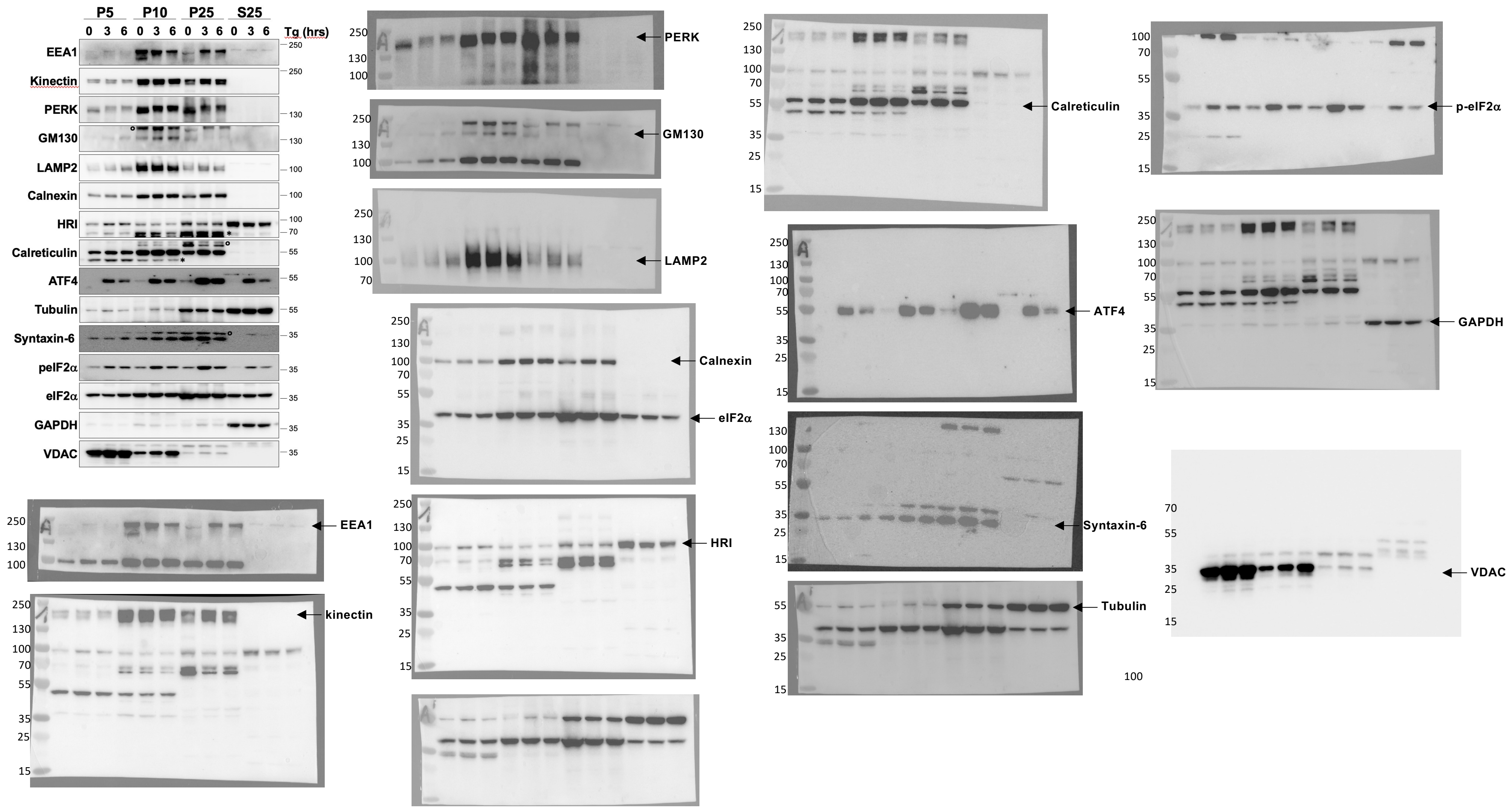

Supplementary Fig. 5

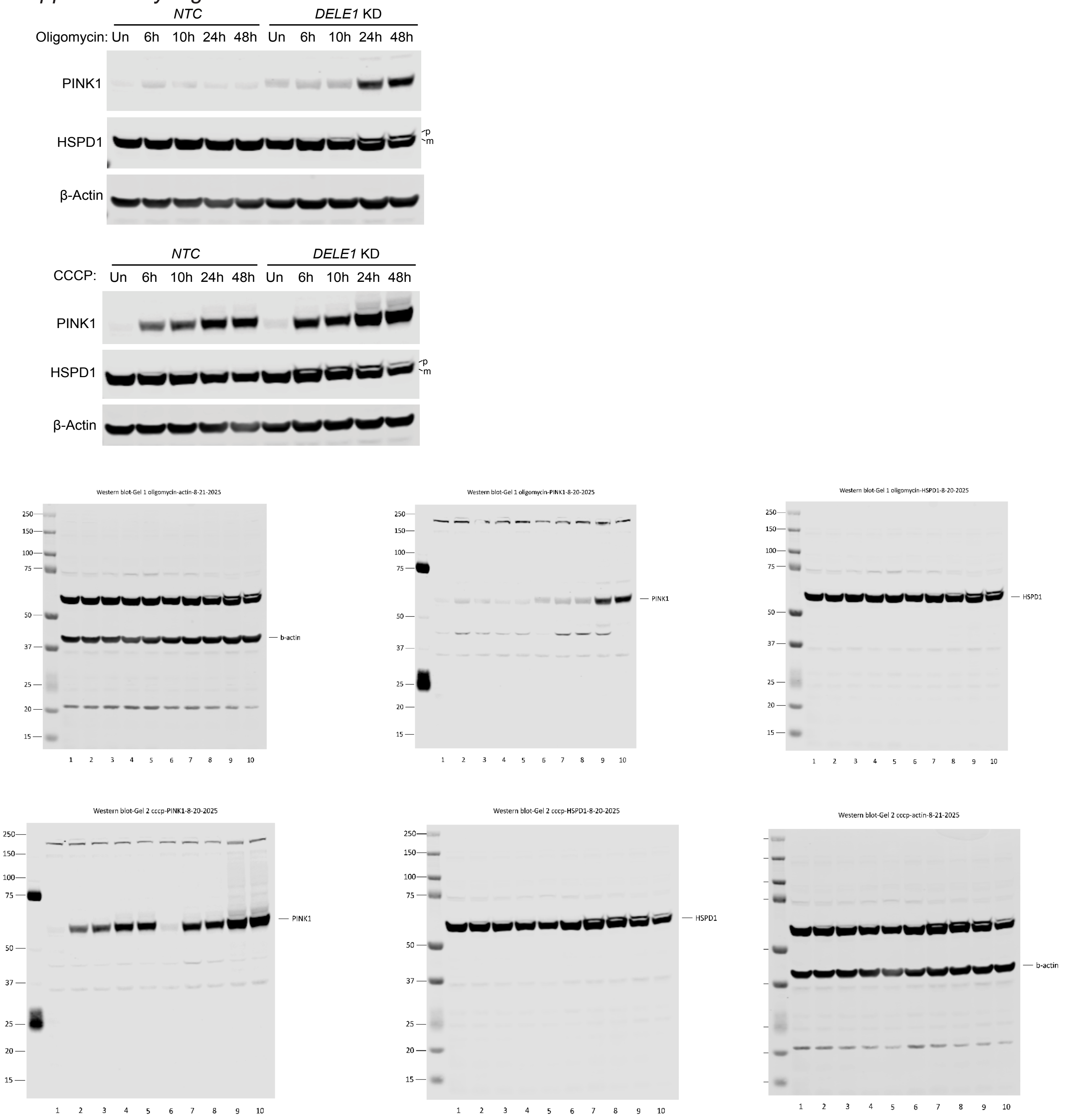

Supplementary Fig. 6a

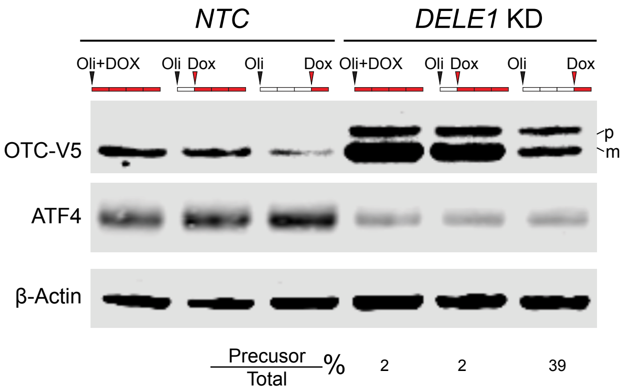

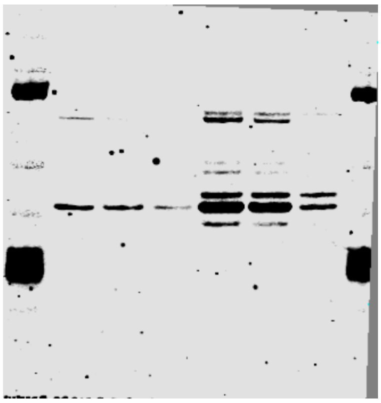

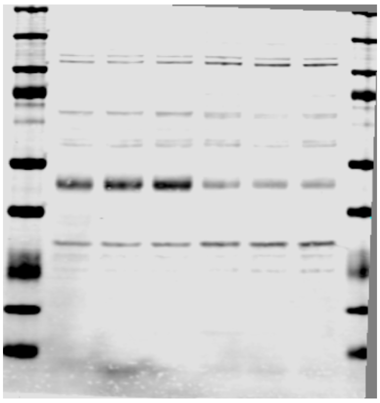

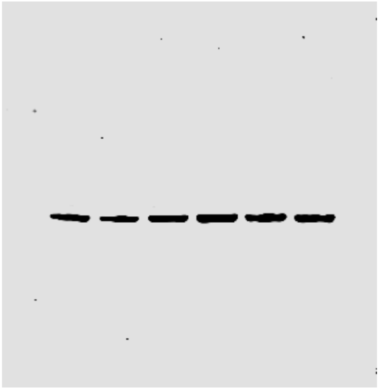

Supplementary Fig. 6b

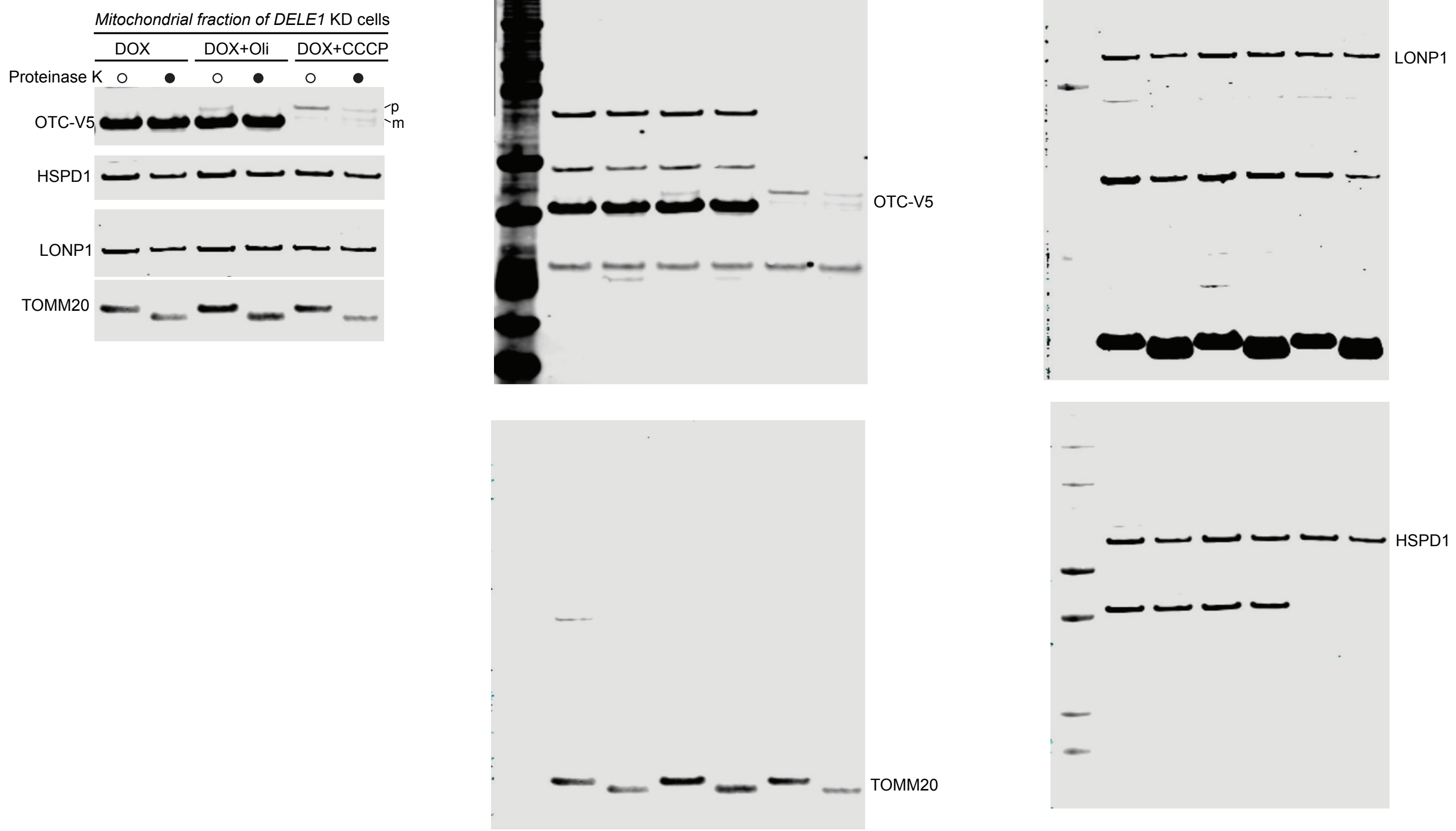

Supplementary Fig. 6c

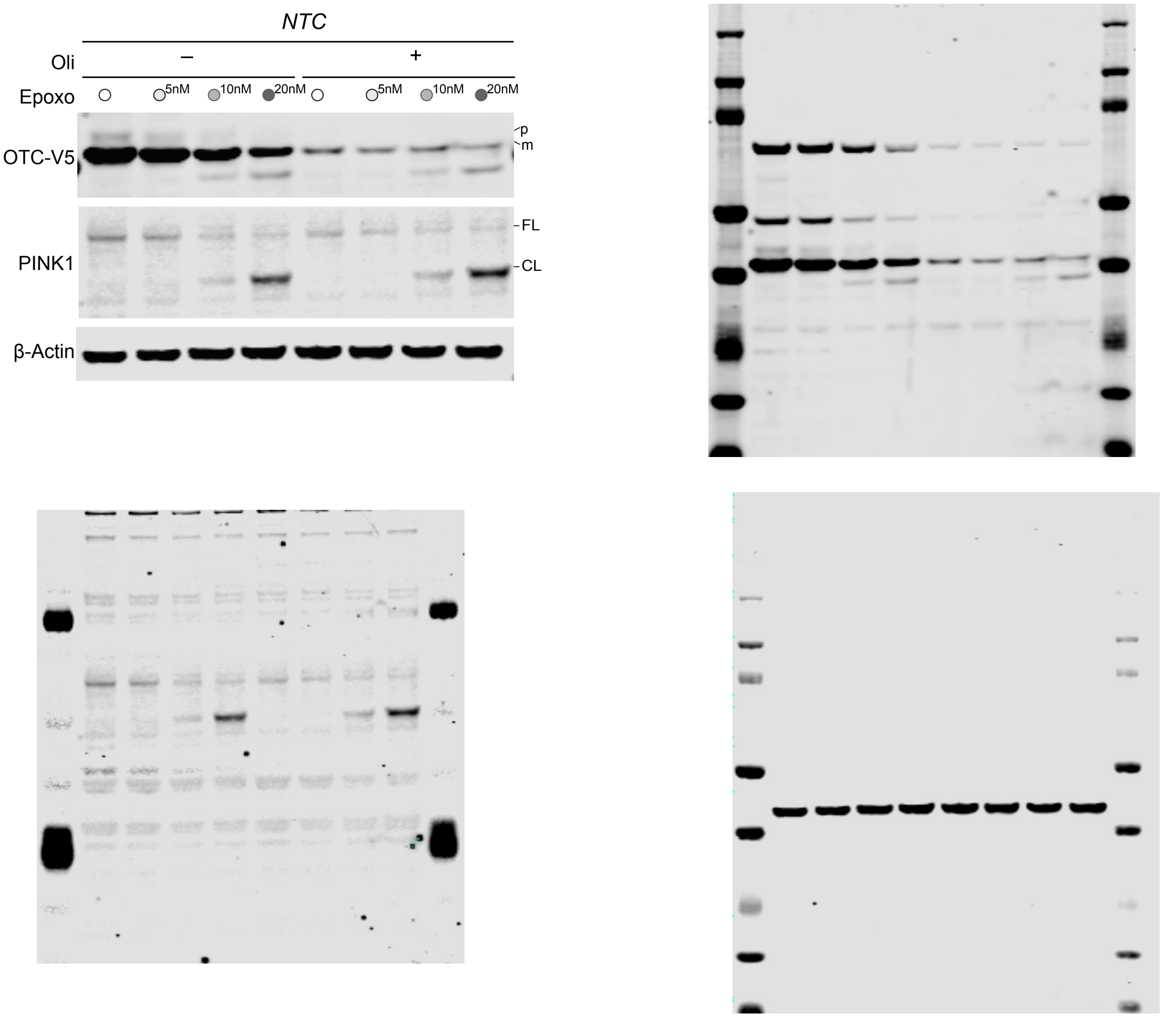

Supplementary Fig. 8a

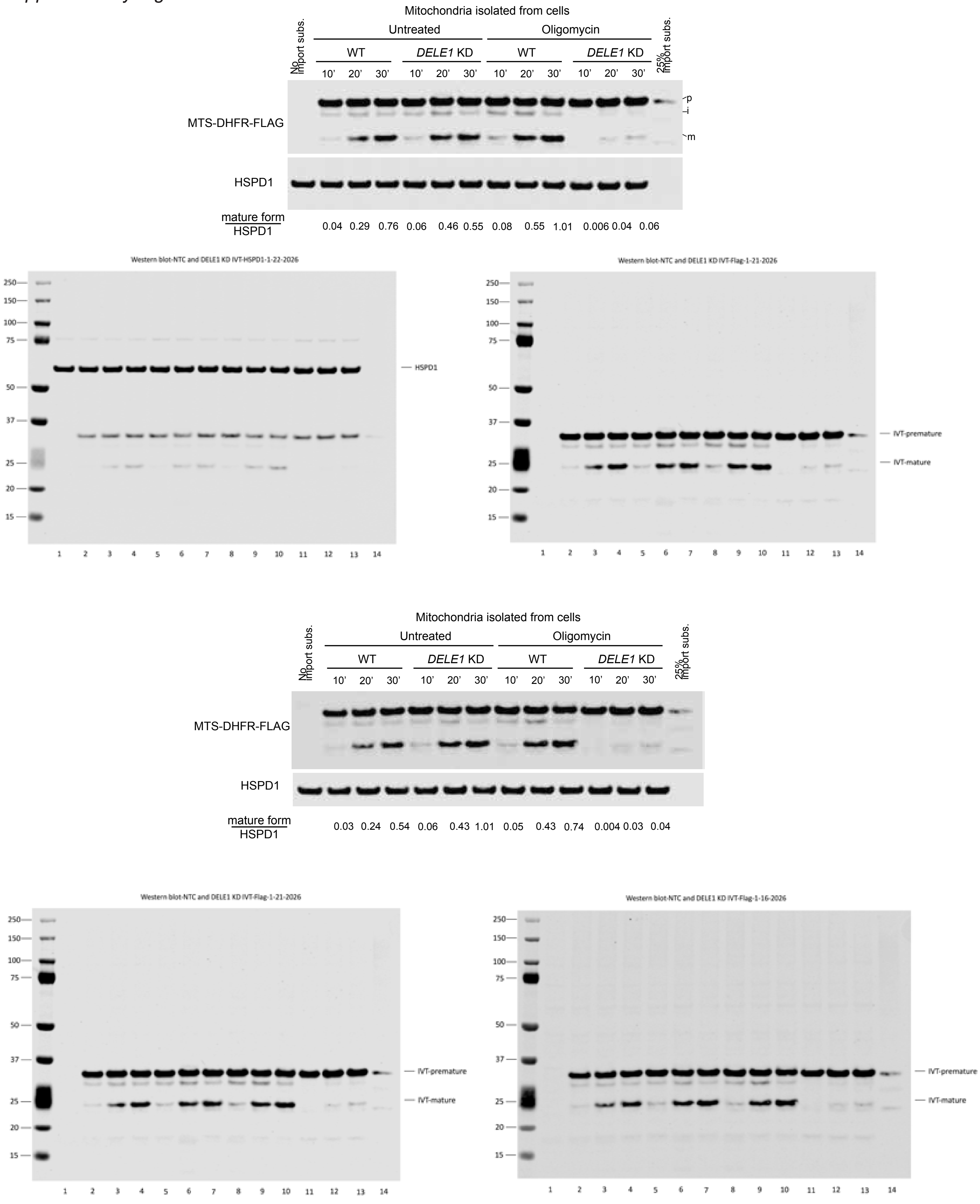

Supplementary Fig. 8b

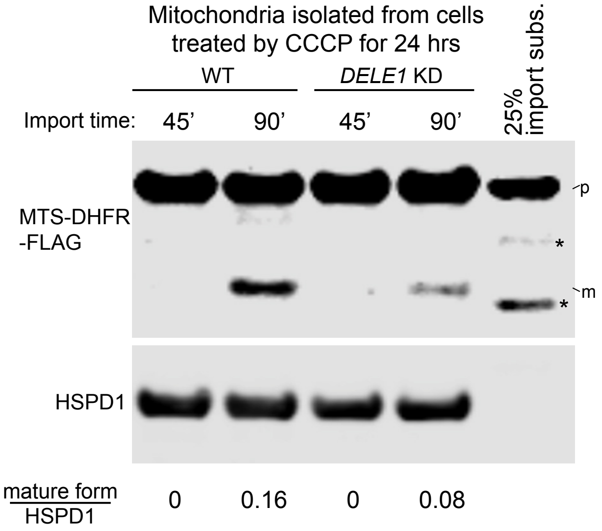

Western blot-IVT-cccp-Flag-9-24-2025

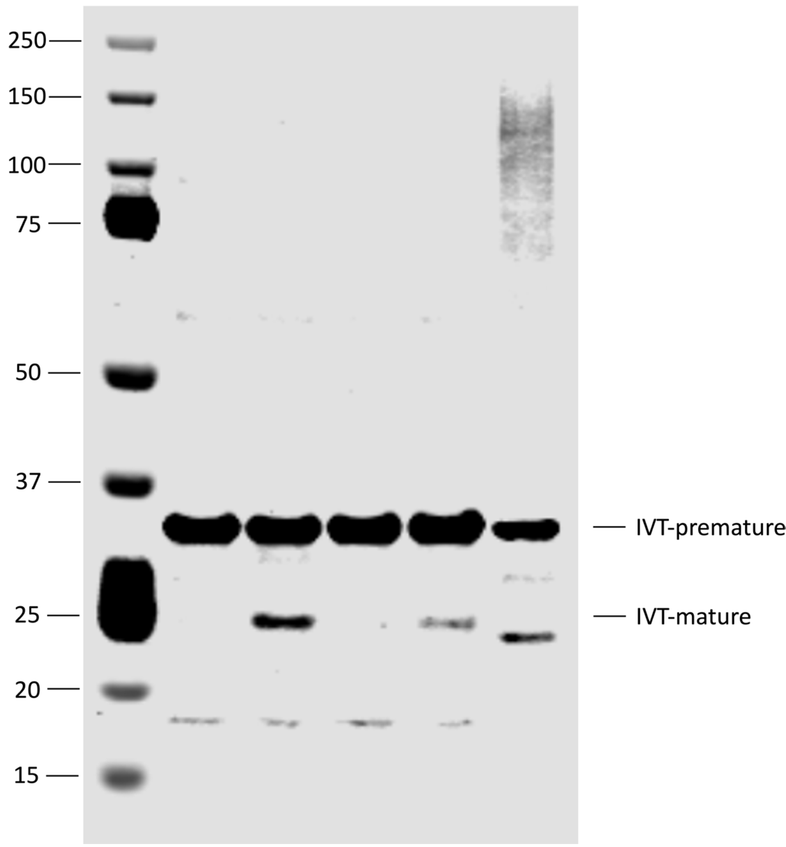

Western blot-IVT-cccp-HSPD1-9-25-2025

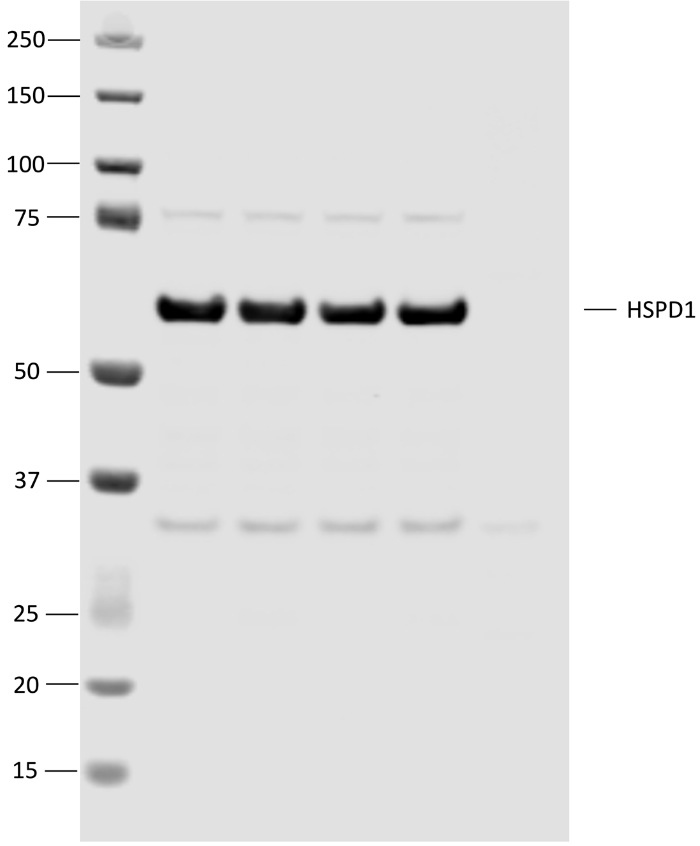

Supplementary Fig. 9b

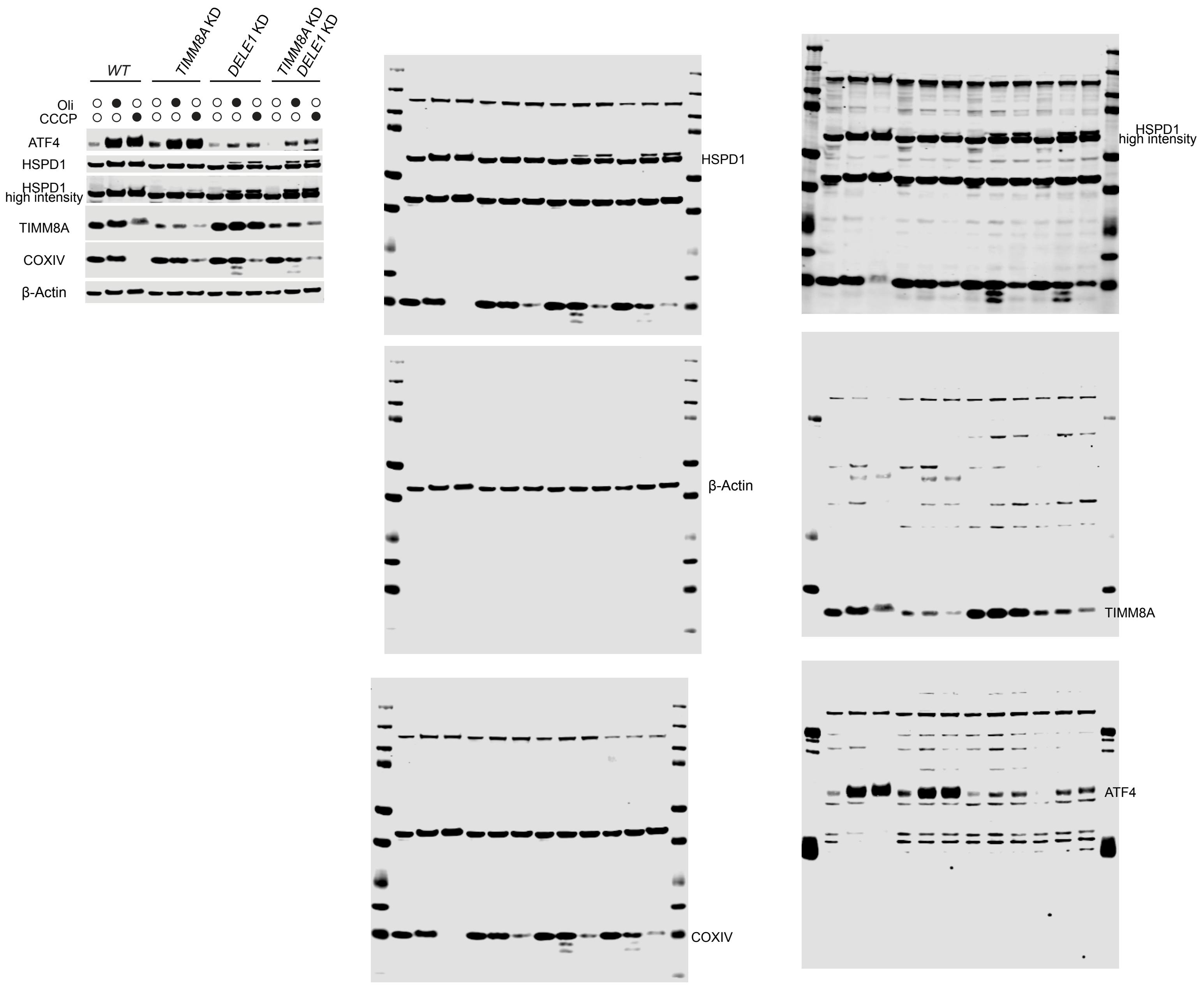

Supplementary Fig. 10a

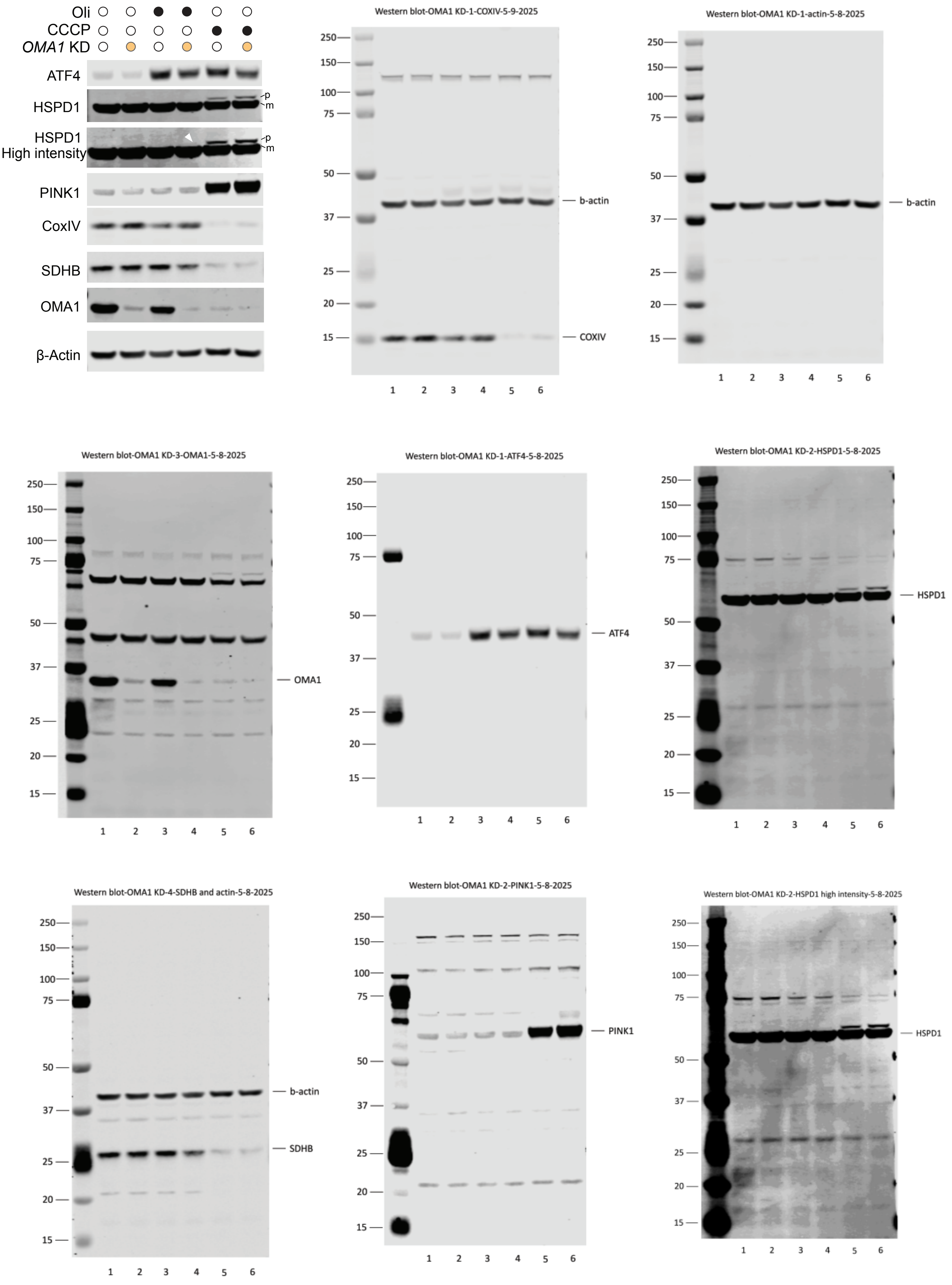

Extended Data Fig. 10d

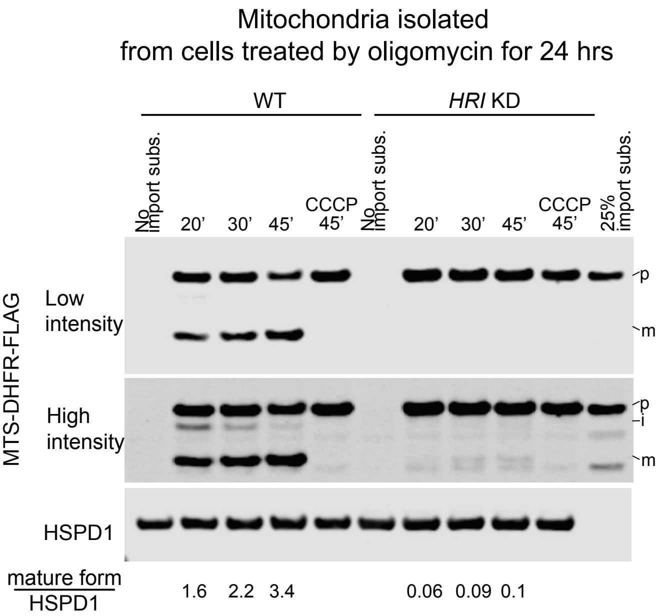

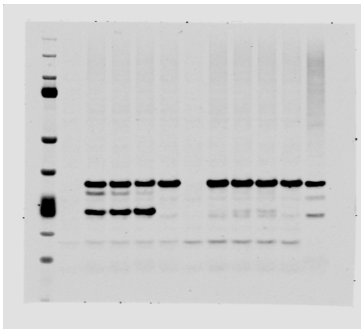

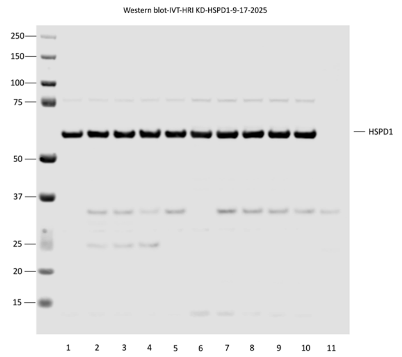

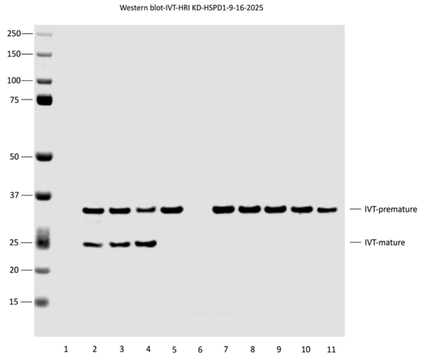

Extended Data Fig. 10e

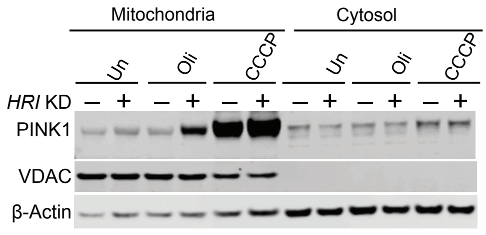

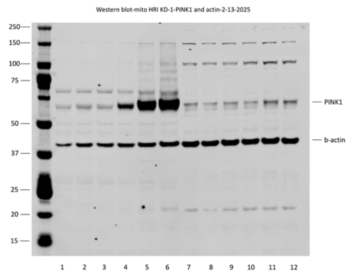

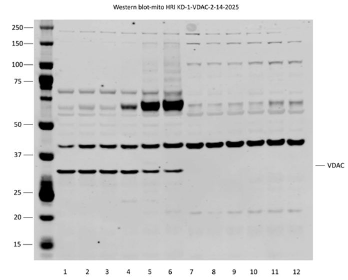

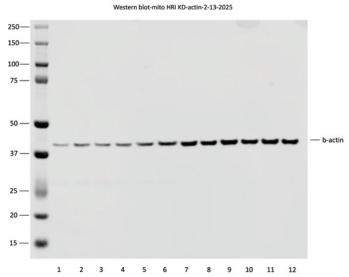

Extended Data Fig. 10f

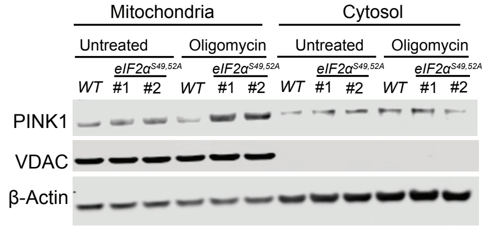

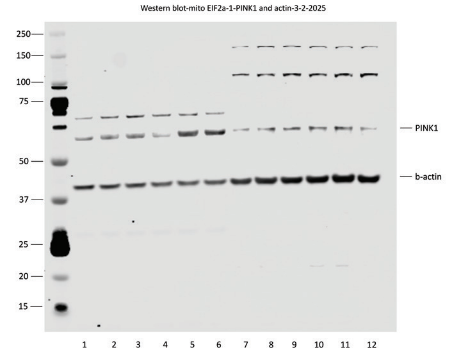

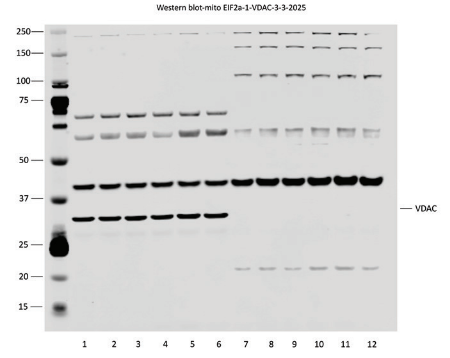

Extended Data Fig. 10g

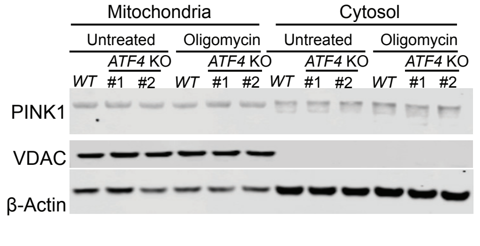

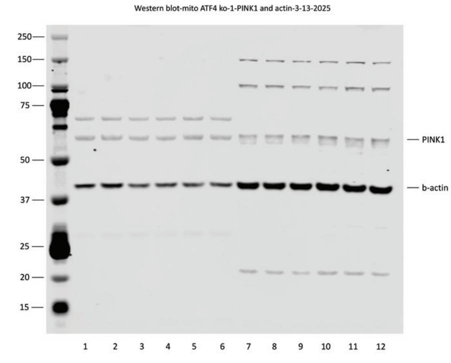

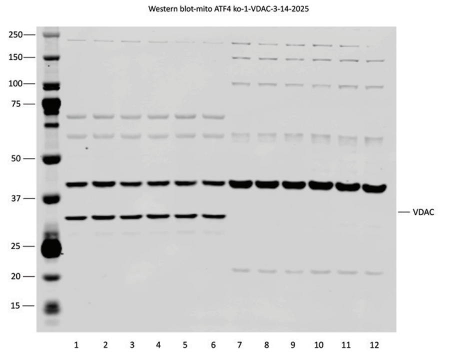

Supplementary Fig. 12a

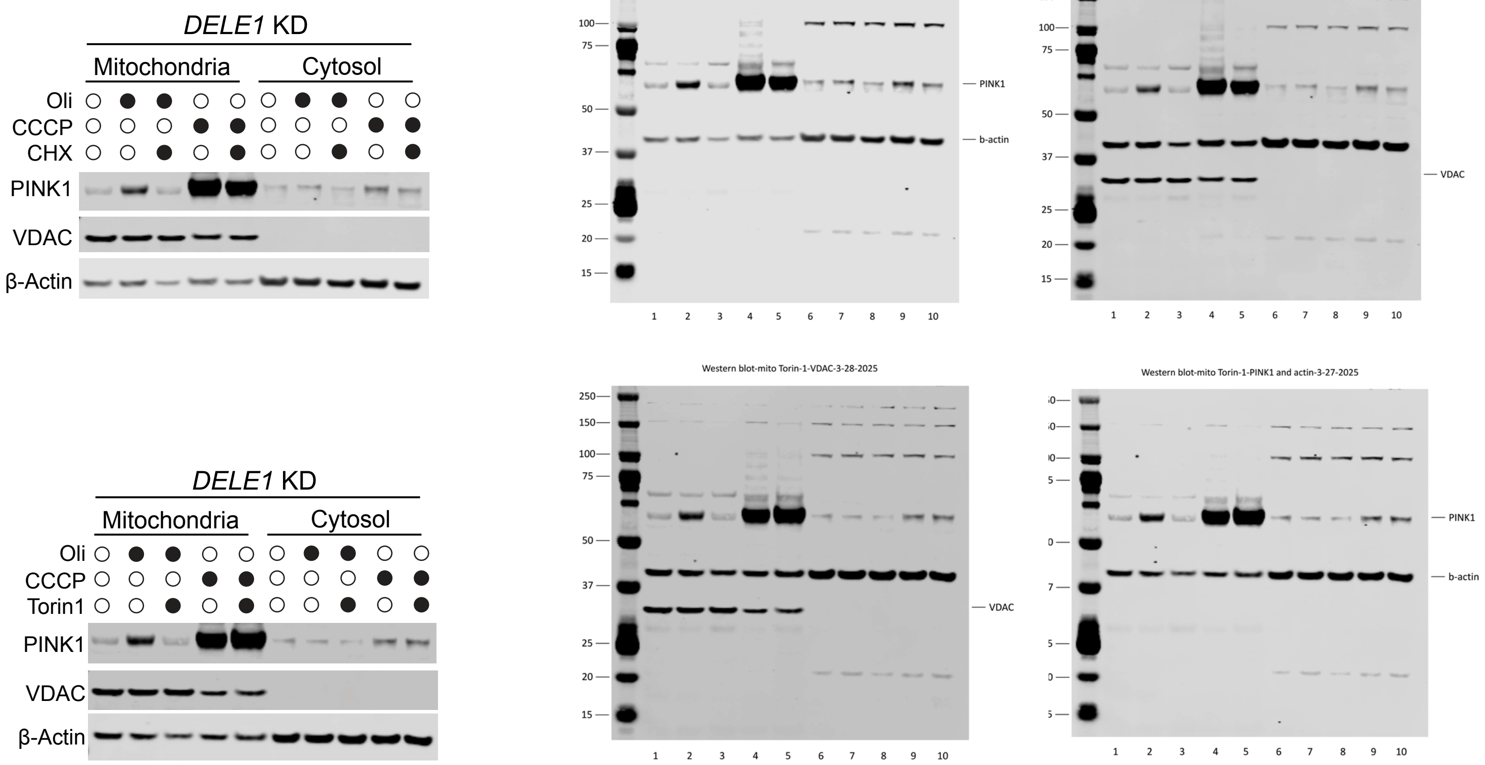

Supplementary Fig. 12e

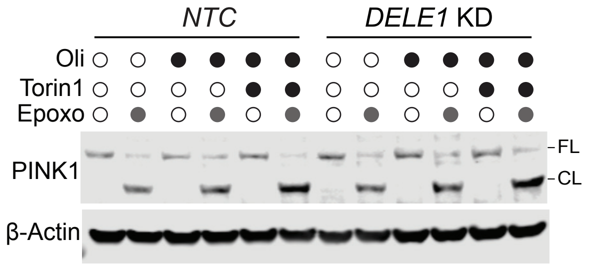

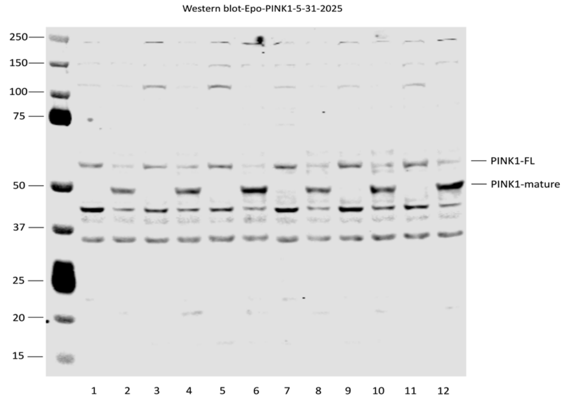

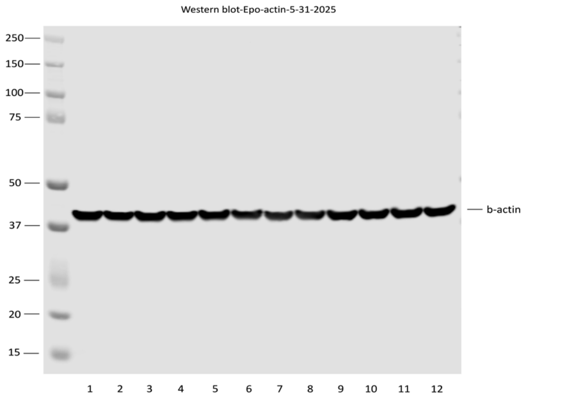

Supplementary Fig. 12f

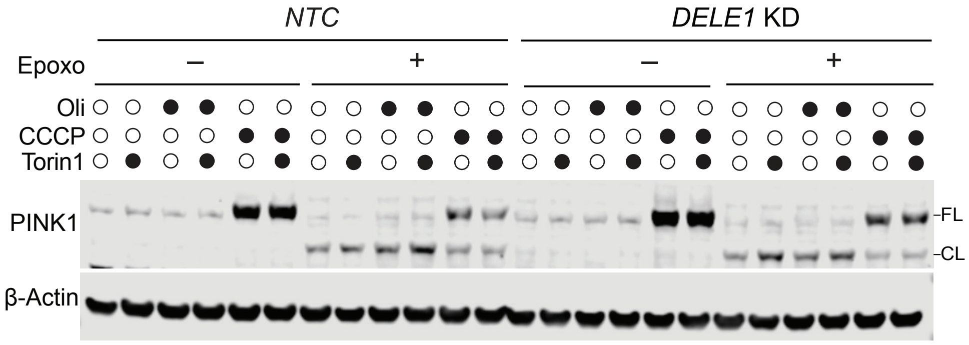

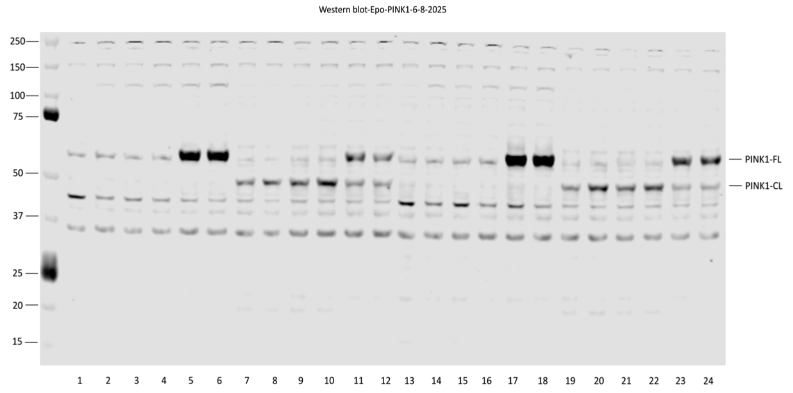

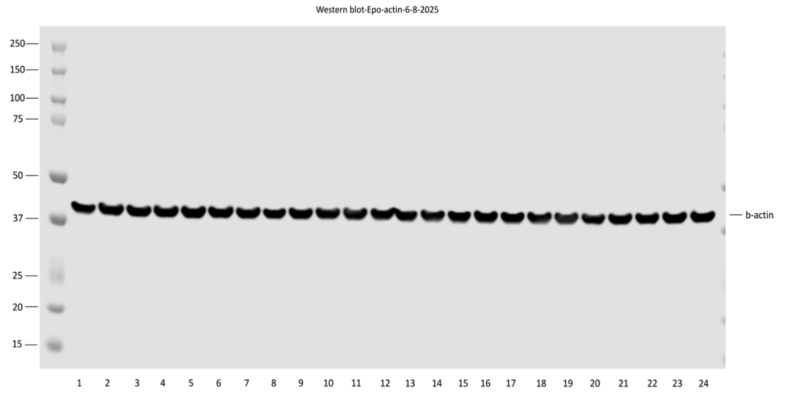

Supplementary Fig. 12g

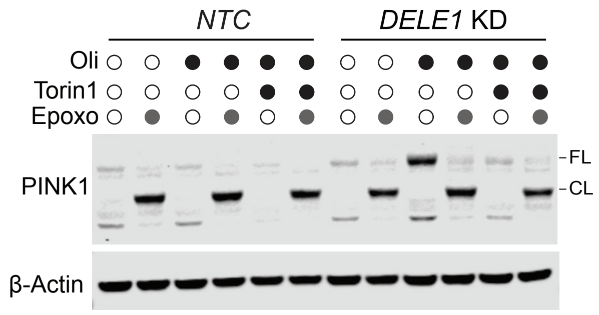

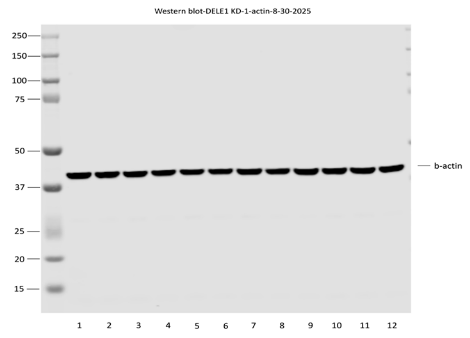

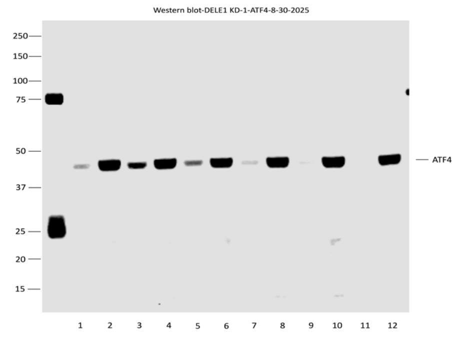

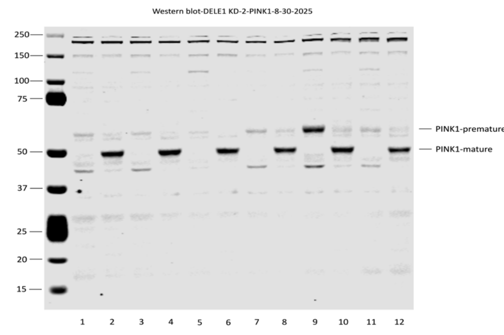

Supplementary Fig. 13

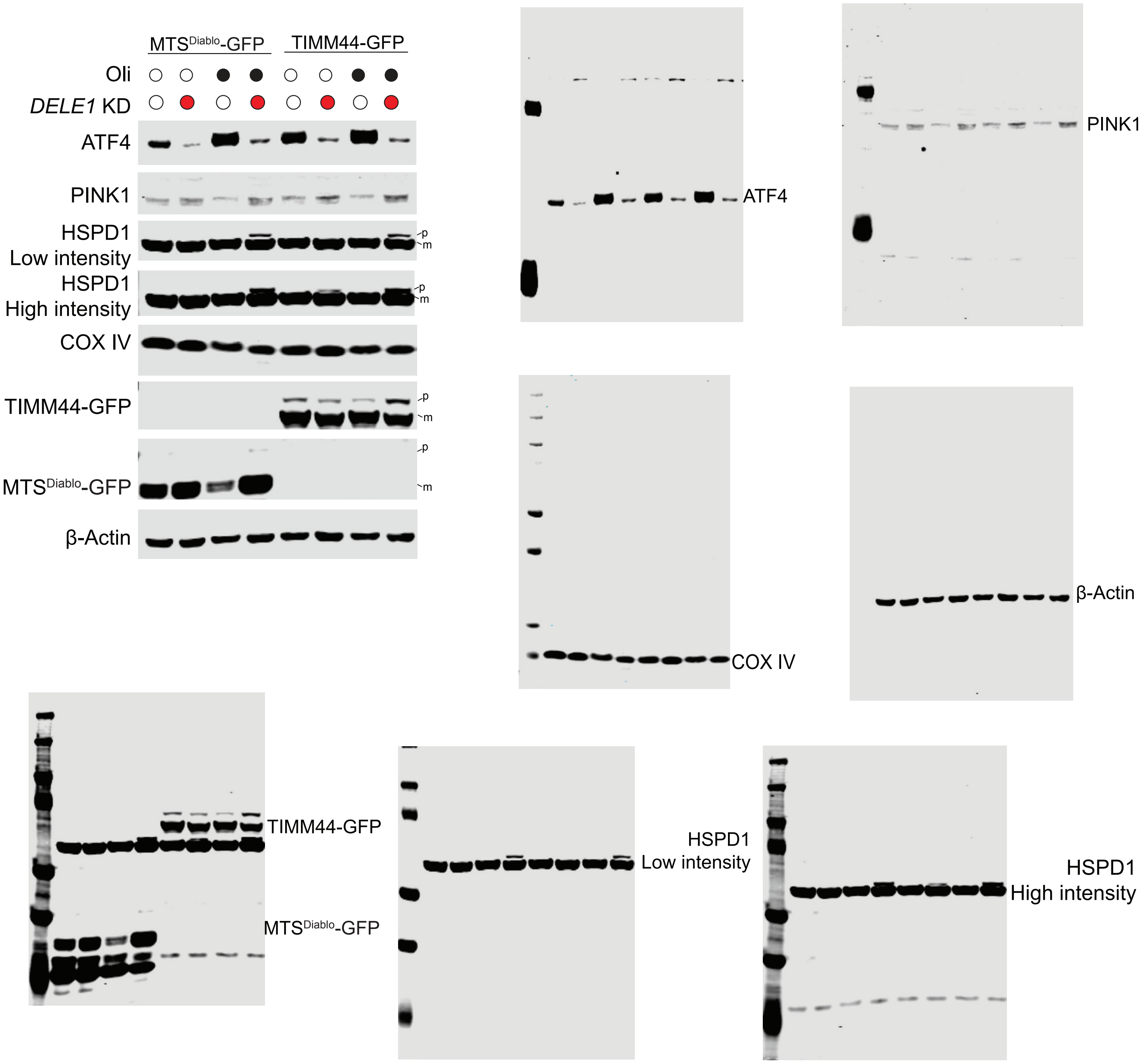

Supplementary Fig. 16b

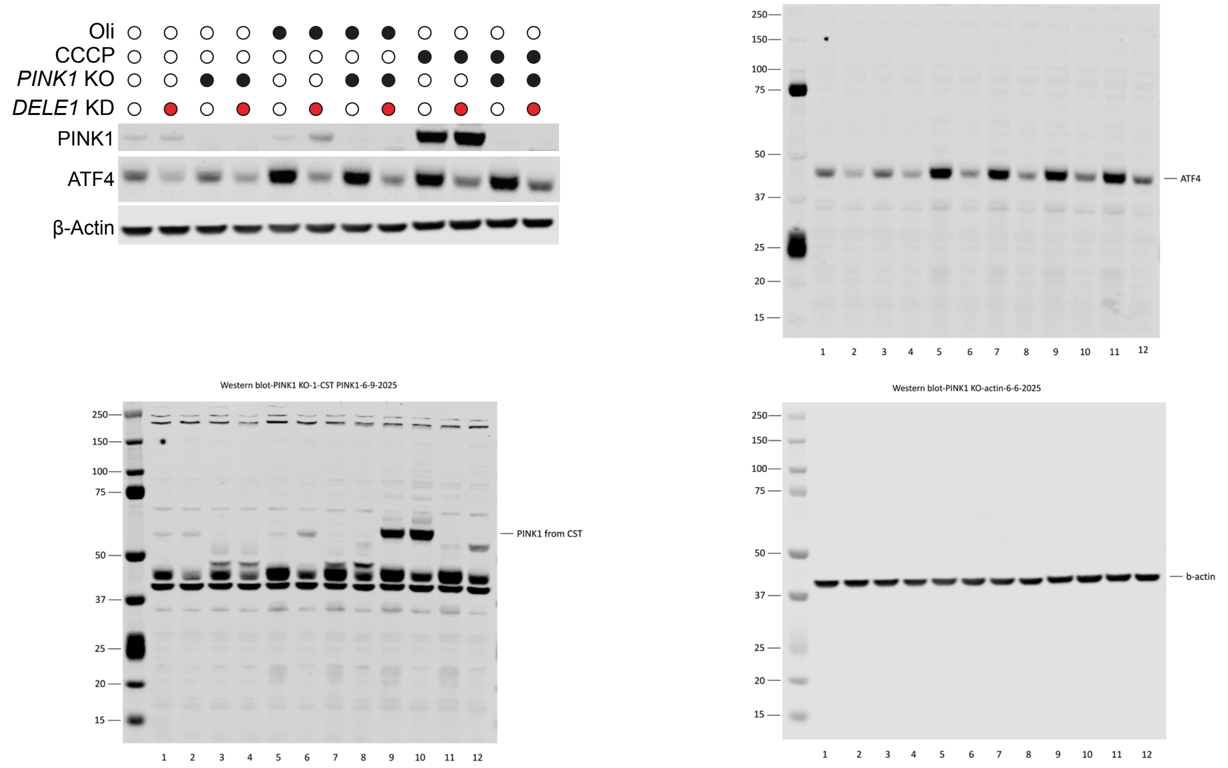

Supplementary Fig. 16c

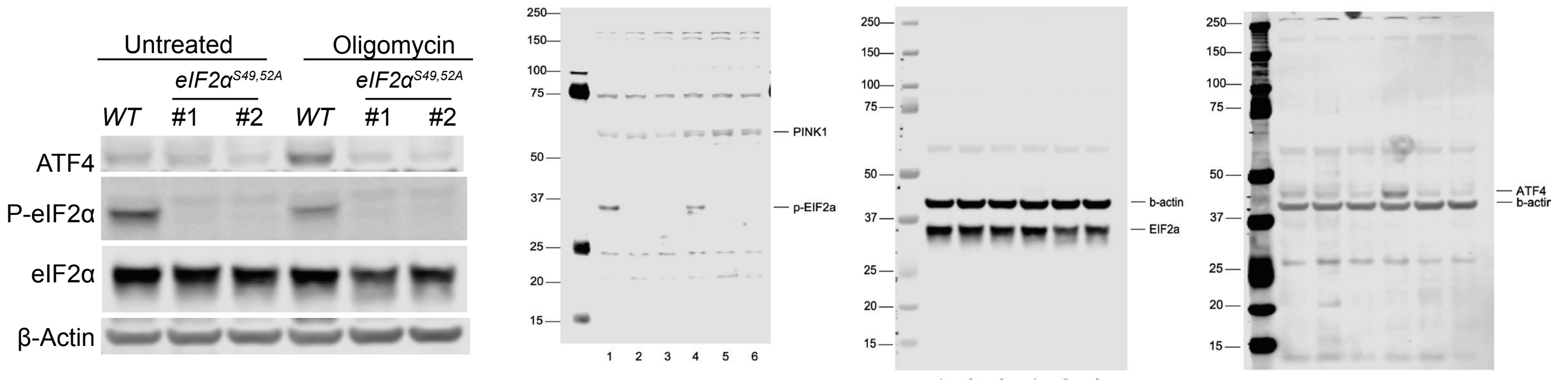

Supplementary Fig. 16d

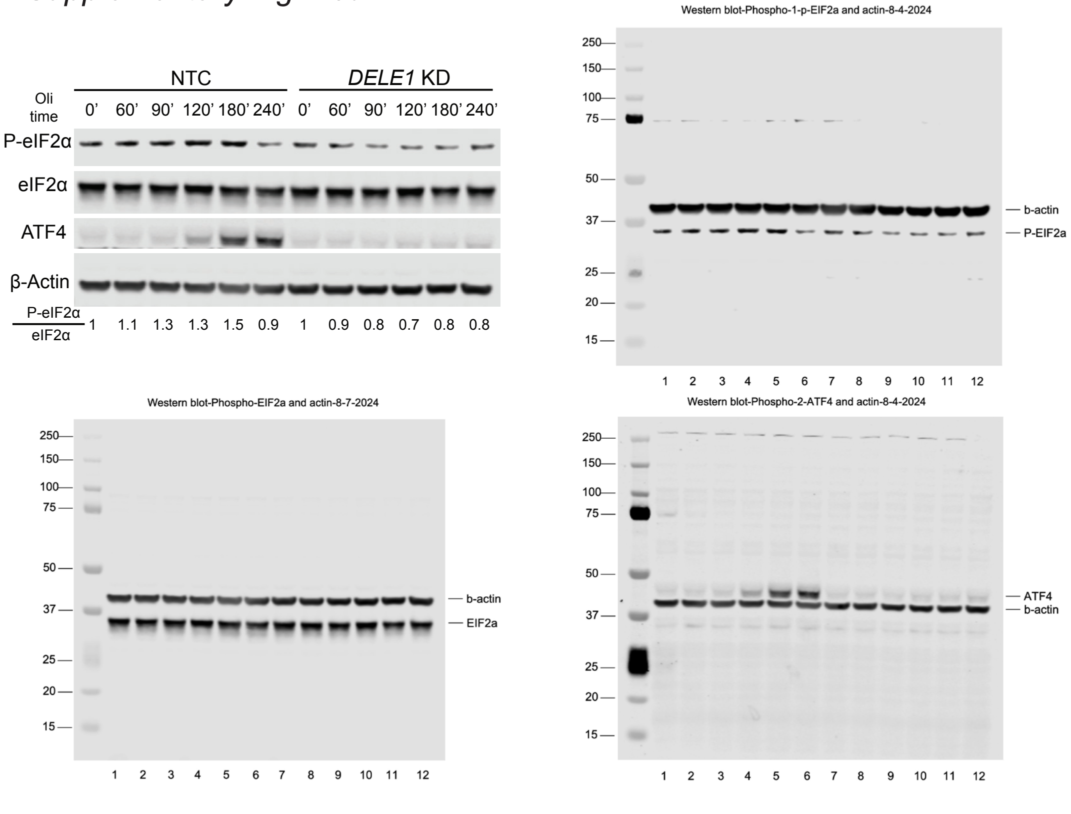

Supplementary Fig. 8a

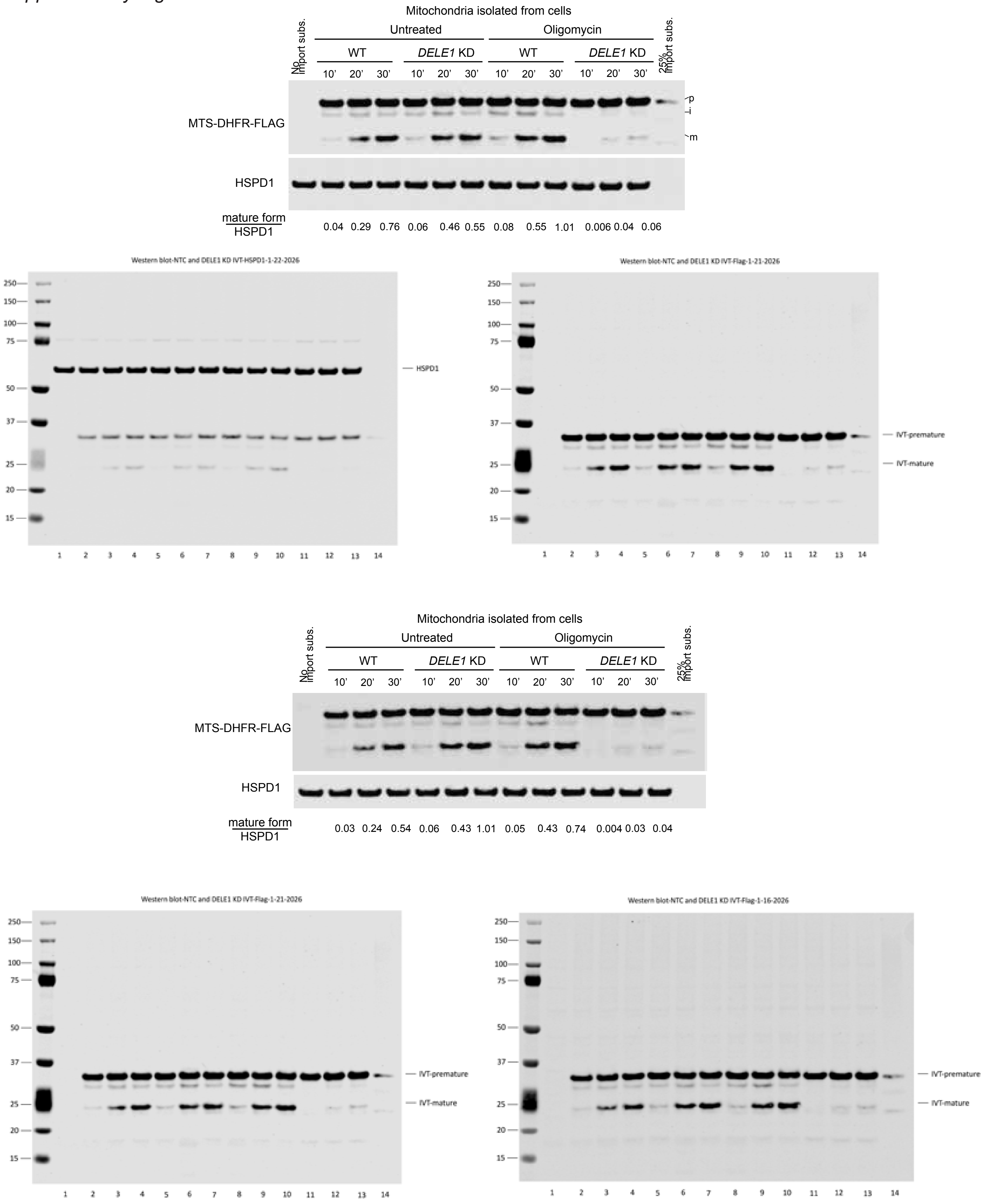

Supplement: Supplementary file 4 — Source data [file 41467_2026_71630_MOESM4_ESM.zip › Source_data_uncropped_WB.pdf]
